# COBRAme: A computational framework for genome-scale models of metabolism and gene expression

**DOI:** 10.1371/journal.pcbi.1006302

**Published:** 2018-07-05

**Authors:** Colton J. Lloyd, Ali Ebrahim, Laurence Yang, Zachary A. King, Edward Catoiu, Edward J. O’Brien, Joanne K. Liu, Bernhard O. Palsson

**Affiliations:** 1 Department of Bioengineering, University of California, San Diego, La Jolla, CA, United States of America; 2 Novo Nordisk Foundation Center for Biosustainability, Technical University of Denmark, Lyngby, Denmark; 3 Bioinformatics and Systems Biology Program, University of California, San Diego, La Jolla, CA, United States of America; 4 Department of Pediatrics, University of California, San Diego, La Jolla, CA, United States of America; University of Technology Sydney, AUSTRALIA

## Abstract

Genome-scale models of metabolism and macromolecular expression (ME-models) explicitly compute the optimal proteome composition of a growing cell. ME-models expand upon the well-established genome-scale models of metabolism (M-models), and they enable a new fundamental understanding of cellular growth. ME-models have increased predictive capabilities and accuracy due to their inclusion of the biosynthetic costs for the machinery of life, but they come with a significant increase in model size and complexity. This challenge results in models which are both difficult to compute and challenging to understand conceptually. As a result, ME-models exist for only two organisms (*Escherichia coli* and *Thermotoga maritima*) and are still used by relatively few researchers. To address these challenges, we have developed a new software framework called COBRAme for building and simulating ME-models. It is coded in Python and built on COBRApy, a popular platform for using M-models. COBRAme streamlines computation and analysis of ME-models. It provides tools to simplify constructing and editing ME-models to enable ME-model reconstructions for new organisms. We used COBRAme to reconstruct a condensed *E*. *coli* ME-model called *i*JL1678b-ME. This reformulated model gives functionally identical solutions to previous *E*. *coli* ME-models while using 1/6 the number of free variables and solving in less than 10 minutes, a marked improvement over the 6 hour solve time of previous ME-model formulations. Errors in previous ME-models were also corrected leading to 52 additional genes that must be expressed in *i*JL1678b-ME to grow aerobically in glucose minimal *in silico* media. This manuscript outlines the architecture of COBRAme and demonstrates how ME-models can be created, modified, and shared most efficiently using the new software framework.

This is a *PLOS Computational Biology* Software paper

## Introduction

Genome-scale metabolic models (M-models) have shown significant success predicting various aspects of cellular metabolism by integrating all of the experimentally determined metabolic reactions taking place in an organism of interest [1–4]. These predictions are enabled based on the stoichiometric and thermodynamic constraints of the organism’s metabolic reaction network and the metabolic interactions with the environment. M-models are capable of accurately predicting the metabolic capabilities of an organism, but they require defined substrate input constraints and empirical metabolite measurements to make predictions of its growth capabilities. Therefore, a focus of development in the field of genome-scale models has been to increase the scope and capabilities of genome-scale models [5].

**Fig 1:**
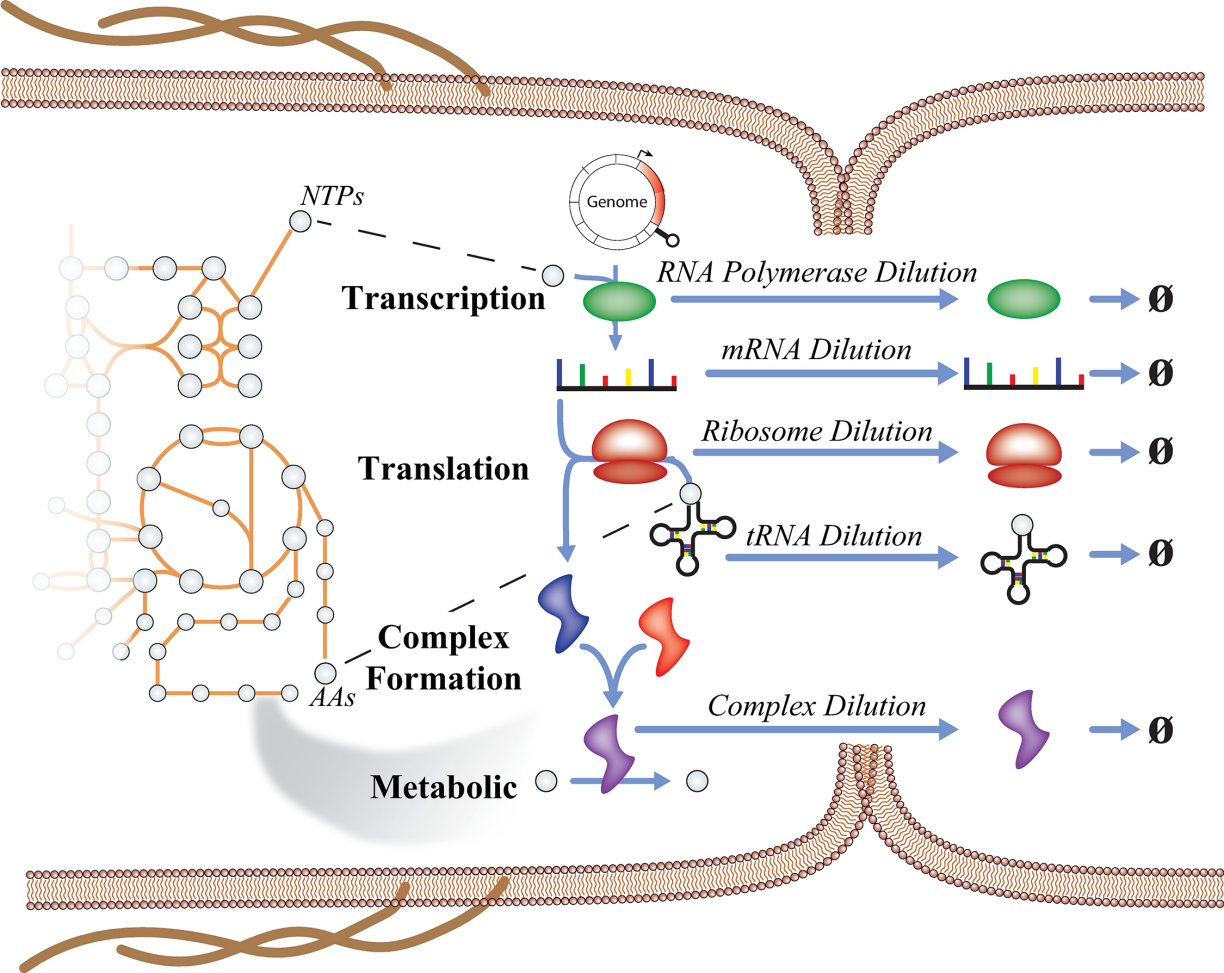
Multi-scale processes modeled in a ME-model depicted in a dividing *E*. *coli* cell. ME-models expand upon underlying M-models by explicitly accounting for the reactions involved in expressing genes that are required to catalyze enzymatic processes. The synthesis of each major macromolecule is coupled to the reaction that it is involved in by accounting for its dilution to daughter cells during cell division. Each dilution is a function of growth rate (μ).

Recently, M-models have been extended to include the synthesis of the gene expression machinery, enabling models to compute the entire metabolic and gene expression proteome in a growing cell [6–9]. These ME-models integrate Metabolism and Expression on the genome scale (**Fig 1**), and they are capable of explicitly computing a large percentage (up to 80% in some cases) of the proteome by mass in enterobacteria [10]. In other words, ME-models not only compute optimal metabolic flux states, as with M-models, but they additionally compute the optimal proteome composition required to sustain the metabolic phenotype. ME-models enable a wide range of new biological questions that can be investigated, including direct calculations of proteome allocation [11] to cellular processes, temperature dependent activity of the chaperone network [12], metabolic pathway usage, and the effects of membrane and volume constraints [7]. Furthermore, their ability to compute the optimal proteome abundances for a given condition make them ideal for mechanistically integrating transcriptomics and proteomics data.

So far ME-models have been constructed for only two organisms, *Thermotoga maritima* [8] and *Escherichia coli* K-12 MG1655 [6,7,9,13]. The slow pace of ME-model construction can be attributed to two basic challenges. First, ME-models are much slower to numerically solve than M-models; it takes 5 orders of magnitude more CPU time to solve *i*OL1650-ME [6] than it does the corresponding *i*JO1366 M-model [14] (~6 hrs for *i*OL1650-ME vs ~100 ms for *i*JO1366). Therefore, while M-models can be solved on personal computers, ME-models have required large clusters or supercomputers to parallelize simulations. Second, the large model sizes and complex structure have made analyzing and debugging the model difficult and time consuming. M-models can use generalized software tools [15–19], but each organism’s ME-model has required its own dedicated codebase and database schema, which makes advances for one organism’s model difficult to apply to another organism. Therefore, each organism’s ME-model has required dedicated person-years of effort.

We addressed these challenges by developing a computational framework called COBRAme for building, editing, simulating, and interpreting ME-models. COBRAme is written in Python and extends the widely used COBRApy software that only supports M-models [18]. COBRAme is designed to: 1) support any organism with an existing M-model; 2) use protocols and commands familiar to current users of COBRApy; 3) represent ME-models with an intuitive collection of Python classes; and 4) solve FBA simulations orders of magnitude faster than previous ME-models [6]. As a result of the above considerations, we hope that COBRAme and its associated tools will accelerate the development and use of models of metabolism and expression.

## Design and implementation

### Python

The COBRAme software (**S1 File**) is written entirely in Python 2.7+/3.5+ and requires the COBRApy [18] software package to enable full COBRA model functionality. Additionally, COBRAme requires the SymPy Python module [20] in order to handle the symbolic variable representing cellular growth rate (μ), which participates as a member of many stoichiometric coefficients in the ME-model. The BioPython package [21] is used by COBRAme to construct transcription, translation, and tRNA charging reactions for each gene product in the organism’s genbank genome annotation file. The ME-model is solved using the SoPlex [22,23] or qMINOS [24] solvers via APIs written in Python and included as part of this project. Further, the ECOLIme Python package is included in this work (**S2 File**) and contains information pertaining to *E*. *coli* gene expression and scripts to build *i*JL1678b-ME starting with the *E*. *coli* metabolic model, *i*JO1366 [14]. ECOLIme can further act as a blueprint for ME-model reconstructions of new organisms.

### ME-model architecture

Constructing a ME-model requires assembling information pertaining to many different cellular processes. For instance, in order to construct a translation reaction for the ME-model, the sequence of the gene, the codon table for the organism, the tRNAs for each codon, ribosome translation rates, elongation factor usage, etc. must be incorporated. Further, several processes in the ME-model recur for many genes that are transcribed or translated, due to their template-like nature [13]. To address these challenges, the COBRAme ME-model was structured to compartmentalize information for individual cellular processes. A key component of this approach was the separation of the ME-model into two major Python classes: the information storage vessels called **ProcessData** and the functional model reactions called **MEReaction**, which is analogous to the COBRApy Reaction.

#### ProcessData

COBRAme constructs ME-models that are composed of two major Python classes. The first of these is the ProcessData class, which is used to store information associated with a cellular process. The type of information contained in each ProcessData type is summarized in the **COBRAme Documentation (http://cobrame.readthedocs.io/, S4 File)**. This method of information storage has several advantages over alternatives such as establishing a database to query information as it is needed, which was the approach used to build previous ME-model versions. For example, this approach simplifies the dissemination of the information used to construct a ME-model given that the information can now be included as part of a published ME-model without requiring the user to install and populate a database. Further, this gives the ability to compartmentalize the information based on which cellular processes it elucidates. By storing this information in Python objects, methods can be implemented to further allow data contained in each ProcessData instance to be manipulated. This approach also reduces error by enabling many aspects of the model to be computed using defined inputs in a consistent way. For example, the amino acid sequence for a protein can be dynamically computed and used to construct a TranslationReaction instance using a gene’s nucleotide sequence and codon table (**Table 1 and S5 File**).

**Table 1:** Overview of all ProcessData subclasses.

ProcessData Subclass	Information Contained	Example	Number in *i*JL1678b-ME
StoichiometricData	Metabolite stoichiometry of a metabolic reaction (often equivalent to M-model reaction)	HISTD	2282
ComplexData	Protein subunit stoichiometry of an enzyme complex as well as the modifications required for its activity	CPLX-153	1445
SubreactionData	Some processes occur in multiple steps (e.g. translation reactions) or require modifications. This class details the stoichiometry and catalytic enzyme associated with the process.	ala_addition_at_GCA or mod_2fe2s_c	353
TranscriptionData	Nucleotide sequence, RNA products, sigma factor usage, etc. for a given transcription unit	TU00001_from_RpoD_mono	1447
TranslationData	Subreactions (tRNA mediated amino acid additions), sequence of mRNA/protein, etc. for a given mRNA being translated	b2020	1569
tRNAData	Codon, amino acid, tRNA, and modifications required to make a functioning tRNA	tRNA_b0202_AUU	158
TranslocationData	Keff, enzymes, and metabolite stoichiometry of a particular protein translocation pathway	srp_translocation	9
PostTranslationData	Translocation pathways, protein modifications (for lipoproteins), etc. required to produce a functioning protein.	translocation_protein_b0733	682
GenericData	List of complexes or metabolites that are redundant and represented as generics	generic_Tuf	11

#### MEReactions

ME-models are multiscale in nature, meaning they contain reactions that operate on dramatically different scales in time and space and whose reaction rates span ~15 orders of magnitude [25]. Fast reactions (e.g., metabolic) are coupled to slow reactions (e.g., complex formation) through coupling coefficients that determine the amount of macromolecule needed to catalyze particular reactions. To facilitate this coupling and to handle the unique characteristics of each major reaction type found in cell biology, the MEReaction Python class is used.

The MEReaction classes inherit all of the methods of a COBRApy Reaction. In addition to the functionality of the COBRApy Reaction, however, MEReactions contain methods to read and process the information contained in ProcessData objects and to update this information into a complete, functional reaction. In many cases, part of compiling a ME-model reaction also includes imposing the appropriate growth rate dependant coupling constraints (coupling constraints detailed in the **COBRAme Documentation** and Supplemental Text (**S8 File**)). These coupling constraints are imposed directly as part of the MEReaction’s update method and can vary depending on the reaction type. Since MEReactions are directly linked to the information used to construct them through the associated ProcessData, this codebase has the ability to easily query, edit, and update the information and metabolite stoichiometry constituting the MEReaction and therefore the edit model (**Table 2 and S5–S7 Files**). Examples of how this ME-model architecture can be leveraged to query and edit reaction information can be found in the **COBRAme Documentation**.

**Table 2:** ProcessData types used to construct each MEReaction type.

MEReaction Type	ProcessData Information Used	Number in *i*JL1678b-ME
MEReaction	None	2021
SummaryVariable	None	22
MetabolicReaction	**StoichiometericData**, SubreactionData, ComplexData	5266
ComplexFormation	**ComplexData**, SubreactionData	1445
TranslationReaction	**TranslationData**, SubreactionData	1569
TranscriptionReaction	**TranscriptionData**, SubreactionData	1447
PostTranslationReaction	**PostTranslationData**, TranslocationData, SubreactionData	682
tRNAChargingReaction	**tRNAData**, SubreactionData	158
GenericFormationReaction	**GenericData**	44

Most MEReaction types in COBRAme must be linked to at least one ProcessData instance that defines the core information underlying the reaction being represented. The required ProcessData for each reaction is listed in bold.

### ME-model reconstruction workflow

The ME-model of *E*. *coli* is reconstructed using the two Python packages presented here, COBRAme and ECOLIme. COBRAme contains the class definitions and necessary methods to facilitate building and editing a working ME-model. COBRAme is written to be organism-agnostic so that it can be applied to ME-models for any organism. ECOLIme contains the *E*. *coli* specific information (e.g., the *E*. *coli* ribosome composition) as well as functions required to process files containing *E*. *coli* reaction information (e.g., the text file containing transcription unit definitions) and associate them with the ME-model being constructed. Therefore, ECOLIme is required to assemble the reaction and gene expression information that comprises *i*JL1678b-ME. COBRAme, on the other hand, supplies the computational framework underlying the ME-model. The package composition along with further demonstrations of the utility of each of these packages is outlined in the **COBRAme Documentation**.

**Fig 2:**
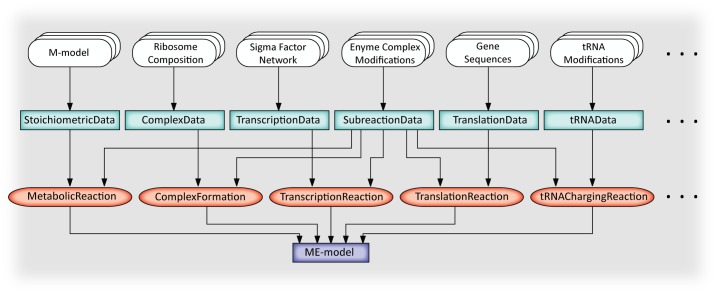
The flow of information from input data to the ME-model, as facilitated using the ‘build_me_model’ script. The ‘**build_me_model**’ workflow uses the ECOLIme package to load and process the *E*. *coli* M-model along with all supplied files containing information defining gene expression processes/reactions. This information is then used to populate the different ProcessData classes (shown in turquoise boxes) and link them to the appropriate MEReaction classes (shown in red ovals), all of which are defined in the COBRAme package. The entirety of the MEReactions comprise a working ME-model. Not all input data, ProcessData classes, and MEReaction classes are shown. For a complete list, reference the COBRAme Documentation.

The procedure used to build *i*JL1678b-ME using COBRAme and ECOLIme is presented in the building script, ‘**build_me_model**’ (**Fig 2**). This script goes through each of the major gene expression processes modeled in *i*JL1678b-ME and uses ECOLIme to load all the relevant information. Once the information is loaded, it is used to create and populate ProcessData instances associated with the information. Each of the ProcessData instances are then linked to the appropriate MEReaction instance and updated to form a functioning ME-model (**Fig 2**).

### Reformulating the *E*. *coli* ME-model

Significant efforts were made to simplify the ME-model while also optimizing the model size, modularity, and time required to solve. These included: **1)** reformulating the implementation of explicit coupling constraints (metabolites) and **2)** lumping major cellular processes such as transcription and translation into single ME-model reactions. Further, a number of updates, changes, and corrections have been made to the *E*. *coli* ME-model reconstruction which are detailed below and in the Supplemental Text (**S8 File**).

#### Macromolecular coupling

**Fig 3:**
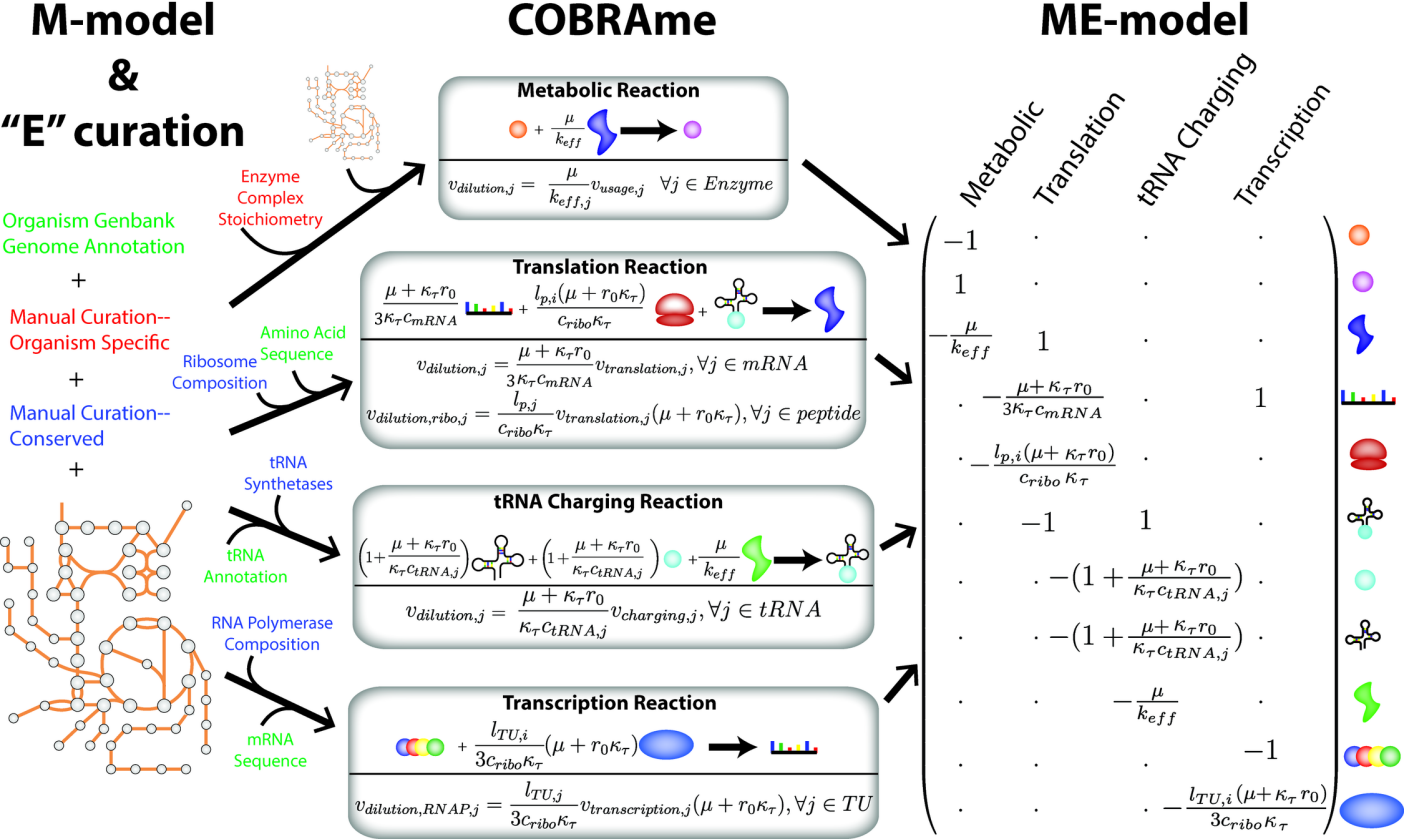
An overview of the COBRAme ME-model formulation. The previous ME-models implemented coupling constraints explicitly as model pseudo-metabolites. With COBRAme, instead of using explicit coupling constraints (metabolites), dilution of coupled macromolecules to the daughter cell is accounted for by applying a coupling coefficient directly in the reaction in which the macromolecule is used. For example, for the metabolic reaction shown above, a small amount (μ/k_eff_) of the catalyzing enzyme is consumed by the reaction in which it is involved. In other words, for a given amount of flux carried by the metabolic reaction, μ/k_eff_ * v_metabolic_reaction_ of the catalyzing enzyme must be synthesized. A subset of the major macromolecular coupling in *i*JL1678b-ME is also shown, along with their representation in the ME-matrix. Reference the COBRAme Documentation for derivations and further explanation of the coupling coefficients.

The largest mathematical difference between the original ME-model formulation [6] and COBRAme is the change in the macromolecular coupling implementation. Coupling coefficients dictate the amount of macromolecule synthesis flux that is required for the reaction catalyzed by that macromolecule to carry flux. They are derived based on the fact that, as a cell grows and divides, it dilutes macromolecules to its daughter cells. Therefore, coupling constraints have a general form of “μ/k_eff_” [6] (**Fig 3**), where μ is the growth rate and k_eff_ is the effective turnover rate of the process catalyzed by the macromolecule. While these are essential in a ME-model to couple together the various types of reactions, in previous model versions they inflated the number of metabolites and reactions contained in the ME-model (ME-matrix), resulting in longer solve times. COBRAme improves coupling constraint implementation by directly embedding macromolecule dilution coupling into its catalytic reaction (**Fig 3 and S8 File**).

A more thorough description of coupling constraint reformulation and implementation can be found in the **COBRAme Documentation** and the Supplemental Text (**S8 File**).

#### Reaction lumping

Splitting the model into ProcessData and MEReactions allows for a variety of model simplifications. For example, reactions that occur in a number of individual steps or sub-reactions (i.e., ribosome formation, translation, etc.) can be lumped into a single reaction. The single lumped MEReaction can be constructed by associating it with the multiple ProcessData instances that detail the individual sub-reactions involved in the overall reaction. All sub-reaction information is further accessible through the MEReaction instance itself which allows the information to be queried, edited, and updated throughout the reaction. If the sub-reaction participates in many different reactions, the sub-reaction changes can be applied throughout the entire model. This lumping has the obvious benefit of reducing the number of model reactions, thus shortening the solve time. Lumping intricate reactions has the added benefit of making the ME-model much more modular in nature. This simplifies the process of adding or removing new processes associated with the ME-model reaction. Examples of accessing and editing MEReactions through ProcessData can be found in the **COBRAme Documentation**.

#### Nonequivalent changes

Unlike the reformulations described above, some of the changes made in the COBRAme formulation purposefully changed the model in a nonequivalent way. One of the most significant differences was assigning a “dummy complex” monomer with a representative amino acid composition to act as the catalytic enzyme for “orphan” reactions. These are non-spontaneous reactions that do not have a known enzymatic catalyst. The previous ME-model formulations modeled these “orphan” reactions as spontaneous, causing a slight bias toward using these reactions since they did not have an associated protein expression cost. This was corrected in *i*JL1678b-ME. Additionally, in *i*JL1678b-ME, protein “carriers” (described in the **Supplemental Text** (S8 File)) like the acyl carrier protein are considered the catalysts of their transfer reactions. Therefore, *i*JL1678b-ME will require translation of these carriers in order for them to participate in the reactions in which they are involved, thus resulting in the expression of 52 more genes when simulating on glucose minimal media compared to *i*JL1678-ME.

Further, membrane surface area constraints imposed in *i*JL1678-ME were removed. This constraint limited the number of membrane proteins that could be expressed at a given growth rate. Protein competition for membrane space may play an important role in shaping *E*. *coli*’s metabolic phenotype, particularly when growing aerobically. Despite this, the constraint was removed to prevent the model from being over constrained when growing in non-glucose aerobic conditions, which could lead to unrealistic behavior. Removing this constraint makes *i*JL1678b-ME more flexible and applicable to more *in silico* conditions. Similarly, growth-dependent surface area calculations were used when imposing growth-dependent lipid synthesis demands, therefore they were also removed and replaced with demands identical to those defined in the *i*JO1366 biomass objective function. The protein translocation genes and pathways that were added when reconstructing *i*JL1678-ME, however, remain in *i*JL1678b-ME.

Additional corrections and changes made when reconstructing *i*JL1678b-ME are outlined in the Supplemental Text (**S8 File**).

### Optimization procedure

Unlike M-models, the stoichiometric matrix for each ME-model consists of numerous growth rate (μ) dependent metabolite coupling coefficients and variable bounds (**Figs 1 and 3**). This makes the ME-model nonlinear, meaning it cannot be solved as a normal LP like M-models. The ME-matrix, however, is quasi-convex [25], meaning that, for any feasible substituted μ, all smaller μ values will also be feasible. Therefore, the maximal feasible μ value can be determined by a binary search or bisection algorithm wherein successive linear programs are solved at different values of μ to find the largest value of μ that gives a feasible flux state, as done for *i*JL1678-ME and *i*OL1650-ME. For each optimization, the production of a representative “dummy complex” is maximized. In doing so, it allows the same algorithm to be used for both batch and nutrient limited growth, which required different procedures in *i*JL1678-ME and *i*OL1650-ME [6] (see **S8 File**).

While any linear programming solver supported by COBRApy [18] could have been used, ME-models are very ill-scaled [6], unlike M-models [26]. Therefore, two specialized solvers are used due to their extended numerical precision, thus ensuring acceptable numerical error: 1) qMINOS [23,24], which supports quad (128-bit) numerical precision, and 2) SoPlex [22], which supports “long double” (80-bit) numerical precision as well as iterative refinement in rational arithmetic to further reduce numerical error.

## Results and discussion

### Model overview

**Fig 4:**
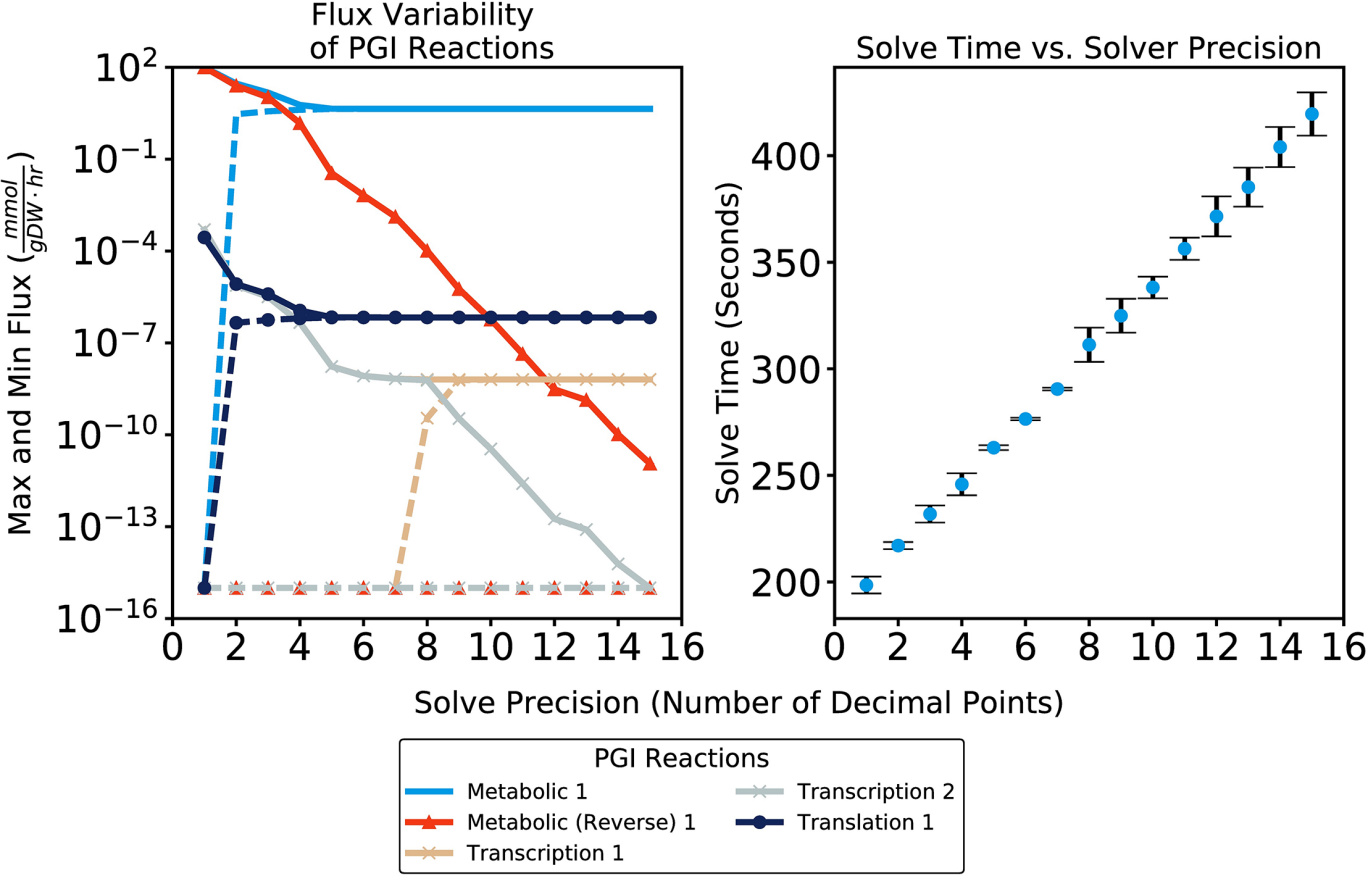
Flux variability analysis of reactions representing the expression of the Pgi enzyme and the PGI metabolic reaction. The variability becomes negligible (the max and min possible fluxes converge) for metabolic and translation fluxes when using a μ precision of 10^−5^ and for transcription fluxes when using a μ precision of 10^−15^. There are two transcription reactions for *pgi* since this gene can be transcribed using two different sigma factors. The lower limit of reaction flux values is set to 10^−15^ mmol • gDW^-1^ • hr^-1^ as this is close to the lowest value that can be accurately represented in double-precision floating-point in Python. Note the maximum reaction flux for the reverse direction of PGI does not drop to 10^−15^ mmol • gDW^-1^ • hr^-1^ by this μ precision. However, considering the general scale of metabolic reaction fluxes (see **Fig 5**), the maximum flux effectively drops to zero for practical purposes. High μ precision can be achieved without sizeable increases in total solve time using qMINOS. The ME-model simulations were repeated nine times for each precision and the error bars represent the standard deviation of the solve times.

The COBRAme framework was used to reconstruct a mass-balance checked, reformulated version of the *E*. *coli* K-12 MG1655 ME-model *i*JL1678-ME, called *i*JL1678b-ME (**S3 File**). This produced a model with 12,655 reactions and 7,031 metabolites (**S6 and S7 Files**), a marked improvement over *i*JL1678-ME which contained 79,871 reactions and 70,751 metabolites. As a result, *i*JL1678b-ME has a stoichiometric matrix with ~85% fewer columns and ~90% fewer rows than *i*OL1650-ME. This dramatically speeds up the solving procedure and allows processes such as iterative refinement, which uses rational arithmetic and is unsuited for fast vectorized SIMD operations, to become feasible for fast and accurate solutions (**Fig 4, Fig B in S8 File**).

**Fig 5:**
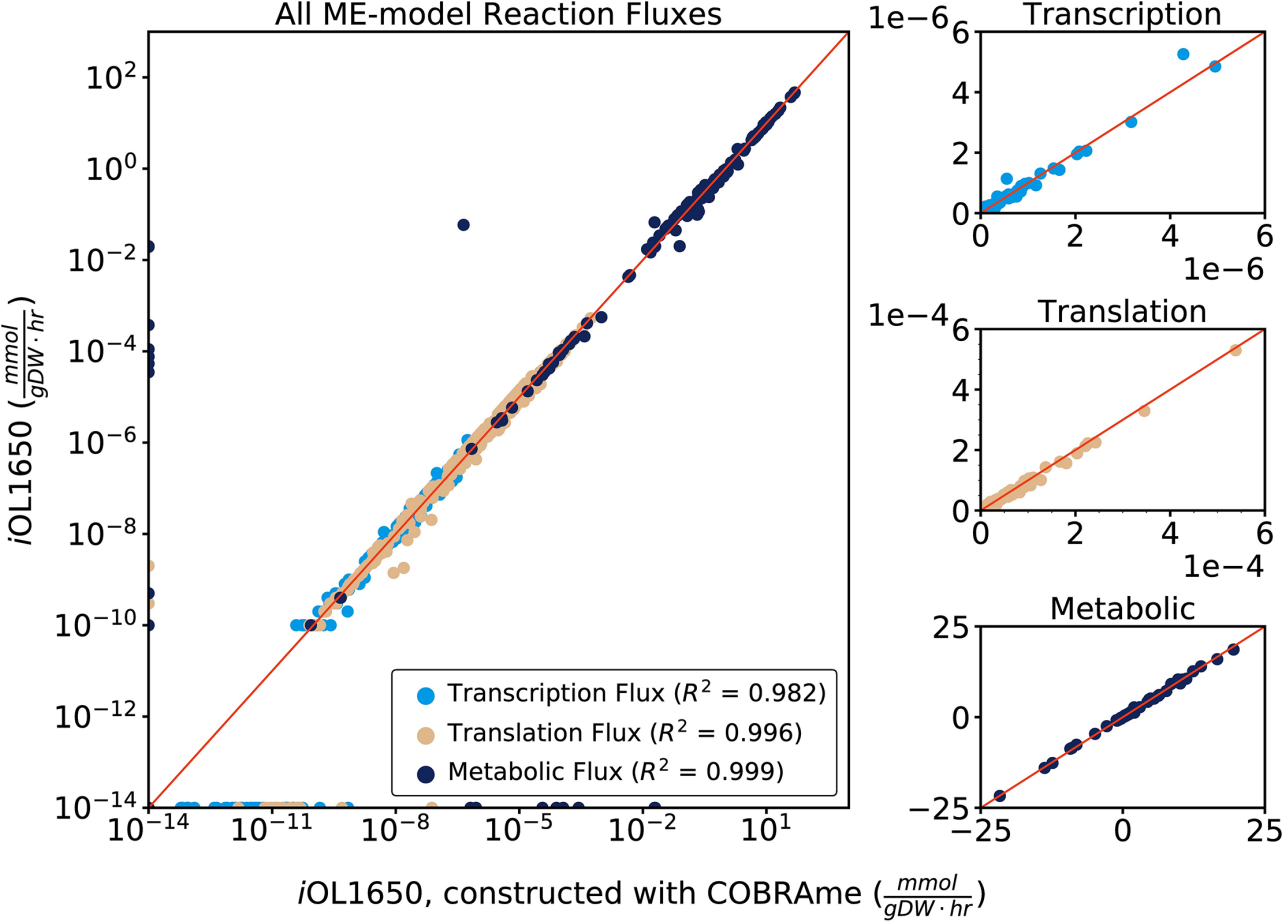
Comparison of the simulated fluxes of *i*OL1650-ME to the COBRAme-constructed version of the same model at transcription, translation, and metabolic flux scales. All fluxes are shown in pairwise comparison on the left using a log scale axis. The fluxes are separated into the major reaction types to be shown on a linear axis on the right. In order for fluxes of 0 mmol/gDW/hr to appear, 0 fluxes have been replaced with 10^−14^ on the left plot. At each level, the models provided comparable flux predictions (R^2^>0.98). The models cannot be expected to give completely identical flux predictions due to the ME-model updates outlined in **Nonequivalent Changes**. Since *i*JL1678b-ME does not contain membrane surface area constraints, *i*OL1650-ME was used for comparison.

*i*OL1650-ME, constructed using COBRAme, was simulated in glucose aerobic minimal media *in silico* conditions and compared against simulations from the previous *i*OL1650-ME version. Both simulations were ran using a selection of k_eff_ parameters that were fit to proteomics data obtained from *E*. *coli* grown in multiple conditions [27]. The new model version gave similar fluxes (R^2^>.98) when comparing model solutions on a transcription, translation, and metabolic level (**Fig 5**), suggesting that the two models are practically identical, computationally. The reformulated ME-model cannot be expected to give completely identical solutions to *i*OL1650-ME due to some of the nonequivalent changes and model corrections described in **Nonequivalent Changes**. Particularly, the RNA degradosome and RNA excision machinery was slightly under-expressed due to the change in stable RNA excision handling, described in the Supplemental Text (**S8 File**).

Computational essentiality predictions for both *i*JL1678b-ME and *i*OL1650-ME were compared against a genome-wide essentiality screen of single gene knockouts grown in glucose M9 minimal media [28]. Due to the corrections described above and in the Supplemental Text (**S8 File**), *i*JL1678b-ME displayed improved gene essentiality predictions when comparing essentiality for the 1539 proteins also modeled in *i*OL1650. The bulk of these improvements stem from modeling the expression of enzyme “carriers” as mentioned in **Nonequivalent Changes.** This correction led to a 35 gene decrease in the number of false positive predictions made by *i*JL1678b-ME, but also led to a 22 gene increase in true positives. Overall, the accuracy of the model improved from 86.6% to 87.5%. Further, the Matthews Correlation Coefficient [29], a machine learning metric to gauge the performance of binary classifiers, saw an increase of 7% from 0.616 to 0.659 (**Table 3**).

**Table 3:** Summary of essentiality predictions for the 1539 proteins modeled in both *i*JL1678b-ME and *i*OL1650-ME.

		Experimental	
		Essential	Nonessential
***i*JL1678b-ME**	**Essential**	1070 (69.5%)	109 (7.1%)
	**Nonessential**	84 (5.5%)	276 (17.9)
***i*OL1650-ME**	**Essential**	1092 (71.0%)	87 (5.7%)
	**Nonessential**	119 (7.7%)	241 (15.4%)

Predictions of essentiality are from a genome wide screen of Keio collection [30] knockouts grown on glucose M9 minimal media [28].

Beyond performance and predictive improvements, the reformulations and reduced size make *i*JL1678b-ME more understandable to the user. By lumping cellular processes into individual model reactions, the structure of each ME-model reaction is able to more closely resemble known reactions the user will recognize. For instance, the translation of a given gene, <gene_id>, occurs in a single model reaction, “translation_<gene_id>,” where all components and coupling constraints are applied in one place (**Fig 3**) as opposed to occurring in multiple, distinct reactions. In addition to being more easily understandable by the user, the reformulation makes the model more amenable to visualization tools like Escher [19], further easing the process of interpreting simulation results.

## Availability and future directions

Both the COBRAme and ECOLIme software packages are required to construct *i*JL1678b-ME and are currently available on the Systems Biology Research Group’s Github page (https://github.com/SBRG). Installation procedures as well as all necessary documentation required to build, simulate, and edit ME-models are present in the repository READMEs. The qMINOS solver [24] is also freely available for academic use. Instructions for installing and using the solver can be found as part of the solveme package [25]. Alternatively, the SoPlex solver can be found at (http://soplex.zib.de/) and is freely available to academic institutions. The soplex_cython package contains instructions to compile the soplex solver with 80-bit precision capabilities along with the necessary code required to solve *i*JL1678b-ME with SoPlex. Builds of COBRAme, ECOLIme, the qMINOS solver, and all dependencies can be further obtained from Docker Hub (https://hub.docker.com/r/sbrg/cobrame/). The scripts and instructions for locally building Docker images that include the above software as well as SoPlex can be found on the COBRAme GitHub repository. This allows researchers to easily install and use ME-models regardless of platform and enables cloud computing platforms for ME-model simulations. These software packages will be actively maintained and continuously improved. The COBRAme documentation can be found on readthedocs (https://cobrame.readthedocs.io/). The scripts, data, and instructions needed to reproduce the presented results can be found in the **S3 File** or at https://github.com/coltonlloyd/cobrame_supplement.

### Enable new ME-model reconstructions

We anticipate that the presented software tools will facilitate the reconstruction of many new ME-models beyond *i*JL1678b-ME for *Escherichia coli* K-12 MG1655. While the COBRAme code was constructed to be readily applicable to many different organisms, it is likely that some organisms will require additional features for their ME-model reconstruction that we did not originally anticipate. It is our priority to continue to update and improve the code to enhance its utility to model new, diverse organisms. Future efforts will also be made to create standards to govern how ME-models are reconstructed and shared within the scientific community. This will include working with the systems biology community to develop SBML [31,32] standards capable of encoding the information required to reproducibly build and simulate ME-models.

## Supporting information

S1 File The COBRAme source code. The COBRAme version 0.0.9 source code. The latest version of COBRAme can be downloaded from https://github.com/SBRG/cobrame.
(ZIP)

S2 File The ECOLIme source code. The ECOLIme version 0.0.9 source code. The latest version of ECOLIme can be downloaded from https://github.com/SBRG/ecolime.
(ZIP)

S3 File Scripts used to produce all figures and tables. All results outlined in the manuscript can be reproduced by following the instructions in the README. A JSON version of iJL1678b-ME is also contained in this file. Alternatively, these scripts can be found at https://github.com/coltonlloyd/cobrame_supplement.
(ZIP)

S4 File The documentation for COBRAme version 0.0.9. The latest version of the documentation can be found at https://cobrame.readthedocs.io.
(PDF)

S5 File The ProcessData in *i*JL1678b-ME. Summary of the information contained in all ProcessData types used in the ME-model. Descriptions of what each ProcessData class represents can be found in **Tables 1 and 2**.
(XLSX)

S6 File The MEReactions in *i*JL1678b-ME. Summary of the attributes of each reaction type used in the COBRAme ME-model.
(XLSX)

S7 File The metabolites in *i*JL1678b-ME. Summary of the attributes of each metabolite type used in the COBRAme ME-model.
(XLSX)

S8 File Supplemental Text and figures. Includes additional information regarding ME-model updates and reformulations along with some additional analysis.
(PDF)
