# Supplementary figures and images for: COBRAme: A computational framework for genome-scale models of metabolism and gene expression
Source: PLoS Comput Biol. 2018 Jul 5;14(7):e1006302. doi: 10.1371/journal.pcbi.1006302 (PMC6049947; doi:10.1371/journal.pcbi.1006302)

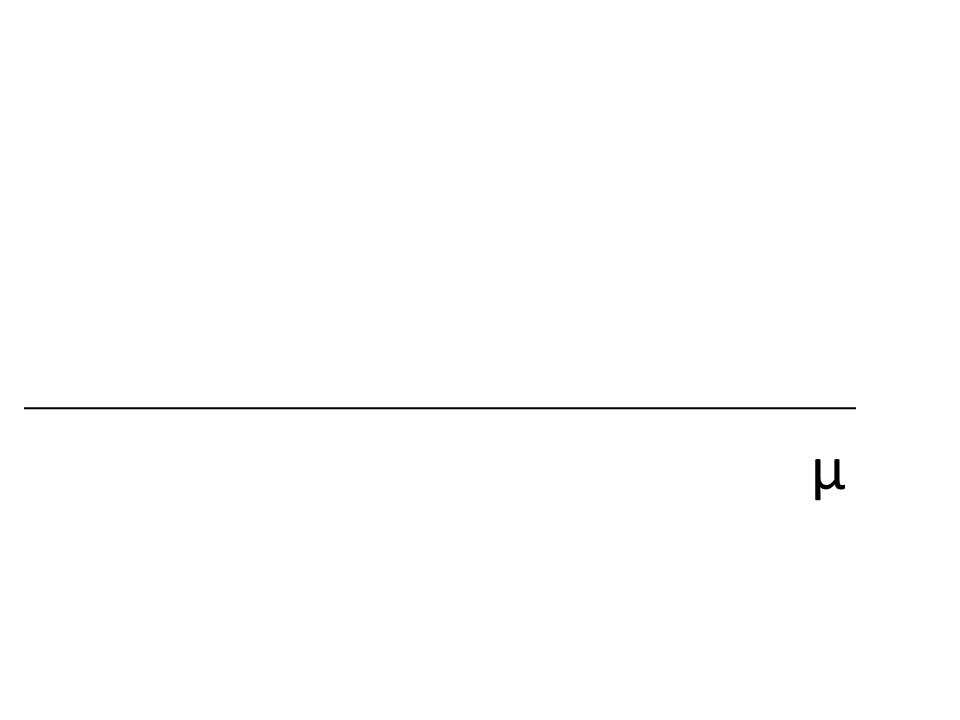

Supplement: S1 File — The COBRAme version 0.0.9 source code. The latest version of COBRAme can be downloaded from https://github.com/SBRG/cobrame. (ZIP) [file pcbi.1006302.s001.zip › S1_File/docs/_static/binary_search.gif]

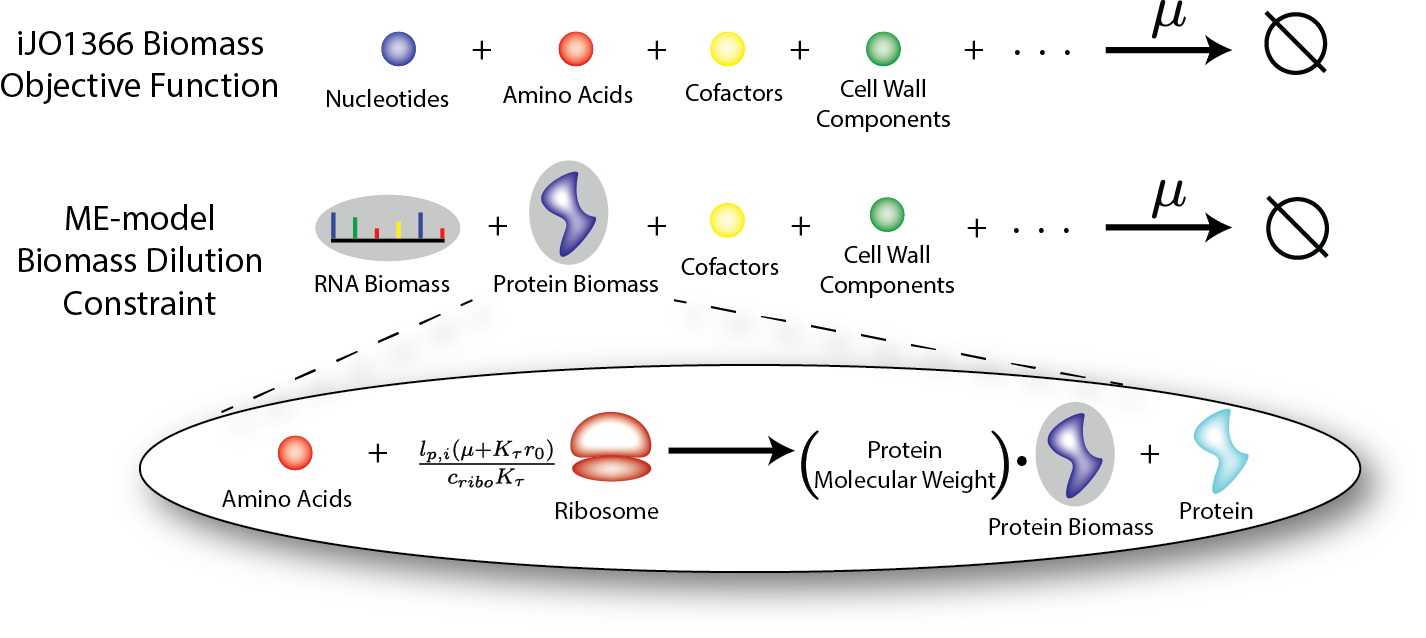

Supplement: S1 File — The COBRAme version 0.0.9 source code. The latest version of COBRAme can be downloaded from https://github.com/SBRG/cobrame. (ZIP) [file pcbi.1006302.s001.zip › S1_File/docs/_static/Biomass_constraint_figure.png]

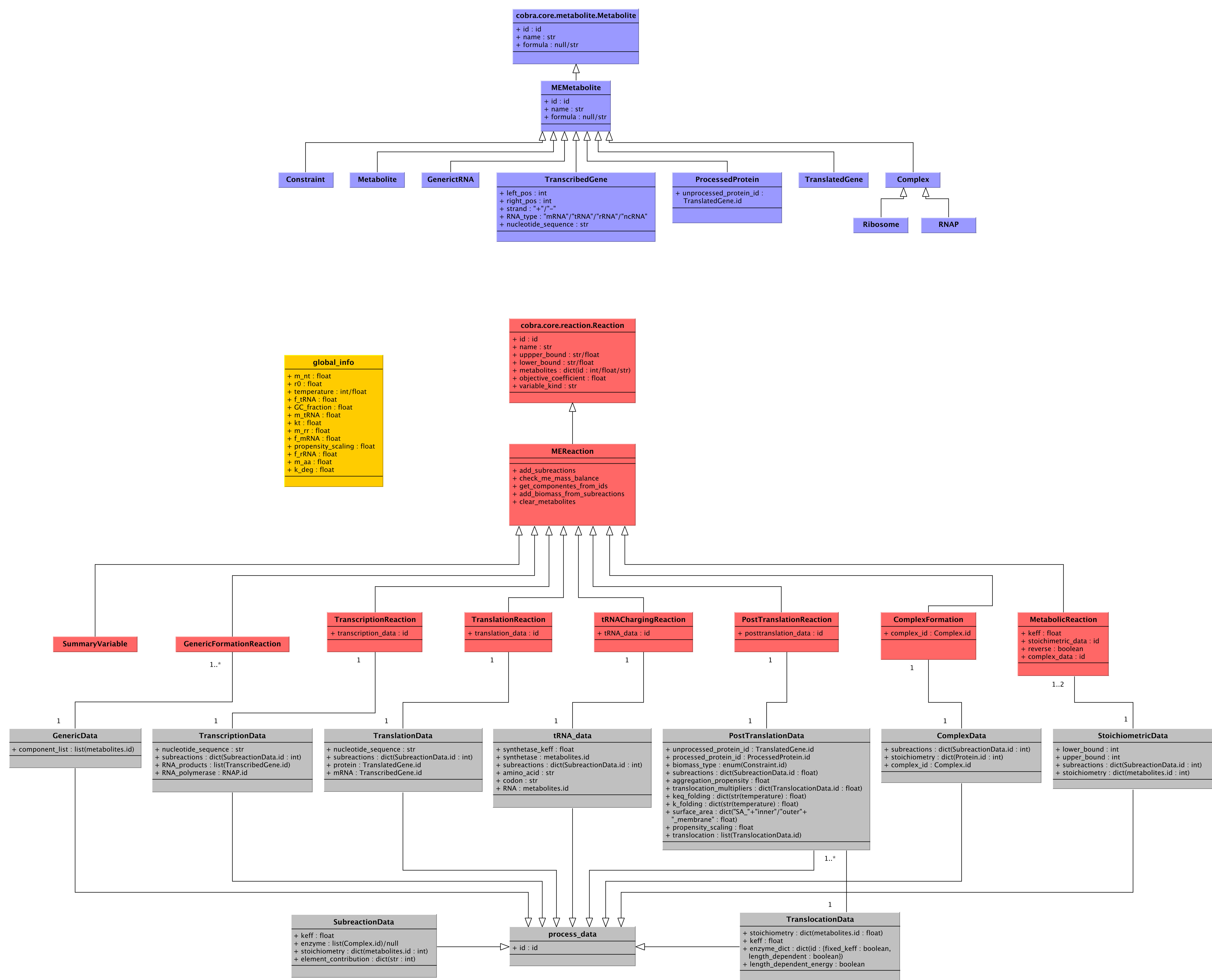

Supplement: S1 File — The COBRAme version 0.0.9 source code. The latest version of COBRAme can be downloaded from https://github.com/SBRG/cobrame. (ZIP) [file pcbi.1006302.s001.zip › S1_File/docs/_static/cobrame_full_uml.pdf]

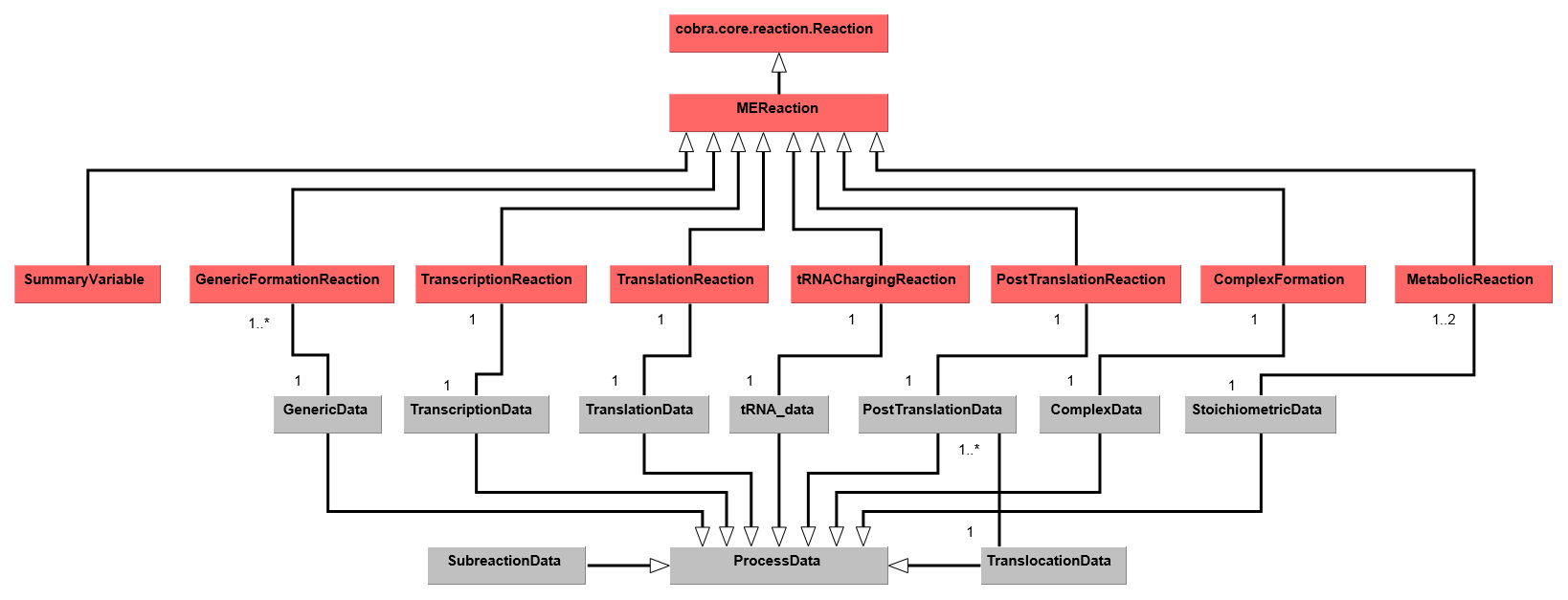

Supplement: S1 File — The COBRAme version 0.0.9 source code. The latest version of COBRAme can be downloaded from https://github.com/SBRG/cobrame. (ZIP) [file pcbi.1006302.s001.zip › S1_File/docs/_static/cobrame_uml.png]

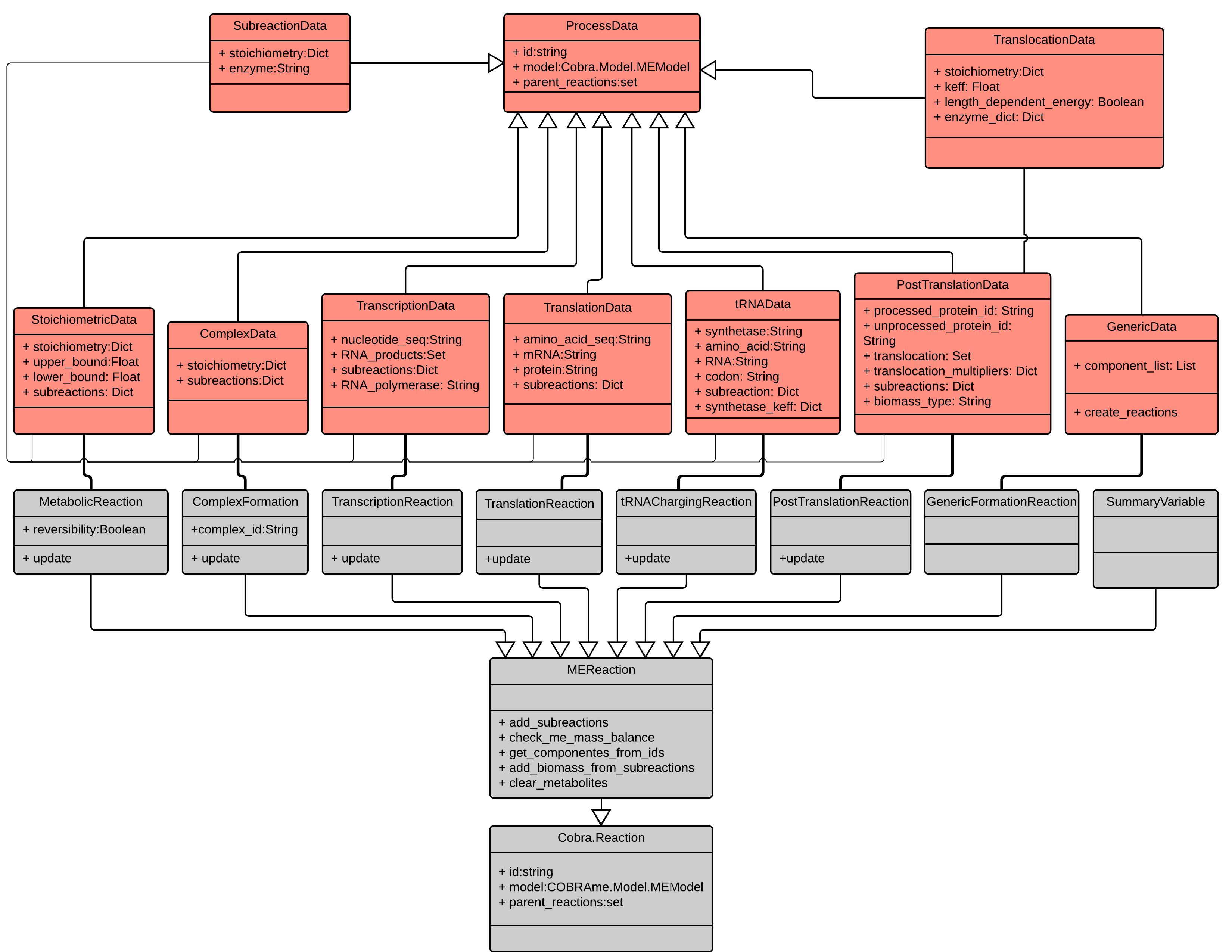

Supplement: S1 File — The COBRAme version 0.0.9 source code. The latest version of COBRAme can be downloaded from https://github.com/SBRG/cobrame. (ZIP) [file pcbi.1006302.s001.zip › S1_File/docs/_static/ME_UML.png]

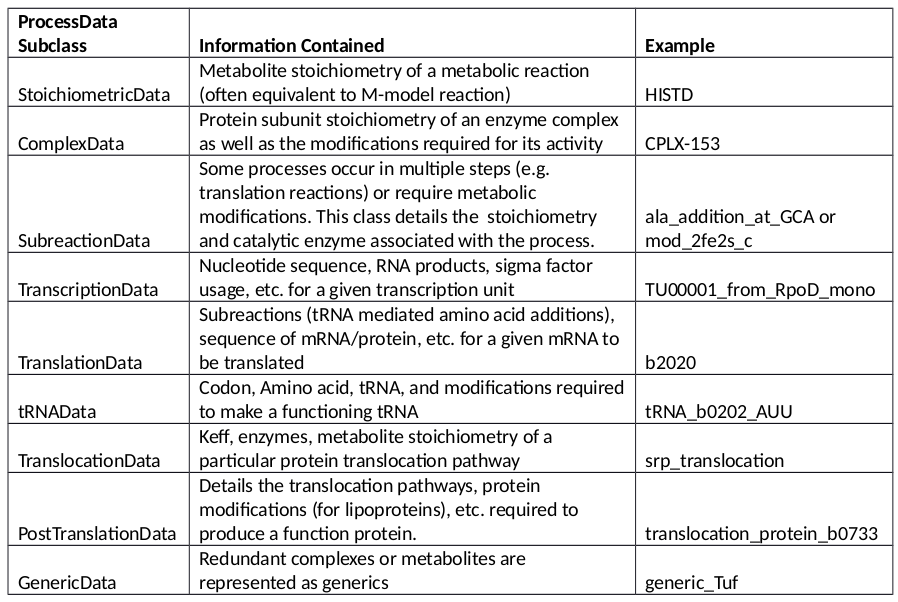

Supplement: S1 File — The COBRAme version 0.0.9 source code. The latest version of COBRAme can be downloaded from https://github.com/SBRG/cobrame. (ZIP) [file pcbi.1006302.s001.zip › S1_File/docs/_static/process_data_definitions.png]

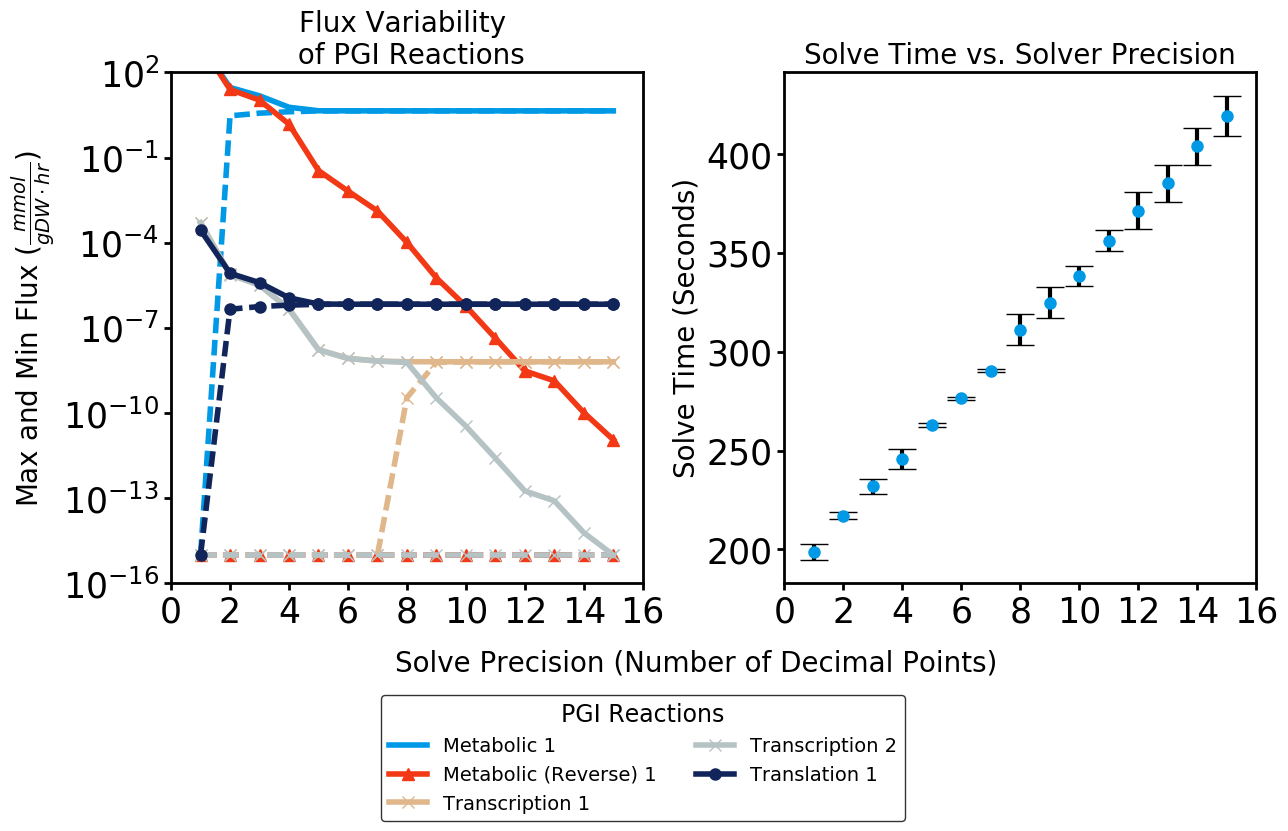

Supplement: S3 File — All results outlined in the manuscript can be reproduced by following the instructions in the README. A JSON version of iJL1678b-ME is also contained in this file. Alternatively, these scripts can be found at https://github.com/coltonlloyd/cobrame_supplement. (ZIP) [file pcbi.1006302.s003.zip › S3_File/Figure_4_solver_accuracy/Figure_4_PGI.png]

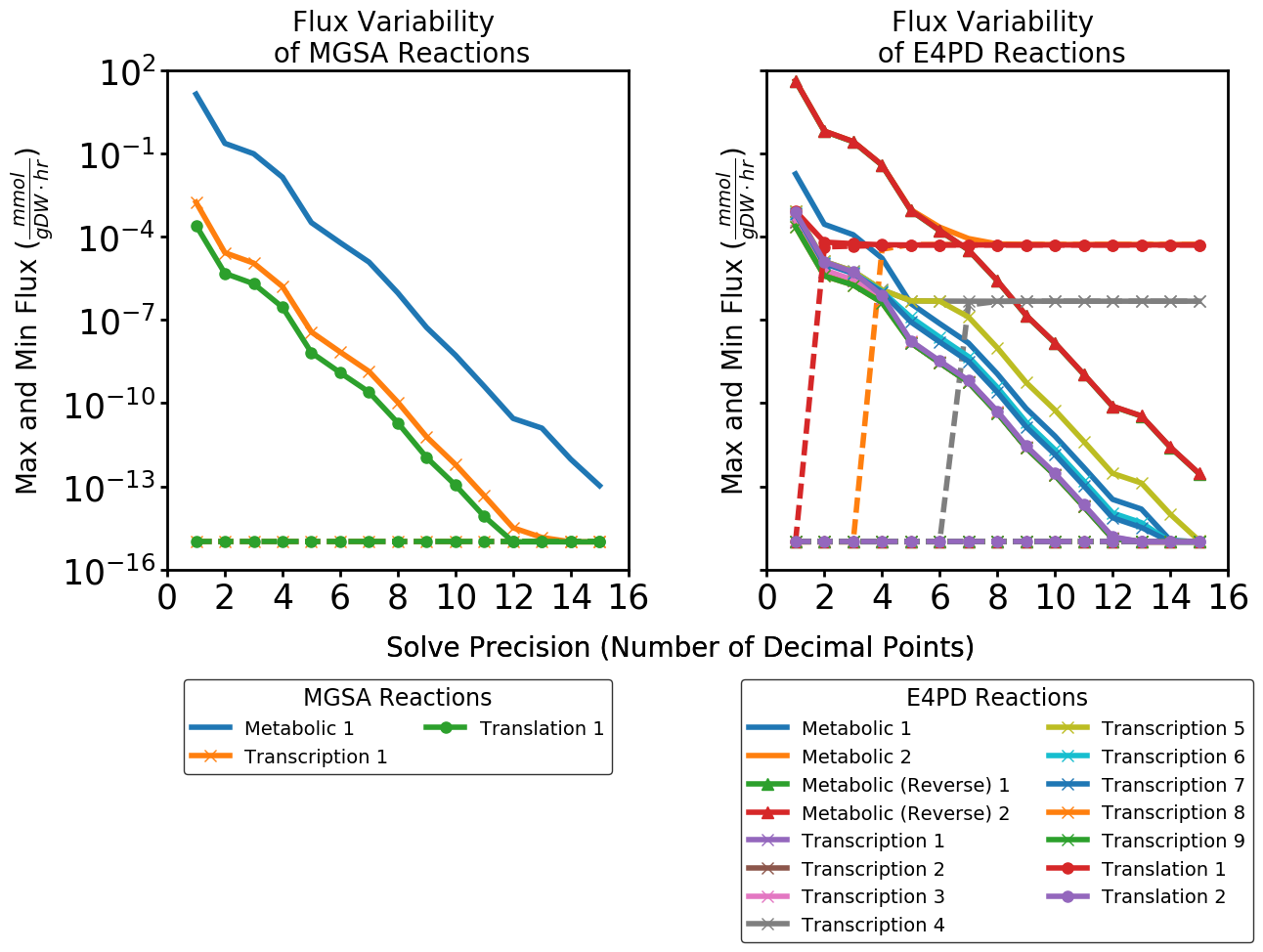

Supplement: S3 File — All results outlined in the manuscript can be reproduced by following the instructions in the README. A JSON version of iJL1678b-ME is also contained in this file. Alternatively, these scripts can be found at https://github.com/coltonlloyd/cobrame_supplement. (ZIP) [file pcbi.1006302.s003.zip › S3_File/Figure_4_solver_accuracy/Figure_S2.png]

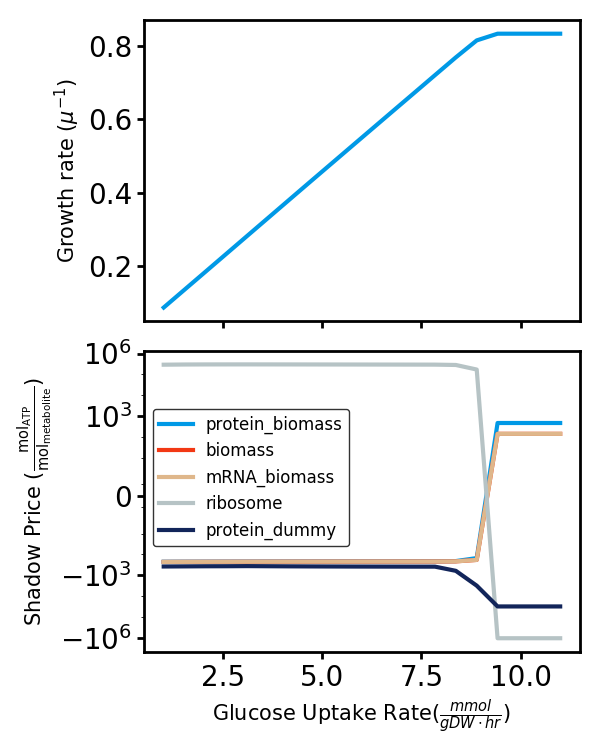

Supplement: S3 File — All results outlined in the manuscript can be reproduced by following the instructions in the README. A JSON version of iJL1678b-ME is also contained in this file. Alternatively, these scripts can be found at https://github.com/coltonlloyd/cobrame_supplement. (ZIP) [file pcbi.1006302.s003.zip › S3_File/Figure_S1_shadow_prices/Figure_S1.png]
