# Supplementary material for: COBRAme: A computational framework for genome-scale models of metabolism and gene expression
Source: PLoS Comput Biol. 2018 Jul 5;14(7):e1006302. doi: 10.1371/journal.pcbi.1006302 (PMC6049947; doi:10.1371/journal.pcbi.1006302)
Supplement: S4 File — The latest version of the documentation can be found at https://cobrame.readthedocs.io. (PDF) [file pcbi.1006302.s004.pdf]

COBRAme Documentation

Release 0.0.9

Colton Lloyd, Ali Ebrahim, Laurence Yang

Jun 06, 2018

Contents

1 ME-model Fundamentals **2**
- 1.1 Coupling Constraints . . . 2
- 1.2 Biomass Dilution Constraints . . . 4

2 COBRAme Software Architecture **6**
- 2.1 ProcessData . . . 6
- 2.2 MEReaction . . . 7
- 2.3 Overview . . . 7

3 Building a ME-model **9**
- 3.1 Overview . . . 9
- 3.2 Initializing new ME-Models . . . 10
- 3.3 Adding Reactions without utility functions . . . 11
- 3.4 Adding Reactions using utility functions . . . 25

4 Reaction Properties **29**
- 4.1 Metabolic Reactions . . . 29
- 4.2 Transcription Reactions . . . 32
- 4.3 Translation Reactions . . . 34
- 4.4 ComplexFormation Reactions . . . 35

5 ME-model Saving and Loading **37**
- 5.1 As a full JSON file . . . 37
- 5.2 As a reduced JSON file . . . 37
- 5.3 As a pickle file . . . 38

6 Coupling Constraint Derivations **39**
- 6.1 Parameters . . . 39
- 6.2 Derivation of mRNA coupling coefficients . . . 40
- 6.3 Derivation of ribosome coupling coefficients . . . 43

7 cobrame package **45**
- 7.1 Subpackages . . . 45
- 7.2 Module contents . . . 69

8 Indices and tables **70**

Python Module Index **71**

This resource is intended to:

1. Provide an overview of what ME-models are and how they work
2. Describe the architecture of the COBRAme code base.
3. Highlight the major object classes and how they interrelate
4. Demonstrate basic model building and editing procedures
5. Provide examples of how to query, edit and update information in a constructed ME-model

CHAPTER 1

ME-model Fundamentals

Models of metabolism and expression (ME-models) are unique in that they are capable of predicting the optimal macromolecular expression required to sustain a metabolic phenotype. In other words, they are capable of making novel predictions of the amount of individual protein, nucleotides, cofactors, etc. that the cell must synthesize in order to grow optimally. To enable these types of predictions, ME-models differ from metabolic models (M-models) in a few key ways:

1. ME-models are multi-scale in nature so they require the addition of *coupling constraints* to couple cellular processes to each other.
2. ME-models predict the biomass composition of a growing cell thus forgoing much of the M-model biomass composition function. For this reason, the function representing growth needs to be updated.

These two ME-model features are briefly described below. Their practical implementation is further outlined in **Building a ME-model**.

1.1 Coupling Constraints

Coupling constraints are required in an ME-model in order to couple a reaction flux to the synthesis of the macromolecule catalyzing the flux. The easiest example of this is for the coupling of metabolic enzymes to metabolic reactions. This has the form:

$$\frac{\mu}{k_{eff}}$$

where μ is the growth rate and k_{eff} is an approximation of the effective turnover rate for the metabolic process. The coupling of enzyme synthesis cost to metabolic flux scales with μ to represent the dilution of macromolecules as they are passed on the daughter cells. More macromolecules are therefore diluted at faster growth rates. Enzyme turnover rates determine the efficiency of of an enzyme *in vivo* and are largly unknown for a majority of metabolic and expression-related enzymes. Optimizing the vector of $k_{eff}s$ for the cellular processes modeled in the ME-model is an ongoing area of research.

Currently for the *E. coli* ME-model, the $k_{eff}s$ are set with an average of 65 s^{-1} and scaled by their solvent accessible surface area (approximated as $protein\_moleculare\_weight^{\frac{3}{4}}$). A set ~125 metabolic $k_{eff}s$ were found by

Ebrahim et. al. 2016 to be particularly important in *E. coli* for computing an accurate metabolic/proteomic state using proteomics data. We suspect similar observations would be seen in other organisms.

For non metabolic macromolecules such as ribosome, mRNA, tRNA and RNA polymerase, the coupling constrains coefficients ('$k_{eff}s$') are derived by essentially back-calculating the individual rates using a measured RNA-to-Protein ratio from Scott et. al. 2010 and measured mRNA, tRNA and rRNA fractions. The coupling constraints coefficients for these macromolecules are derived in detail in O'Brien et. al. 2013. Applying these constraints results in a final nonlinear optimization problem (NLP) shown below. COBRAme reformulates these coupling constraints to embed them directly into the reaction which they are used as follows:

$$
\begin{aligned}
&\max_{v,\mu}\ \mu \\
&\ \text{s.t. } Sv = 0 \\
&\quad v_{\text{formation,Ribosome}} - \sum_{i\in Peptide}\left(\frac{l_{p,i}}{c_{\text{ribo}}\kappa_\tau}(\mu + r_0\kappa_\tau)\cdot v_{\text{translation},i}\right) = 0 \\
&\quad v_{\text{formation,RNAP}} - \sum_{i\in TU}\left(\frac{l_{\text{TU},i}}{3c_{\text{ribo}}\kappa_\tau}(\mu + r_0\kappa_\tau)\cdot v_{\text{transcription},i}\right) = 0 \\
&\quad v_{\text{formation},j} \\
&\qquad - \sum_{i\in \text{generic\_tRNA}_{AA}}\left(\left(1+\frac{\mu}{k_{eff,tRNA}}\right)\frac{\mu}{k_{eff,charging}}v_{\text{charging},i}\right) = 0, \\
&\qquad\quad \forall j \in Synthetase \\
&\quad v_{\text{formation},j} - \sum_{i\in \text{enzymatic reaction}}\left(\frac{\mu}{k^{\text{eff}}_{ij}}v_{\text{usage},i}\right) = 0, \quad \forall j \in Enzyme \\
&\quad v_{\text{formation},j} - \sum_{i\in \text{tRNA anticodons}}\frac{(\mu+\kappa_\tau r_0)}{\kappa_\tau c_{tRNA,j}}v_{charging,i} = 0, \quad \forall j \in tRNA \\
&\quad v_{\text{degradation},j} - \frac{k_{deg,j}}{3\kappa_\tau c_{mRNA}}\cdot\frac{\mu+\kappa_\tau r_0}{\mu}v_{translation,j} = 0, \quad \forall j \in mRNA \\
&\quad v_{\text{formation},j} - \frac{(\mu+\kappa_\tau r_0)}{3\kappa_\tau c_{mRNA}}v_{translation,j} = 0, \quad \forall j \in mRNA \\
&\quad v^L \le v \le v^U \\
&\quad \mu \le v_{biomass\_dilution} \le \mu
\end{aligned}
$$

The biomass_dilution constraint is discussed below.

1.1.1 Previous ME-model Coupling Constraint Implementation

The previous iterations of ME-models applied coupling constraints using three separate reactions. An example for a generic "enzymatic reaction" could be represented as follows:

$$\textbf{Enzyme Priming: } \text{enzyme} \xrightarrow{v0} \text{enzyme\_primed} + \alpha\cdot\text{coupling}$$

$$\textbf{Enzymatic Reaction: } \text{a} + \text{enzyme\_primed} \xrightarrow{v1} \text{enzyme + b}$$

$$\textbf{Dilution Coupling Reaction: } \text{enzyme + coupling} \xrightarrow{v2} \emptyset$$

Where α is the coupling coefficient applied to "coupling", a metabolite (constraint) which effectively determines the minimal rate that the third dilution coupling reaction ($v2$) must proceed. For previous ME-model implementations, this coupling constraint was given a "_constraint_sense" in COBRApy of 'L' (less than or equal to) meaning that for this toy example $v0 = v1$ and $v2 \ge \alpha \cdot v1$.

1.1.2 COBRAme Coupling Constraint Implementation

With COBRAme it is assumed that the optimal ME solution will never dilute more enzyme than is required by the coupling constraint thus constraining $v2 = \alpha \cdot v1$ and allowing us to combine the implementation of the constraint with the reaction that uses the enzyme to give.

$$\text{a} + \alpha \cdot \text{enzyme} \xrightarrow{v1} \text{b}$$

The coupling constraints and coefficients were derived as in O'Brien et. al. 2013. As stated above, however, these were implemented in the current study as equality constraints . Effectively, this means that each ME-model solution will give the computed optimal proteome allocation for the *in silico* conditions. Previous ME-model formulations have applied the constraints as inequalities thus allowing the simulation to overproduce macromolecule components. While overproduction is seen *in vivo* in cells, this phenomenon would not be selected as the optimal solution. Furthermore, using inequality constraints greatly expands the size of the possible solution space significantly increasing the time required to solve the optimization. Reformulating the model using equality constraints thus resulted in a reduced ME-matrix with the coupling constraints embedded directly into the reaction in which they are used.

1.2 Biomass Dilution Constraints

For metabolic models (M-models), the biomass objective function has been used to represent the amount of biomass production that is required for the cell to double at a specified rate. The metabolites represented in the biomass function are typically building blocks for major macromolecules (e.g. amino acids and nucleotides), cell wall components and cofactors. The coefficients of the biomass objective function are determined from empirical measurements from a cell growing at a measured rate. Since ME-models explicitly compute the predicted amount of RNA, protein, cofactors, etc. necessary for growth, this concept has to be modified for ME-models.

This is accomplished via the *biomass_dilution* variable (reaction), which contains a *biomass* constraint (pseudo metabolite) that represents the mass produced by the synthesis of each functional RNA or protein. This reaction essentially ensured that the ME-model can only produce biomass at the rate it is being diluted (via growth and division).

1.2.1 Implementation

For each transcription or translation reaction in an ME-model an amount of a *biomass* constraint (pseudo metabolite) is created with a stoichiometry equal to the molecular weight of the mRNA or protein being made (in *kDA*). The below figure shows an example of this where a translation reaction produces both the catalytic protein as well as the protein biomass constraint. The formed *protein_biomass* constraint is a participant in the overall ME-model *Biomas_ Dilution* reaction which restricts the total production of the major biomass components to equal the rate at which biomass is diluted (i.e. the cell's growth rate, μ).

Some biomass constituents do not have a mechanistic function in the ME-model (e.g. cell wall components, DNA and glycogen). These metabolites are included in the *biomass_dilution* reaction indentical to the M-model biomass reaction .

1.2.2 Unit Check

The units for this contraint work out as follows:

The units of a given reaction in the ME-model are in molecules per hour.

$$v_i \Rightarrow \frac{mmol}{gDW \cdot hr}$$

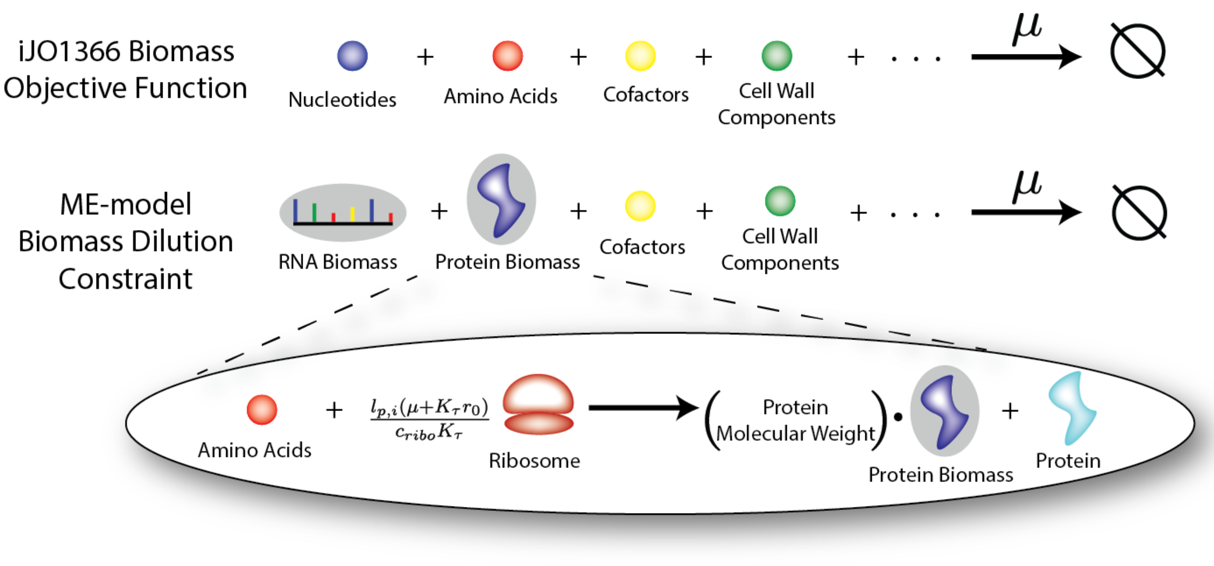

Fig. 1.1: Biomass Dilution for iJO1366 and for a ME-model

The individual components of the biomass dilution constraints are in units of kDa.

$$molecular\_weight \Rightarrow kDA[\frac{g}{mmol}]$$

Therefore, when the *biomass dilution* variable (reaction) carries flux it gives units of hr^{-1} representing the growth rateof the cell, μ

$$molecular\_weight \cdot v_i \Rightarrow hr^{-1}[\mu]$$

CHAPTER 2

COBRAme Software Architecture

The COBRAme codebase is constructed with the intention of including all of the metabolic processes and complexity associated with gene expression, while still giving a final ME-model that is not prohibitively difficult to use and interpret. To accomplish this COBRAme separates the information associated with each cellular process from the actual ME-model reaction in which the process is modeled. We call these two major python classes ProcessData and MEReaction, respectively. The logic behind each of these classes is briefly presented below, and a description of the class attributes and properties is presented here in the form of a `UML Diagram`.

2.1 ProcessData

The previous ME-models for *E. coli* and *T. Maritima* were reconstructed by first establishing a database containing information about all gene expression processes known in the organism being model. This included, for instance, enzyme complex subunit stoichiometries or the *E. coli* transcription unit architecture. Then, when building the ME-model, the database was queried to obtain any relevant information and incorporate this into the appropriate reactions. For the COBRAme formulation, this database was replaced by the ProcessData "information storage" class. The ProcessData class generally consists of attributes which use simple python types (string, dictionary, etc.) to describe features of a biological process.

The use of the ProcessData class has the advantage of:

1. Simplifying the process of querying information associated with a cellular function
2. Allowing edits to this information which can easily be applied throughout the model without rebuilding from scratch
3. Enabling additional computations to be performed and seamlessly accessed as ProcessData class methods

The ProcessData classes are broken into the following subclasses:

ProcessData Subclass	Information Contained	Example
StoichiometricData	Metabolite stoichiometry of a metabolic reaction (often equivalent to M-model reaction)	HISTD
ComplexData	Protein subunit stoichiometry of an enzyme complex as well as the modifications required for its activity	CPLX-153
SubreactionData	Some processes occur in multiple steps (e.g. translation reactions) or require metabolic modifications. This class details the stoichiometry and catalytic enzyme associated with the process.	ala_addition_at_GCA or mod_2fe2s_c
TranscriptionData	Nucleotide sequence, RNA products, sigma factor usage, etc. for a given transcription unit	TU00001_from_RpoD_mono
TranslationData	Subreactions (tRNA mediated amino acid additions), sequence of mRNA/protein, etc. for a given mRNA to be translated	b2020
tRNAData	Codon, Amino acid, tRNA, and modifications required to make a functioning tRNA	tRNA_b0202_AUU
TranslocationData	Keff, enzymes, metabolite stoichiometry of a particular protein translocation pathway	srp_translocation
PostTranslationData	Details the translocation pathways, protein modifications (for lipoproteins), etc. required to produce a function protein.	translocation_protein_b0733
GenericData	Redundant complexes or metabolites are represented as generics	generic_Tuf

2.2 MEReaction

COBRAme compartmentalizes the major reaction types into their own MEReaction classes. Each of these classes contains a single *update* function which effectively reads in the appropriate ProcessData types, applies the coupling constraints on the macromolecules, and assembles these components into a complete model reaction. This allows changes made to the ProcessData describing a particular cellular process to easily be incorporated into the reactions which it was used.

2.3 Overview

Using the major classes described above, reconstructing a ME-model can then be broken down into three steps:

1. Define and construct all necessary ProcessData objects
2. Link the ProcessData to the appropriate MEReaction instance
3. Update all MEReactions to incorporate ProcessData information into functional reactions

The overall codebase architecture is displayed below in a UML diagram. This reduced UML highlights the ways which MEReactions and ProcessData are linked in a ME-model reconstructed using COBRAme. A full UML representation of the COBRAme/ME-model software architecture can be downloaded `here`

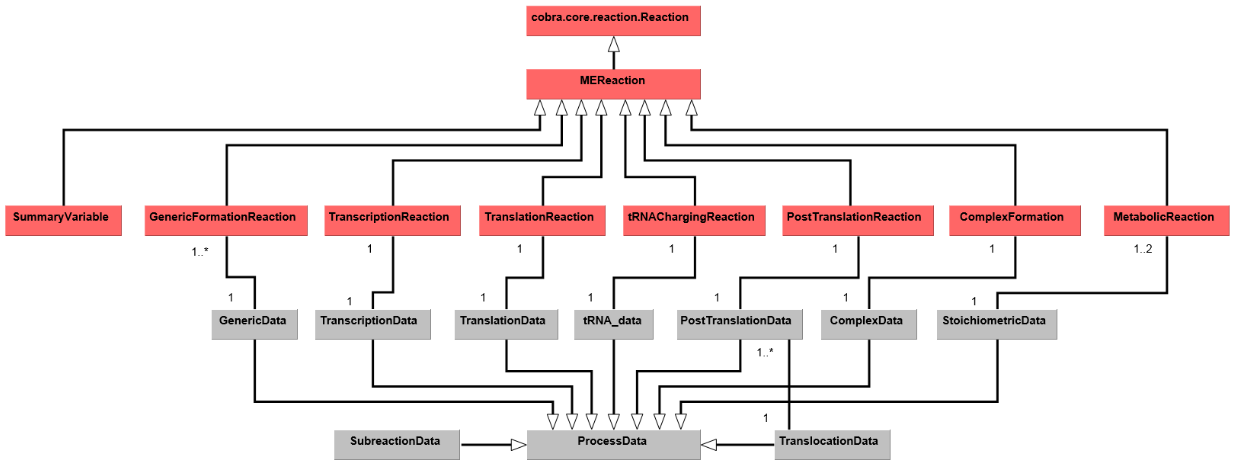
cobra.core.reaction.Reaction
MEReaction
SummaryVariable
GenericFormationReaction
TranscriptionReaction
TranslationReaction
tRNAChargingReaction
PostTranslationReaction
ComplexFormation
MetabolicReaction
1..*
1
1
1
1
1
1..2
1
1
1
1
1
1
1
GenericData
TranscriptionData
TranslationData
tRNA_data
PostTranslationData
ComplexData
StoichiometricData
1..*
1
SubreactionData
ProcessData
TranslocationData

CHAPTER 3

Building a ME-model

```
In [1]: from __future__ import print_function

        import cobrame
        from cobrame.util import dogma, building
        import cobrame.util.building
        import cobra
        import cobra.test
        from collections import defaultdict

        #import warnings
        #warnings.filterwarnings('ignore')
```

```
/home/sbrg-cjlloyd/cobrapy/cobra/io/sbml3.py:24: UserWarning: Install lxml for faster SBML I/O
  warn("Install lxml for faster SBML I/O")
/home/sbrg-cjlloyd/cobrapy/cobra/io/__init__.py:12: UserWarning: cobra.io.sbml requires libsbml
  warn("cobra.io.sbml requires libsbml")
```

3.1 Overview

COBRAme is constructed entirely over COBRApy. This means that ME-model reactions will have all of the same properties, methods, and functions as a COBRApy reaction. However, one key difference between M and ME models is that many reactions involved in gene expression are effecively templates that are constructed identically but vary based on characteristics of the gene being expressed. For example, a gene with a given nucleotide sequence is always translated following the same rules provided by the codon table for that organism.

In order to facilliate the template nature of many gene expression reactions, COBRAme reactions are constructed and their components are manipulated through the use of `ProcessData` classes. These act as information vessels for holding the information assocatied with a cellular process in simple, standard datatypes such as dictionaries and strings.

This tutorial will go step-by-step through the process of creating a generic enzyme catalyzed reaction (i.e. metabolic

reaction):

$$a \to b$$

which requires the formation and coupling of **complex_ab** in order to proceed.

In order for this reaction to carry flux in the model we will additionally need to first add the corresponding:

1. **Transcription reactions**
2. **Translation reactions**
3. **tRNA charging reactions**
4. **Complex formation reactions**

Once these are added we will add in the synthesis of key macromolecular components (ribosome, RNA polymerase, etc.) and show how they are coupled to their respective reactions. The derived coupling coefficients will also be described. For more on the derivation of the coupling coefficients, reference the supplemental text of O'brien et. al. 2013

3.2 Initializing new ME-Models

When applying some constraints in the ME-model, metabolite properties are required. For instance, to calculate the total biomass (by molecular weight) produced by a particular macromolecule, the amino acid, nucleotide, etc. molecular weights are required. To enable these calculations, all metabolites from *i*JO1366, along with their metabolite attributes are added to the newly initialized ME-model.

Further the reactions from *i*JO1366 will be added to the ME-model to demonstrate ME-model solving procedures.

```
In [2]: # create empty ME-model
        me = cobrame.MEModel('test')
        ijo = cobra.test.create_test_model('ecoli')
```

```
In [3]: # Add all metabolites and reactions from iJO1366 to the new ME-model
        for met in ijo.metabolites:
            me.add_metabolites(met)
        for rxn in ijo.reactions:
            me.add_reaction(rxn)
```

The ME-model contains a "global_info" attribute which stores information used to calculate coupling constraints, along with other functions. The specifics of each of these constraints will be discussed when they are implemented.

Note: k_deg will initially be set to 0. We will apply RNA degradation later in the tutorial.

```
In [4]: # "Translational capacity" of organism
        me.global_info['kt'] = 4.5  # (in h-1)scott 2010, RNA-to-protein curve fit
        me.global_info['r0'] = 0.087  # scott 2010, RNA-to-protein curve fit
        me.global_info['k_deg'] = 1.0/5. * 60.0  # 1/5 1/min 60 min/h # h-1

        # Molecular mass of RNA component of ribosome
        me.global_info['m_rr'] = 1453. # in kDa

        # Average molecular mass of an amino acid
        me.global_info['m_aa'] = 109. / 1000.  # in kDa
```

```
# Proportion of RNA that is rRNA
me.global_info['f_rRNA'] = .86
me.global_info['m_nt'] = 324. / 1000.  # in kDa
me.global_info['f_mRNA'] = .02

# tRNA associated global information
me.global_info['m_tRNA'] = 25000. / 1000.  # in kDA
me.global_info['f_tRNA'] = .12

# Define the types of biomass that will be synthesized in the model
me.add_biomass_constraints_to_model(["protein_biomass", "mRNA_biomass", "tRNA_biomass", "rRNA
                                     "ncRNA_biomass", "DNA_biomass", "lipid_biomass", "const
                                     "prosthetic_group_biomass", "peptidoglycan_biomass"])
```

Define sequence of gene that will be expressed in tutorial

```
In [5]: sequence = ("ATG" + "TTT" * 12 + "TAT" * 12 +
                    "ACG" * 12 + "GAT" * 12 + "AGT" * 12 + "TGA")
```

3.3 Adding Reactions without utility functions

We'll first demonstrate how transcription, translation, tRNA charging, complex formation, and metabolic reactions can be added to a model without using any of the utility functions provided in `cobrame.util.building.py`. The second half of the tutorial will show how these utility functions can be used to add these reactions.

The basic workflow for adding any reaction to a ME-model using COBRAme occurs in three steps:

1. **Create the ProcessData(s) associated with the reaction and populate them with the necessary information**
2. **Create the MEReaction and link the appropriate ProcessData**
3. **Execute the MEReaction's update method**

3.3.1 Add Transcription Reaction

Add TranscribedGene metabolite to model

Transcription reactions is unique in that they occur at a transcription unit level and can code for multiple transcript products. Therefore the nucleotide sequence of both the transcription unit and the RNA transcripts must be defined in order to correctly construct a transcription reaction.

class `cobrame.core.component.`**`TranscribedGene`**(*id*, *rna_type*, *nucleotide_sequence*)

Metabolite class for gene created from *`cobrame.core.reaction.TranscriptionReaction`*

Parameters

- **`id`** (*`str`*) – Identifier of the transcribed gene. As a best practice, this ID should be prefixed with 'RNA + _'
- **`RNA_type`** (*`str`*) – Type of RNA encoded by gene sequence (mRNA, rRNA, tRNA, or ncRNA)
- **`nucleotide_sequence`** (*`str`*) – String of base pair abbreviations for nucleotides contained in the gene

`left_pos`

int – Left position of gene on the sequence of the (+) strain

right_pos
int – Right position of gene on the sequence of the (+) strain

strand
str –

- (+) if the RNA product is on the leading strand
- (-) if the RNA product is on the comple(mentary strand

```
In [6]: gene = cobrame.TranscribedGene('RNA_a', 'mRNA', sequence)
        me.add_metabolites([gene])
```

When adding the `TranscribedGene` above, the `RNA_type` and `nucleotide_sequence` was assigned to the gene. This sequence cannot be determined from the transcription unit (TU) sequence because a single TU often contains several different RNAs.

Add TranscriptionData to model

class `cobrame.core.processdata.`**`TranscriptionData`**(*id*, *model*, *rna_products=set([])*)
Class for storing information needed to define a transcription reaction

Parameters

- **id** (*str*) – Identifier of the transcription unit, typically beginning with 'TU'
- **model** (*cobrame.core.model.MEModel*) – ME-model that the TranscriptionData is associated with

nucleotide_sequence
str – String of base pair abbreviations for nucleotides contained in the transcription unit

RNA_products
set – IDs of *cobrame.core.component.TranscribedGene* that the transcription unit encodes. Each member should be prefixed with "RNA + _"

RNA_polymerase
str – ID of the *cobrame.core.component.RNAP* that transcribes the transcription unit. Different IDs are used for different sigma factors

subreactions
`collections.DefaultDict(int)` – Dictionary of {*cobrame.core.processdata.SubreactionData* ID: num_usages} required for the transcription unit to be transcribed

```
In [7]: transcription_data = cobrame.TranscriptionData('TU_a',me,rna_products={'RNA_a'})
        transcription_data.nucleotide_sequence = sequence
```

Add TranscriptionReaction to model

And point `TranscriptionReaction` to `TranscriptionData`

class `cobrame.core.reaction.`**`TranscriptionReaction`**(*id*)
Transcription of a TU to produced TranscribedGene.

RNA is transcribed on a transcription unit (TU) level. This type of reaction produces all of the RNAs contained within a TU, as well as accounts for the splicing/excision of RNA between tRNAs and rRNAs. The appropriate RNA_biomass constrain is produced based on the molecular weight of the RNAs being transcribed

Parameters **id** (*str*) – Identifier of the transcription reaction. As a best practice, this ID should be prefixed with 'transcription + _'

```
In [8]: transcription_rxn = cobrame.TranscriptionReaction('transcription_TU_a')
        transcription_rxn.transcription_data = transcription_data
        me.add_reactions([transcription_rxn])
```

Update TranscriptionReaction

`TranscriptionReaction.`**`update`**(*verbose=True*)

Creates reaction using the associated transcription data and adds chemical formula to RNA products

This function adds the following components to the reaction stoichiometry (using 'data' as shorthand for *cobrame.core.processdata.TranscriptionData*):

1. RNA_polymerase from data.RNA_polymerase w/ coupling coefficient (if present)
2. RNA products defined in data.RNA_products
3. Nucleotide reactants defined in data.nucleotide_counts
4. If tRNA or rRNA contained in data.RNA_types, excised base products
5. Metabolites + enzymes w/ coupling coefficients defined in data.subreactions (if present)
6. Biomass *cobrame.core.component.Constraint* corresponding to data.RNA_products and their associated masses
7. Demand reactions for each transcript product of this reaction

Parameters **verbose** (*bool*) – Prints when new metabolites are added to the model when executing update()

```
In [9]: transcription_rxn.update()
        print(transcription_rxn.reaction)
```

```
86 atp_c + 38 ctp_c + 12 gtp_c + 50 utp_c --> RNA_a + 59.172286 mRNA_biomass + 186 ppi_c
```

```
/home/sbrg-cjlloyd/cobrame/cobrame/core/reaction.py:813 UserWarning: RNA Polymerase () not found
```

Note: the RNA_polymerase complex is not included in the reaction. This will be added later

This reaction now produces a small amount of the a `mRNA_biomass` metabolite (constraint). This term has a coefficient corresponding to the molecular weight (in kDA) of the RNA being transcribed. This constraint will be implemented into a $v_{biomasss\_dilution}$ reaction with the form:

$$\mu \leq v_{biomass\_dilution} \leq \mu$$

A mathematical description of the `biomass` constraint can be found in *Biomass Dilution Constraints* in **ME-Model Fundamentals**.

Note: This is not a complete picture of transcription because the RNA polymerase is missing.

Incorporate RNA Polymerase

For the purposes of this tutorial, we'll skip the steps required to add the reactions to form the RNA_polymerase. The steps however are identical to those outlined in **add enzyme complexes** below

class `cobrame.core.component.`**`RNAP`**(*id*)

Metabolite class for RNA polymerase complexes. Inherits from *cobrame.core.component.Complex*

Parameters **id** (*str*) – Identifier of the RNA Polymerase.

```
In [10]: RNAP = cobrame.RNAP('RNA_polymerase')
         me.add_metabolites(RNAP)
```

Associate RNA_polymerase with all `TranscriptionData` and update

```
In [11]: for data in me.transcription_data:
             data.RNA_polymerase = RNAP.id
         me.reactions.transcription_TU_a.update()

         print(me.reactions.transcription_TU_a.reaction)
```

```
0.000888870536605567*mu + 0.000347992814865795 RNA_polymerase + 86 atp_c + 38 ctp_c + 12 gtp_c + 50 ut
```

The coefficient for RNA_polymerase is the first instance in this tutorial where a coupling constraint is imposed. In this case the constraint couples the formation of a RNA_polymerase metabolite to its transcription flux. This constraint is formulated as in O'brien et. al. 2013, with assumption that $k_{rnap} = 3 \cdot k_{ribosome}$ based on data from Proshkin et al. 2010:

$$v_{dilution,RNAP,j} = \frac{l_{TU,j}}{3 c_{ribo} \kappa_\tau} v_{transcription,j} (\mu + r_0 \kappa_\tau), \forall j \in TU \tag{3.1}$$

where:

- κ_τ and r_0 are phenomenological parameters from Scott et. al. 2010 that describe the linear relationship between the observed RNA/protein ratio of *E. coli* and its growth rate (μ)
- $c_{ribo} = \frac{m_{rr}}{f_{rRNA} \cdot m_{aa}}$ where: m_{rr} is the mass of rRNA per ribosome. f_{rRNA} is the fraction of total RNA that is rRNA m_{aa} is the molecular weight of an average amino acid
- $v_{transcription,j}$ is the rate of transcription for TU_j
- $l_{TU,j}$ is number of nucleotides in TU_j

3.3.2 Add Translation Reaction

Add TranslationData to model

In order to add a TranslationData object to a ME-model the user must additionally specify the mRNA id and protein id of the translation reaction that will be added. This information as well as a nucleotide sequence is the only information required to add a translation reaction.

class `cobrame.core.processdata.`**`TranslationData`**(*id*, *model*, *mrna*, *protein*)

Class for storing information about a translation reaction.

Parameters

- **id** (*str*) – Identifier of the gene being translated, typically the locus tag
- **model** (*cobrame.core.model.MEModel*) – ME-model that the TranslationData is associated with
- **mrna** (*str*) – ID of the mRNA that is being translated
- **protein** (*str*) – ID of the protein product.

mRNA

str – ID of the mRNA that is being translated

protein
str – ID of the protein product.

subreactions
collections.DefaultDict(int) – Dictionary of {cobrame.core.processdata.SubreactionData.id: num_usages} required for the mRNA to be translated

nucleotide_sequence
str – String of base pair abbreviations for nucleotides contained in the gene being translated

```
In [12]: data = cobrame.TranslationData('a', me, 'RNA_a', 'protein_a')
         data.nucleotide_sequence = sequence
```

Add TranslationReaction to model

By associating the TranslationReaction with its corresponding TranslationData object and running the update function, COBRAme will create a reaction reaction for the nucleotide sequence given based on the organisms codon table and prespecified translation machinery.

class cobrame.core.reaction.**TranslationReaction**(*id*)
Reaction class for the translation of a TranscribedGene to a TranslatedGene

Parameters **id** (*str*) – Identifier of the translation reaction. As a best practice, this ID should be prefixed with 'translation + _'

```
In [13]: rxn = cobrame.TranslationReaction('translation_a')
         rxn.translation_data = data
         me.add_reaction(rxn)
```

Update TranslationReaction

TranslationReaction.**update**(*verbose=True*)
Creates reaction using the associated translation data and adds chemical formula to protein product

This function adds the following components to the reaction stoichiometry (using 'data' as shorthand for *cobrame.core.processdata.TranslationData*):

1. Amino acids defined in data.amino_acid_sequence. Subtracting water to account for condensation reactions during polymerization
2. Ribosome w/ translation coupling coefficient (if present)
3. mRNA defined in data.mRNA w/ translation coupling coefficient
4. mRNA + nucleotides + hydrolysis ATP cost w/ degradation coupling coefficient (if kdeg (defined in model.global_info) > 0)
5. RNA_degradosome w/ degradation coupling coefficient (if present and kdeg > 0)
6. Protein product defined in data.protein
7. Subreactions defined in data.subreactions
8. protein_biomass *cobrame.core.component.Constraint* corresponding to the protein product's mass
9. Subtract mRNA_biomass *cobrame.core.component.Constraint* defined by mRNA degradation coupling coefficinet (if kdeg > 0)

Parameters **verbose** (*bool*) – Prints when new metabolites are added to the model when executing update()

```
In [14]: rxn.update()
         print(rxn.reaction)
```

```
/home/sbrg-cjlloyd/cobrame/cobrame/core/reaction.py:1051 UserWarning: ribosome not found
/home/sbrg-cjlloyd/cobrame/cobrame/core/reaction.py:1094 UserWarning: RNA_degradosome not found
```

```
0.000498399634202103*mu + 0.000195123456790123 + 0.00598079561042524*(mu + 0.3915)/mu RNA_a + 12 asp_
```

In this case the constraint couples the formation of a mRNA metabolite to its translation flux. This constraint is formulated as in O'brien et. al. 2013:

$$v_{dilution,j} = \frac{3}{\kappa_{\tau} c_{mRNA}} \cdot (\mu + \kappa_{\tau} r_0) v_{translation,j}, \qquad \forall j \in mRNA \tag{3.2}$$

where:

- κ_{τ} and r_0 are phenomenological parameters from Scott et. al. 2010 that describe the linear relationship between the observed RNA/protein ratio of *E. coli* and its growth rate (μ)
- $c_{mRNA} = \frac{m_{nt}}{f_{mRNA} \cdot m_{aa}}$ where: m_{nt} is the molecular weight of an average mRNA nucleotide. f_{mRNA} is the fraction of total RNA that is mRNA m_{aa} is the molecular weight of an average amino acid
- $v_{translation,j}$ is the rate of translation for $mRNA_j$

Incorporate Ribosome

class `cobrame.core.component.`**`Ribosome`**(*id*)

Metabolite class for Ribosome complexes. Inherits from *`cobrame.core.component.Complex`*

Parameters **`id`** (*`str`*) – Identifier of the Ribosome.

```
In [15]: ribosome = cobrame.Ribosome('ribosome')
         me.add_metabolites([ribosome])
         me.reactions.translation_a.update()
         print(me.reactions.translation_a.reaction)
```

```
0.000498399634202103*mu + 0.000195123456790123 + 0.00598079561042524*(mu + 0.3915)/mu RNA_a + 12 asp_
```

```
/home/sbrg-cjlloyd/cobrame/cobrame/core/reaction.py:1094 UserWarning: RNA_degradosome not found
```

This imposes a new coupling constraint for the ribosome. In this case the constraint couples the formation of a ribosome to its translation flux. This constraint is formulated as in O'brien et. al. 2013:

$$v_{dilution,ribo,j} = \frac{l_{p,j}}{c_{ribo} \kappa_{\tau}} v_{translation,j} (\mu + r_0 \kappa_{\tau}), \forall j \in mRNA \tag{3.3}$$

where:

- κ_{τ} and r_0 are phenomenological parameters from Scott et. al. 2010 that describe the linear relationship between the observed RNA/protein ratio of *E. coli* and its growth rate (μ)
- $c_{ribo} = \frac{m_{rr}}{f_{rRNA} \cdot m_{aa}}$ where: m_{nt} is the mass of rRNA per ribosome. f_{rRNA} is the fraction of total RNA that is rRNA m_{aa} is the molecular weight of an average amino acid
- $v_{translation,j}$ is the rate of translation for $mRNA_j$
- $l_{p,j}$ is number of amino acids in peptide translated from $mRNA_j$

Note: The above reactions do not provide a complete picture of translation in that it is missing charged tRNAs to facillitate tRNA addition.

Below, we'll correct this by adding in an tRNA charging reaction.

3.3.3 Add tRNA Charging Reaction

Add tRNAData to model

```
In [16]: # Must add tRNA metabolite first
         gene = cobrame.TranscribedGene('RNA_d', 'tRNA', sequence)
         me.add_metabolites([gene])
```

class `cobrame.core.processdata.`**`tRNAData`**(*id*, *model*, *amino_acid*, *rna*, *codon*)
Class for storing information about a tRNA charging reaction.

Parameters

- **`id`** (*`str`*) – Identifier for tRNA charging process. As best practice, this should be follow "tRNA + _ + <tRNA_locus> + _ + <codon>" template. If tRNA initiates translation, <codon> should be replaced with START.
- **`model`** (*`cobrame.core.model.MEModel`*) – ME-model that the tRNAData is associated with
- **`amino_acid`** (*`str`*) – Amino acid that the tRNA transfers to an peptide
- **`rna`** (*`str`*) – ID of the uncharged tRNA metabolite. As a best practice, this ID should be prefixed with 'RNA + _'

`subreactions`
`collections.DefaultDict(int)` – Dictionary of {`cobrame.core.processdata.SubreactionData.id`: num_usages} required for the tRNA to be charged

`synthetase`
str – ID of the tRNA synthetase required to charge the tRNA with an amino acid

`synthetase_keff`
float – Effective turnover rate of the tRNA synthetase

```
In [17]: data = cobrame.tRNAData('tRNA_d_GUA', me, 'val__L_c', 'RNA_d', 'GUA')
```

Add tRNAChargingReaction to model

And point `tRNAChargingReaction` to `tRNAData`

class `cobrame.core.reaction.`**`tRNAChargingReaction`**(*id*)
Reaction class for the charging of a tRNA with an amino acid

Parameters **`id`** (*`str`*) – Identifier for the charging reaction. As a best practice, ID should follow the template "charging_tRNA + _ + <tRNA_locus> + _ + <codon>". If tRNA initiates translation, <codon> should be replaced with START.

```
In [18]: rxn = cobrame.tRNAChargingReaction('charging_tRNA_d_GUA')
         me.add_reaction(rxn)
         rxn.tRNA_data = data
```

Update tRNAChargingReaction

`tRNAChargingReaction.`**`update`**(*verbose=True*)
Creates reaction using the associated tRNA data

This function adds the following components to the reaction stoichiometry (using 'data' as shorthand for *`cobrame.core.processdata.tRNAData`*):

1. Charged tRNA product following template: "generic_tRNA + _ + <data.codon> + _ + <data.amino_acid>"
2. tRNA metabolite (defined in data.RNA) w/ charging coupling coefficient
3. Charged amino acid (defined in data.amino_acid) w/ charging coupling coefficient

5. Synthetase (defined in data.synthetase) w/ synthetase coupling coefficient found, in part, using data.synthetase_keff
6. Post transcriptional modifications defined in data.subreactions

Parameters **verbose** (*bool*) – Prints when new metabolites are added to the model when executing update()

```
In [19]: #Setting verbose=False suppresses print statements indicating that new metabolites were crea
         rxn.update(verbose=False)
         print(rxn.reaction)
```

```
0.000116266666666667*mu + 4.55184e-5 RNA_d + 0.000116266666666667*mu + 4.55184e-5 val__L_c --> generi
```

This reaction creates one `generic_charged_tRNA` equivalement that can then be used in a translation reaction

The coefficient for `RNA_d` and `lys__L_c` are defined by:

$$v_{dilution,j} \geq \frac{1}{\kappa_\tau c_{tRNA,j}} (\mu + \kappa_\tau r_0) v_{charging,j}, \forall j \in tRNA \tag{3.4}$$

where:

- κ_τ and r_0 are phenomenological parameters from Scott et. al. 2010 that describe the linear relationship between the observed RNA/protein ratio of *E. coli* and its growth rate (μ)
- $c_{tRNA,j} = \frac{m_{tRNA}}{f_{tRNA} \cdot m_{aa}}$ where: m_{tRNA} is molecular weight of an average tRNA. f_{tRNA} is the fraction of total RNA that is tRNA m_{aa} is the molecular weight of an average amino acid
- $v_{charging,j}$ is the rate of charging for $tRNA_j$

Note: This tRNA charging reaction is still missing a tRNA synthetase which catalyzes the amino acid addition to the uncharged tRNA.

Incorporate tRNA Synthetases

.. autoclass:: cobrame.core.component.Complex :noindex:

```
In [20]: synthetase = cobrame.Complex('synthetase')
         me.add_metabolites(synthetase)
```

Associate synthetase with `tRNAData` and update

```
In [21]: data.synthetase = synthetase.id
         rxn.update()
         print(rxn.reaction)
```

```
0.000116266666666667*mu + 4.55184e-5 RNA_d + 4.27350427350427e-6*mu*(0.000116266666666667*mu + 1.0000
```

The synthetase coupling was reformulated from O'brien et. al. 2013 enable more modularity in the ME-model. A more complete mathematical description of the tRNA synthetase coupling constraints can be found in the tRNA.ipynb

3.3.4 Add tRNAs to Translation

Here we take advantage of an additional subclass of `ProcessData`, called a `SubreactionData` object. This class is used to lump together processeses that occur as a result of many individual reactions, including translation elongation, ribosome formation, tRNA modification, etc. Since each of these steps often involve an enzyme that requires its own coupling constraint, this process allows these processes to be lumped into one reaction while still enabling each subprocess to be modified.

`TranslationData` objects have an `subreaction_from_sequence` method that returns any subreactions that have been added to the model and are part of translation elongation (i.e. tRNA). Since no tRNA-mediated amino acid addition subreactions have been added to the model, the below call returns nothing.

```
In [22]: print(me.process_data.a.subreactions_from_sequence)
```

```
{}
```

```
/home/sbrg-cjlloyd/cobrame/cobrame/core/processdata.py:826 UserWarning: tRNA addition subreaction phe
/home/sbrg-cjlloyd/cobrame/cobrame/core/processdata.py:826 UserWarning: tRNA addition subreaction tyr
/home/sbrg-cjlloyd/cobrame/cobrame/core/processdata.py:826 UserWarning: tRNA addition subreaction thr
/home/sbrg-cjlloyd/cobrame/cobrame/core/processdata.py:826 UserWarning: tRNA addition subreaction asp
/home/sbrg-cjlloyd/cobrame/cobrame/core/processdata.py:826 UserWarning: tRNA addition subreaction ser
```

`UserWarnings` are returned to indicate that tRNA subreactions have not been added for each codon.

Below, we add the SubreactionData (excluding enzymes) for the addition of an amino acid using information from the *E. coli* codon table. The charge tRNA does not act as an enzyme in this case because it's coupling is handled in the `tRNAChargingReaction`

Add Subreactions for tRNA addition to model

class `cobrame.core.processdata.`**`SubreactionData`**(*id*, *model*)

Parameters

- **`id`** (*`str`*) – Identifier of the subreaction data. As a best practice, if the subreaction data details a modification, the ID should be prefixed with "mod + _"
- **`model`** (*`cobrame.core.model.MEModel`*) – ME-model that the SubreactionData is associated with

`enzyme`

list or str or None – List of `cobrame.core.component.Complex.id` s for enzymes that catalyze this process

or

String of single `cobrame.core.component.Complex.id` for enzyme that catalyzes this process

`keff`

float – Effective turnover rate of enzyme(s) in subreaction process

`_element_contribution`

dict – If subreaction adds a chemical moiety to a macromolecules via a modification or other means, net element contribution of the modification process should be accounted for. This can be used to mass balance check each of the individual processes.

Dictionary of {element: net_number_of_contributions}

```
In [23]: data = cobrame.SubreactionData('asp_addition_at_GAU', me)
         data.stoichiometry = {'generic_tRNA_GAU_asp__L_c': -1,
                               'gtp_c': -1, 'gdp_c': 1, 'h_c': 1,
                               'pi_c': 1}
```

Now calling `subreactions_from_sequence` returns the number of tRNA subreactions that should be added to the `TranslationData`

```
In [24]: translation_subreactions = me.process_data.a.subreactions_from_sequence
         print(translation_subreactions)
```

```
{'asp_addition_at_GAU': 12}
```

```
/home/sbrg-cjlloyd/cobrame/cobrame/core/processdata.py:826 UserWarning: tRNA addition subreaction phe
/home/sbrg-cjlloyd/cobrame/cobrame/core/processdata.py:826 UserWarning: tRNA addition subreaction tyr
/home/sbrg-cjlloyd/cobrame/cobrame/core/processdata.py:826 UserWarning: tRNA addition subreaction thr
/home/sbrg-cjlloyd/cobrame/cobrame/core/processdata.py:826 UserWarning: tRNA addition subreaction ser
```

Updating `TranslationData.subreactions` with the tRNA subreactions incorporates this information into the `TranslationReaction`

```
In [25]: print("Before adding tRNA subreaction")
         print("------------------------------")
         print(me.reactions.translation_a.reaction)
         print("")
         # Link tranlation_data to subreactions and update
         for subreaction, value in translation_subreactions.items():
             me.process_data.a.subreactions[subreaction] = value
         me.reactions.translation_a.update(verbose=False)
         print("After adding tRNA subreaction")
         print("-----------------------------")
         print(me.reactions.translation_a.reaction)
```

```
Before adding tRNA subreaction
------------------------------
0.000498399634202103*mu + 0.000195123456790123 + 0.00598079561042524*(mu + 0.3915)/mu RNA_a + 12 asp_

After adding tRNA subreaction
-----------------------------
0.000498399634202103*mu + 0.000195123456790123 + 0.00598079561042524*(mu + 0.3915)/mu RNA_a + 12 asp_
```

```
/home/sbrg-cjlloyd/cobrame/cobrame/core/reaction.py:1094 UserWarning: RNA_degradosome not found
/home/sbrg-cjlloyd/cobrame/cobrame/core/processdata.py:229 UserWarning: No element contribution input
```

3.3.5 Add Complex Formation Reaction

Add ComplexData to model

For COBRAme models, the reaction gene-protein-reaction rule (GPR) is replaced with a metabolite representing the synthesis of the enzyme(s) catalyzing a reaction. This metabolite is formed explicitly in a ME model by seperate reaction to transcribe the gene(s) and translate the protein(s) the compose the complex.

class `cobrame.core.processdata.`**`ComplexData`**(*id*, *model*)

Contains all information associated with the formation of an functional enzyme complex.

This can include any enzyme complex modifications required for the enzyme to become active.

Parameters

- **`id`** (*`str`*) – Identifier of the complex data. As a best practice, this should typically use the same ID as the complex being formed. In cases with multiple ways to form complex '_ + alt' or similar suffixes can be used.
- **`model`** (*`cobrame.core.model.MEModel`*) – ME-model that the ComplexData is associated with

stoichiometry
`collections.DefaultDict(int)` – Dictionary containing {protein_id: count} for all protein subunits comprising enzyme complex

subreactions
dict – Dictionary of {subreaction_data_id: count} for all complex formation subreactions/modifications. This can include cofactor/prosthetic group binding or enzyme side group addition.

```
In [26]: data = cobrame.ComplexData('complex_ab', me)
         data.stoichiometry = {'protein_a': 1, 'protein_b': 1}
```

Add ComplexFormation reaction to model

And point `ComplexFormation` to `ComplexData` .. autoclass:: cobrame.core.reaction.ComplexFormation :noindex:

```
In [27]: rxn = cobrame.ComplexFormation('formation_complex_ab')
         me.add_reaction(rxn)
         rxn.complex_data_id = data.id
         rxn._complex_id = data.id
```

Update ComplexFormation reaction

`ComplexFormation.`**`update`**`(`*verbose=True*`)`
Creates reaction using the associated complex data and adds chemical formula to complex metabolite product.

This function adds the following components to the reaction stoichiometry (using 'data' as shorthand for *`cobrame.core.processdata.ComplexData`*):

1. Complex product defined in self._complex_id
2. Protein subunits with stoichiometery defined in data.stoichiometry
3. Metabolites and enzymes w/ coupling coefficients defined in data.subreactions. This often includes enzyme complex modifications by coenzymes or prosthetic groups.
4. Biomass *`cobrame.core.component.Constraint`* corresponding to modifications detailed in data.subreactions, if any

Parameters **verbose** (*bool*) – Prints when new metabolites are added to the model when executing update()

```
In [28]: rxn.update(verbose=False)
         print(me.reactions.formation_complex_ab.reaction)
```

```
protein_a + protein_b --> complex_ab
```

Apply modification to complex formation reaction

Many enzyme complexes in an ME-model require cofactors or prosthetic groups in order to properly function. Information about such processes are stored as ModificationData.

For instance, we can add the modification of an iron-sulfur cluster, a common prosthetic group, by doing the following:

```
In [29]: # Define the stoichiometry of the modification
         mod_data = cobrame.SubreactionData('mod_2fe2s_c', me)
         mod_data.stoichiometry = {'2fe2s_c': -1}
         # this process can also be catalyzed by a chaperone
```

```
        mod_data.enzyme = 'complex_ba'
        mod_data.keff = 65.  # default value
```

Associate modification to complex and `update()` its formation

```
In [30]: complex_data = me.process_data.complex_ab
         complex_data.subreactions['mod_2fe2s_c'] = 1
```

Update ComplexFormation reaction

```
In [31]: print('Before adding modification')
         print('---------------------------')
         print(me.reactions.formation_complex_ab.reaction)
         me.reactions.formation_complex_ab.update()
         print('\n')
         print('After adding modification')
         print('-------------------------')
         print(me.reactions.formation_complex_ab.reaction)
```

```
Before adding modification
--------------------------
protein_a + protein_b --> complex_ab
Created <Complex complex_ba at 0x7f4bf69a8b38> in <ComplexFormation formation_complex_ab at 0x7f4bf5


After adding modification
-------------------------
2fe2s_c + 4.27350427350427e-6*mu complex_ba + protein_a + protein_b --> complex_ab + 0.17582 prosthet
```

3.3.6 Add Metabolic Reaction

Add StoichiometricData to model

MetabolicReactions require, at a minimum, one corresponding StoichiometricData. StoichiometricData essentially holds the information contained in an M-model reaction. This includes the metabolite stoichiometry and the upper and lower bound of the reaction. As a best practice, StoichiometricData typically uses an ID equivalent to the M-model reaction ID.

So first, we will create a StoichiometricData object to define the stoichiometry of the conversion of *a* to *b*. **Only one StoichiometricData object should be created for both reversible and irreversible reactions**

class `cobrame.core.processdata.`**`StoichiometricData`**(*id*, *model*)

Encodes the stoichiometry for a metabolic reaction.

StoichiometricData defines the metabolite stoichiometry and upper/lower bounds of metabolic reaction

Parameters

- **`id`** (*`str`*) – Identifier of the metabolic reaction. Should be identical to the M-model reactions in most cases.
- **`model`** (*`cobrame.core.model.MEModel`*) – ME-model that the StoichiometricData is associated with

`_stoichiometry`

dict – Dictionary of {metabolite_id: stoichiometry} for reaction

`subreactions`

`collections.DefaultDict(int)` – Cases where multiple enzymes (often carriers ie. Acyl Carrier Protein) are involved in a metabolic reactions.

upper_bound
int – Upper reaction bound of metabolic reaction. Should be identical to the M-model reactions in most cases.

lower_bound
int – Lower reaction bound of metabolic reaction. Should be identical to the M-model reactions in most cases.

```
In [32]: # unique to COBRAme, construct a stoichiometric data object with the reaction information
         data = cobrame.StoichiometricData('a_to_b', me)
         stoichiometry = {'a':-1, 'b': 1}
         data._stoichiometry = stoichiometry
         data.lower_bound = -1000
         data.upper_bound = 1000
```

Add MetabolicReaction to model

The StoichiometricData for this reversible reaction is then assigned to two different MetabolicReactions (Due to the enzyme dilution constraint, all enzyme catalyzed reactions must be reverisble; more on this later). The MetabolicReactions require: - The associated StoichiometricData - The *reverse* flag set to True for reverse reactions, False for forward reactions - Enzyme K_{eff} for reaction (discussed later, dafault=65)

These fields are then processed and the actual model reaction is created using the MetabolicReaction's update() function

class cobrame.core.reaction.**MetabolicReaction**(*id*)
Irreversible metabolic reaction including required enzymatic complex

This reaction class's update function processes the information contained in the complex data for the enzyme that catalyzes this reaction as well as the stoichiometric data which contains the stoichiometry of the metabolic conversion being performed (i.e. the stoichiometry of the M-model reaction analog)

Parameters **id** (*str*) – Identifier of the metabolic reaction. As a best practice, this ID should use the following template (FWD=forward, REV=reverse): "<StoichiometricData.id> + _ + <FWD or REV> + _ + <Complex.id>"

keff
float – The turnover rete (keff) couples enzymatic dilution to metabolic flux

reverse
boolean – If True, the reaction corresponds to the reverse direction of the reaction. This is necessary since all reversible enzymatic reactions in an ME-model are broken into two irreversible reactions

```
In [33]: # Create a forward ME Metabolic Reaction and associate the stoichiometric data to it
         rxn_fwd = cobrame.MetabolicReaction('a_to_b_FWD_complex_ab')
         me.add_reaction(rxn_fwd)
         rxn_fwd.stoichiometric_data = data
         rxn_fwd.reverse = False
         rxn_fwd.keff = 65.

         # Create a reverse ME Metabolic Reaction and associate the stoichiometric data to it
         rxn_rev = cobrame.MetabolicReaction('a_to_b_REV_complex_ab')
         me.add_reaction(rxn_rev)
         rxn_rev.stoichiometric_data = data
         rxn_rev.reverse = True
         rxn_rev.keff = 65.
```

Update MetabolicReactions

`MetabolicReaction.update(verbose=True)`

Creates reaction using the associated stoichiometric data and complex data.

This function adds the following components to the reaction stoichiometry (using 'data' as shorthand for *cobrame.core.processdata.StoichiometricData*):

1. Complex w/ coupling coefficients defined in self.complex_data.id and self.keff
2. Metabolite stoichiometry defined in data.stoichiometry. Sign is flipped if self.reverse == True

Also sets the lower and upper bounds based on self.reverse and data.upper_bound and data.lower_bound.

Parameters **verbose** (*bool*) – Prints when new metabolites are added to the model when executing update()

```
In [34]: rxn_fwd.update(verbose=False)
         rxn_rev.update(verbose=False)
         print(me.reactions.a_to_b_FWD_complex_ab.reaction)
         print(me.reactions.a_to_b_REV_complex_ab.reaction)
```

```
a --> b
b --> a
```

Note: the k_{eff} and `complex_ab` is not included in the reaction since no complex has been associated to it yet

Associate enzyme with MetabolicReaction

The ComplexData object created in the previous cell can be incorporated into the MetabolicReaction using code below.

Note: the update() function is required to apply the change.

```
In [35]: data = me.process_data.complex_ab
         me.reactions.a_to_b_FWD_complex_ab.complex_data = data
         print('Forward reaction (before update): %s' %
               (me.reactions.a_to_b_FWD_complex_ab.reaction))
         me.reactions.a_to_b_FWD_complex_ab.update()
         print('Forward reaction (after update): %s' %
               (me.reactions.a_to_b_FWD_complex_ab.reaction))
         print('')

         me.reactions.a_to_b_REV_complex_ab.complex_data = data
         print('Reverse reaction (before update): %s' %
               (me.reactions.a_to_b_REV_complex_ab.reaction))
         me.reactions.a_to_b_REV_complex_ab.update()
         print('Reverse reaction (after update): %s' %
               (me.reactions.a_to_b_REV_complex_ab.reaction))
```

```
Forward reaction (before update): a --> b
Forward reaction (after update): a + 4.27350427350427e-6*mu complex_ab --> b

Reverse reaction (before update): b --> a
Reverse reaction (after update): b + 4.27350427350427e-6*mu complex_ab --> a
```

The coefficient for complex_ab is determined by the expression

$$\frac{\mu}{k_{eff}}$$

which in its entirety represents the dilution of an enzyme following a cell doubling. The coupling constraint can be summarized as followed

$$v_{dilution,j} = \mu \sum_i \left(\frac{1}{k_{eff,i}} v_{usage,i} \right), \qquad \forall j \in Enzyme \tag{3.5}$$

Where

- $v_{usage,i}$ is the flux through the metabolic reaction
- k_{eff} is the turnover rate for the process and conveys the productivity of the enzyme complex. Physically, it can be thought of as the number of reactions the enzyme can catalyze per cell division.

By default the k_{eff} for a MetabolicReaction is set to 65 but this can be changed using the code below.

Different Keff for forward reaction

```
In [36]: me.reactions.a_to_b_FWD_complex_ab.keff = .00001
         me.reactions.a_to_b_FWD_complex_ab.update()

         # The forward and reverse direction can have differing keffs
         print('Forward reaction')
         print('-----------------')
         print(me.reactions.a_to_b_FWD_complex_ab.reaction)
         print('')
         print('Reverse reaction')
         print('-----------------')
         print(me.reactions.a_to_b_REV_complex_ab.reaction)
```

```
Forward reaction
----------------
a + 27.7777777777778*mu complex_ab --> b

Reverse reaction
----------------
b + 4.27350427350427e-6*mu complex_ab --> a
```

3.4 Adding Reactions using utility functions

Add reactions using some of the utility functions provided in `cobrame.util.building.py`

3.4.1 Transcription

Using the utility functions to create the TranscribedGene metabolite has the advantage of forcing the assignment of sequence, strand and RNA_type.

`cobrame.util.building.`**`create_transcribed_gene`**(*me_model, locus_id, rna_type, seq, left_pos=None, right_pos=None, strand=None*)

Creates a *TranscribedGene* metabolite object and adds it to the ME-model

Parameters

- **me_model** (*cobrame.core.model.MEModel*) – The MEModel object to which the reaction will be added
- **locus_id** (*str*) – Locus ID of RNA product. The TranscribedGene will be added as "RNA + _ + locus_id"
- **left_pos** (*int or None*) – Left position of gene on the sequence of the (+) strain
- **right_pos** (*int or None*) – Right position of gene on the sequence of the (+) strain
- **seq** (*str*) – Nucleotide sequence of RNA product. Amino acid sequence, codon counts, etc. will be calculated based on this string.
- **strand** (*str or None*) –
  - (+) if the RNA product is on the leading strand
  - (-) if the RNA product is on the complementary strand
- **rna_type** (*str*) – Type of RNA of the product. tRNA, rRNA, or mRNA Used for determining how RNA product will be processed.

Returns Metabolite object for the RNA product

Return type *cobrame.core.component.TranscribedGene*

```
In [37]: building.create_transcribed_gene(me, 'b','tRNA', 'ATCG')
         building.add_transcription_reaction(me, 'TU_b', {'b'}, sequence)
         print(me.reactions.transcription_TU_b.reaction)
         me.reactions.transcription_TU_b.update()
```

```
86 atp_c + 38 ctp_c + 12 gtp_c + 182 h2o_c + 50 utp_c --> RNA_b + 85 amp_c + 37 cmp_c + 11 gmp_c + 18

/home/sbrg-cjlloyd/cobrame/cobrame/core/reaction.py:813 UserWarning: RNA Polymerase () not found
```

3.4.2 Translation

`add_translation_reaction` assumes that the RNA and protein have the same locus_id. It creates the appropriate `TranslationData` and `TranslationReaction` instance, links the two together and updates the `TranslationReaction`.

`cobrame.util.building.`**`add_translation_reaction`**(*me_model*, *locus_id*, *dna_sequence*, *update=False*)

Creates and adds a TranslationReaction to the ME-model as well as the associated TranslationData

A dna_sequence is required in order to add a TranslationReaction to the ME-model

Parameters

- **me_model** (`cobra.core.model.MEModel`) – The MEModel object to which the reaction will be added
- **locus_id** (*str*) – Locus ID of RNA product. The TranslationReaction will be added as "translation + _ + locus_id" The TranslationData will be added as "locus_id"
- **dna_sequence** (*str*) – DNA sequence of the RNA product. This string should be reverse transcribed if it originates on the complement strand.
- **update** (*bool*) – If True, use TranslationReaction's update function to update and add reaction stoichiometry

```
In [38]: building.add_translation_reaction(me, 'b', dna_sequence=sequence, update=True)
         print(me.reactions.translation_b.reaction)
```

```
/home/sbrg-cjlloyd/cobrame/cobrame/core/reaction.py:1094 UserWarning: RNA_degradosome not found
0.000498399634202103*mu + 0.000195123456790123 + 0.00598079561042524*(mu + 0.3915)/mu RNA_b + 12 asp_
```

3.4.3 Complex Formation

Alternatively, ComplexData has a `create_complex_formation()` function to create the sythesis reaction following the naming conventions. It contains an `update()` function which incorporates changes in the ComplexData

`ComplexData.`**`create_complex_formation`**`(`*verbose=True*`)`

creates a complex formation reaction

This assumes none exists already. Will create a reaction (prefixed by "formation") which forms the complex

Parameters **`verbose`** (*bool*) – If True, print if a metabolite is added to model during update

```
In [39]: data = cobrame.ComplexData('complex_ba', me)
         data.stoichiometry = {'protein_a': 1, 'protein_b': 1}
         data.create_complex_formation()
         print(me.reactions.formation_complex_ba.reaction)
```

```
protein_a + protein_b --> complex_ba
```

3.4.4 Metabolic Reaction

`cobrame.util.building.`**`add_metabolic_reaction_to_model`**`(`*me_model*, *stoichiometric_data_id*, *directionality*, *complex_id=None*, *spontaneous=False*, *update=False*, *keff=65*`)`

Creates and add a MetabolicReaction to a MEModel.

Parameters

- **`me_model`** (*cobrame.core.model.MEModel*) – MEModel that the MetabolicReaction will be added to
- **`stoichiometric_data_id`** (*str*) – ID of the StoichiometricData for the reaction being added
- **`directionality`** (*str*) –
  - Forward: Add reaction that occurs in the forward direction
  - Reverse: Add reaction that occurs in the reverse direction
- **`complex_id`** (*str or None*) – ID of the ComplexData for the enzyme that catalyze the reaction being added.
- **`spontaneous`** (*bool*) –
  - If True and complex_id=" add reaction as spontaneous reaction
  - If False and complex_id=" add reaction as orphan (CPLX_dummy catalyzed)

```
In [40]: stoich_data = cobrame.StoichiometricData('b_to_c', me)
         stoich_data._stoichiometry = {'b': -1, 'c': 1}
         stoich_data.lower_bound = 0
         stoich_data.upper_bound = 1000.
         building.add_metabolic_reaction_to_model(me, stoich_data.id, 'forward', complex_id='complex_
                                                  update=True)
         print('Reaction b_to_c')
```

```
    print('---------------')
    print(me.reactions.b_to_c_FWD_complex_ab.reaction)
```

```
Created <Metabolite c at 0x7f4bf6abf6d8> in <MetabolicReaction b_to_c_FWD_complex_ab at 0x7f4bf6abf7b
Reaction b_to_c
---------------
b + 4.27350427350427e-6*mu complex_ab --> c
```

CHAPTER 4

Reaction Properties

The division of the ME-model into MEReaction and ProcessData classes allows the user to essentially have access to the entire database of information used to construct the model. The following will show how this can be leveraged to easily query, edit and update aspects of the model reactions.

```
In [1]: import pickle
        from collections import defaultdict
        from os.path import abspath, dirname, join

        import pandas as pd
        import cobra.test

        import cobrame
        import ecolime
```

```
/home/sbrg-cjlloyd/cobrapy/cobra/io/sbml3.py:24: UserWarning: Install lxml for faster SBML I/O
  warn("Install lxml for faster SBML I/O")
/home/sbrg-cjlloyd/cobrapy/cobra/io/__init__.py:12: UserWarning: cobra.io.sbml requires libsbml
  warn("cobra.io.sbml requires libsbml")
```

```
In [2]: # Load E. coli ME-model
        ecoli_dir = dirname(abspath(ecolime.__file__))
        model_dir = join(ecoli_dir, 'me_models/iJL1678b.pickle')
        with open(model_dir, 'rb') as f:
            me = pickle.load(f)

        # Load E. coli M-model
        iJO1366 = cobra.test.create_test_model('ecoli')
```

4.1 Metabolic Reactions

These are the ME-model representations of all reactions in the metabolic reconstruction, in this case iJO1366

```
In [3]: print('number of reactions in iJO1366 (excluding exchange) = %i' %
              len([i.id for i in iJO1366.reactions if not i.id.startswith('EX_')]))
```

```
        print('number of stoichiometric data objects = %i\n' %
              len([r.id for r in me.stoichiometric_data]))
        print('number of metabolic reactions = %i' %
              len([r.id for r in me.reactions if type(r) == cobrame.MetabolicReaction]))
        print('number of complex data objects = %i' %
              len([r.id for r in me.complex_data]))
```

```
number of reactions in iJO1366 (excluding exchange) = 2259
number of stoichiometric data objects = 2282

number of metabolic reactions = 5266
number of complex data objects = 1445
```

Through a MetabolicReaction, the user has direct access to the StoichiometricData, ComplexData and keff used to construct the reaction.

```
In [4]: rxn = me.reactions.get_by_id('E4PD_FWD_GAPDH-A-CPLX')

        # Access the StoichiometricData and ComplexData directly through reaction
        stoich_data = rxn.stoichiometric_data
        complex_data = rxn.complex_data

        print(rxn.reaction + '\n')
        print('This reactions is formed using the StoichiometricData (%s) and ComplexData (%s) with k
              (stoich_data.id, complex_data.id, rxn.keff))
```

```
3.20942472231275e-6*mu GAPDH-A-CPLX + e4p_c + h2o_c + nad_c --> 4per_c + 2.0 h_c + nadh_c

This reactions is formed using the StoichiometricData (E4PD) and ComplexData (GAPDH-A-CPLX) with kef
```

As a best practice, the ComplexData and StoichiometricData themselves should not be changed. If these need changed then a new MetabolicReaction should be created.

4.1.1 Edit keffs

Further, the keff is an attribute of the MetabolicReaction itself and not of the ComplexData changing the ComplexData will not affect the form of the MetabolicReaction.

```
In [5]: print('keff = %d: \n\t%s' % (rxn.keff, rxn.reaction))
        rxn.keff = 65.
        rxn.update()
        print('keff = %d: \n\t%s' % (rxn.keff, rxn.reaction))
```

```
keff = 86:
        3.20942472231275e-6*mu GAPDH-A-CPLX + e4p_c + h2o_c + nad_c --> 4per_c + 2.0 h_c + nadh_c
keff = 65:
        4.27350427350427e-6*mu GAPDH-A-CPLX + e4p_c + h2o_c + nad_c --> 4per_c + 2.0 h_c + nadh_c
```

4.1.2 Edit stoichiometry

Aspects of the StoichiometricData, however, can be changed. This includes: - Reaction stoichiometry - Reaction upper & lower bounds

Currently the stoichiometry is:

```
In [6]: stoich_data.stoichiometry

Out[6]: {'4per_c': 1.0,
         'e4p_c': -1.0,
         'h2o_c': -1.0,
```

```
 'h_c': 2.0,
 'nad_c': -1.0,
 'nadh_c': 1.0}
```

This can be updated to, for instance, translocate a hydrogen by performing the following

```
In [7]: stoich_data.stoichiometry['h_c'] = -1
        stoich_data.stoichiometry['h_p'] = 1
        rxn.update()
        print('%s: \n\t%s' % (rxn.id, rxn.reaction))
```

```
E4PD_FWD_GAPDH-A-CPLX:
        4.27350427350427e-6*mu GAPDH-A-CPLX + e4p_c + h2o_c + h_c + nad_c --> 4per_c + h_p + nadh_c
```

This change can be usd to update both the forward and reverse reaction

```
In [8]: rxn_rev = me.reactions.get_by_id(rxn.id.replace('FWD', 'REV'))
        rxn_rev.update()
        print('%s: \n\t%s' % (rxn_rev.id, rxn_rev.reaction))
```

```
E4PD_REV_GAPDH-A-CPLX:
        4per_c + 3.20942472231275e-6*mu GAPDH-A-CPLX + h_p + nadh_c --> e4p_c + h2o_c + h_c + nad_c
```

A simpler approach is to updated the parent reactions for StoichiometricData. This will update any instances of the reaction catalyzed by an isozyme.

```
In [9]: stoich_data.stoichiometry['h_c'] = -2
        stoich_data.stoichiometry['h_p'] = 2
        for r in stoich_data.parent_reactions:
            r.update()
            print('%s: \n\t%s' % (r.id, r.reaction))
```

```
E4PD_FWD_ERYTH4PDEHYDROG-CPLX:
        0.0135143204065698*mu ERYTH4PDEHYDROG-CPLX + e4p_c + h2o_c + 2.0 h_c + nad_c --> 4per_c + 2.(
E4PD_FWD_GAPDH-A-CPLX:
        4.27350427350427e-6*mu GAPDH-A-CPLX + e4p_c + h2o_c + 2.0 h_c + nad_c --> 4per_c + 2.0 h_p +
E4PD_REV_ERYTH4PDEHYDROG-CPLX:
        4per_c + 3.09490345954754e-6*mu ERYTH4PDEHYDROG-CPLX + 2.0 h_p + nadh_c --> e4p_c + h2o_c + 2
E4PD_REV_GAPDH-A-CPLX:
        4per_c + 3.20942472231275e-6*mu GAPDH-A-CPLX + 2.0 h_p + nadh_c --> e4p_c + h2o_c + 2.0 h_c +
```

4.1.3 Edit upper and lower reaction bounds

The upper and lower bounds can be edited through the stoichiometric data and updated to the metabolic reaction

Important: do not change the upper and lower bounds of a MetabolicReaction directly. If this is done than the change will be overwritten when the update function is ran (shown below)

```
In [10]: rxn.lower_bound = -1000
         print('Lower bound = %d' %rxn.lower_bound)
         rxn.update()
         print('Lower bound = %d' %rxn.lower_bound)
```

```
Lower bound = -1000
Lower bound = 0
```

Editing the reaction bounds of the StoichiometricData, however, will edit the bounds of the forward and reverse reaction, as well as any instances of the reaction catalyzed by isozymes

```
In [11]: stoich_data.lower_bound = 0.
         print('Upper Bounds\n--------------------------------------')
         for r in stoich_data.parent_reactions:
             direction = 'Forward' if r.reverse is False else 'Reverse'
```

```
            print('%s Before Update \n\t%s: %s' % (direction, r.id, r.upper_bound))
            r.update()
            print('%s After Update \n\t%s: %s' % (direction, r.id, r.upper_bound))
```

```
Upper Bounds
--------------------------------------
Forward Before Update
        E4PD_FWD_ERYTH4PDEHYDROG-CPLX: 1000.0
Forward After Update
        E4PD_FWD_ERYTH4PDEHYDROG-CPLX: 1000.0
Forward Before Update
        E4PD_FWD_GAPDH-A-CPLX: 1000.0
Forward After Update
        E4PD_FWD_GAPDH-A-CPLX: 1000.0
Reverse Before Update
        E4PD_REV_ERYTH4PDEHYDROG-CPLX: 1000.0
Reverse After Update
        E4PD_REV_ERYTH4PDEHYDROG-CPLX: 0
Reverse Before Update
        E4PD_REV_GAPDH-A-CPLX: 1000.0
Reverse After Update
        E4PD_REV_GAPDH-A-CPLX: 0
```

4.2 Transcription Reactions

```
In [12]: print('number of transcription reactions = %i' %
               len([r.id for r in me.reactions if type(r) == cobrame.TranscriptionReaction]))
         print('number of transcription data objects = %i' %  len(list(me.transcription_data)))
         print('number of transcribed genes (RNA) = %i' %
               len([m.id for m in me.metabolites if type(m) == cobrame.TranscribedGene]))
```

```
number of transcription reactions = 1447
number of transcription data objects = 1447
number of transcribed genes (RNA) = 1679
```

4.2.1 TranscribedGene (RNA) metabolite properties

Transciption occurs via operons contained within the organisms genome or transcription unit (TU). This means that often, a transcribed region will code for multiple RNAs. The E. coli ME-model has 4 possible RNA types that can be transcribed: - mRNA - tRNA - rRNA - ncRNA (noncoding RNA)

mRNAs can then translated directly from the full transcribed TU, while rRNA, tRNA and ncRNA are spliced out of the TU by endonucleases. In these cases, in order to know which bases need excized, the RNA metabolites (TranscribedGene) themselves have to store information such as: - **DNA strand, left and right genome position** to identify which TU the RNA is a part of - **RNA type** to determine whether it needs excised from the TU - **nucleotide sequence** to determine bases that do/do not need excised if not mRNA and the RNA mass for biomass constraint

An example of a TranscribedGene's attributes is shown below

```
In [13]: pd.DataFrame({i: str(v) for i, v in me.metabolites.RNA_b3201.__dict__.items() if not i.start
                      index=['Atribute Values']).T
```

```
Out[13]: Atribute Values
         RNA_type                                              mRNA
         formula                              C6890H7816N2720O5091P726
         id                                               RNA_b3201
```

```
left_pos                                                          3341965
nucleotide_sequence  ATGGCAACATTAACTGCAAAGAACCTTGCAAAAGCCTATAAAGGCC...
right_pos                                                         3342691
strand                                                                  +
```

4.2.2 TranscriptionReaction/TranscriptionData properties

Each TranscriptionReaction in a COBRAme ME-model is associated with exactly one TranscriptionData which includes everything necessary to define a reaction. This includes: - **subreactions** To handle enzymatic processes not performed by RNA polymerase - **RNA Polymerase** Different RNA polymerase metabolite for different sigma factors - **RNA Products** TUs often contain more than one RNA in sequence - **Nucleotide sequence**

The TranscriptionData for TU containing the gene above is shown below

```
In [14]: rxn = me.reactions.transcription_TU_8398_from_RPOE_MONOMER
         data = rxn.transcription_data
         pd.DataFrame({i: str(v) for i, v in data.__dict__.items()}, index=['Atribute Values']).T
```

```
Out[14]: Atribute Values
         RNA_polymerase                                             RNAPE-CPLX
         RNA_products                                 {'RNA_b3201', 'RNA_b3202'}
         _model                                                     iJL1678b-ME
         _parent_reactions          {'transcription_TU_8398_from_RPOE_MONOMER'}
         id                                             TU_8398_from_RPOE_MONOMER
         nucleotide_sequence  ACAAACTCAGCCTTAATCTTGTGCTTGCCAGCTCACTTCTGGCCGC...
         subreactions         defaultdict(<class 'int'>, {'Transcription_nor...
```

This reaction currently uses a subreaction called *Transcription_normal_rho_dependent* to account for the elongation factors etc. associated with transcription. This TU also requires a rho factor to terminate transcription. These complexes can be removed from the reaction by running the following

```
In [15]: print('with subreactions: \n' + rxn.reaction)
         print('--------------------')
         data.subreactions = {}
         for r in data.parent_reactions:
             r.update()
         print('\nwithout subreactions: \n' + rxn.reaction)
```

```
with subreactions:
4.27350427350427e-6*mu GreA_mono + 4.27350427350427e-6*mu GreB_mono + 4.27350427350427e-6*mu Mfd_mono
--------------------

without subreactions:
0.0218585689888099*mu + 0.00855762975911906 RNAPE-CPLX + 1017 atp_c + 1181 ctp_c + 1190 gtp_c + 1186
```

This poses a problem where, if RNA_b3201 and RNA_b3202 are not required in equal amounts, the model will become infeasible. To accound for this, all RNAs have a demand reaction associated with them. *mRNA_biomass* is consumed for each demand reaction with a coefficient equal to the molecular weight of each RNA (in kDa). This prevents the model from overproducing RNA to increase biomass production, and therefore growth rate, in some instances. More on the implications of the *biomass* constraint can be found in **ME-Model Fundamentals**

```
In [16]: for rna in data.RNA_products:
             r = me.reactions.get_by_id('DM_' + rna)
             print('%s: %s' % (r.id, r.reaction))
```

```
DM_RNA_b3201: RNA_b3201 + 232.671391 mRNA_biomass -->
DM_RNA_b3202: RNA_b3202 + 459.03124199999996 mRNA_biomass -->
```

As is, this reaction produces two mRNAs so no nucleotides are excised. If one or both is changed to a stable RNA (rRNA, tRNA or ncRNA) bases will be excised.

```
In [17]: me.metabolites.RNA_b3202.RNA_type = 'rRNA'
         for r in data.parent_reactions:
             r.update()
             print(r.reaction)
```

```
0.0218585689888099*mu + 0.00855762975911906 RNAPE-CPLX + 1017 atp_c + 1181 ctp_c + 1190 gtp_c + 2414
```

Changing RNA_b3202 to an rRNA and updating the transcription reaction causes both of the RNAs to now be excised from the TU, as indicated by the nucleotide monophosphates that appear in the products. This is not a complete picture because this process is catalyzes by an endonuclease, whose activity can be incorporated as ModificationData. Updating the reaction after adding these processes incorporates

```
In [18]: data.subreactions['rRNA_containing_excision'] = len(data.RNA_products) * 2
         data.subreactions['RNA_degradation_machine'] = len(data.RNA_products) * 2
         data.subreactions['RNA_degradation_atp_requirement'] = sum(data.excised_bases.values())
         for r in data.parent_reactions:
             r.update()
             print(r.reaction)
```

```
0.0218585689888099*mu + 0.00855762975911906 RNAPE-CPLX + 1.70940170940171e-5*mu RNA_degradosome + 162
```

4.3 Translation Reactions

```
In [19]: print('number of translation reactions = %i' %
               len([r.id for r in me.reactions if type(r) == cobrame.TranslationReaction]))
         print('number of translation data objects = %i' %  len(list(me.translation_data)))
         print('number of translated genes (proteins) = %i' %
               len([m.id for m in me.metabolites if type(m) == cobrame.TranslatedGene]))
```

```
number of translation reactions = 1569
number of translation data objects = 1569
number of translated genes (proteins) = 1569
```

4.3.1 TranslatedGene (Protein) metabolite properties

For COBRAme ME-models, proteins are translated directly from mRNA metabolites not from TUs. This means that all information required to construct a TranslationReaction can be found in its TranslationData therefore no extra information is contained in a TranslatedGene object.

4.3.2 TranslationReaction/TranslationData properties

Each TranslationReaction in a COBRAme ME-model is associated with exactly one TranslationData which includes everything necessary to define the reaction. This includes: - **subreactions** To handle enzymatic processes not performed by ribosome and incorporate tRNAs - **mRNA** ID of mRAN being translated - **term_enzyme** Enzyme that catalyzes translation termination - **Nucleotide sequence**

The TranslationData for a TranslationReaction is shown below

```
In [20]: rxn = me.reactions.translation_b2020
         data = rxn.translation_data
         pd.DataFrame({i: str(v) for i, v in data.__dict__.items()}, index=['Atribute Values']).T
```

```
Out[20]: Atribute Values
         _model                                                   iJL1678b-ME
         _parent_reactions                                  {'translation_b2020'}
```

```
id                                                                 b2020
mRNA                                                           RNA_b2020
nucleotide_sequence  ATGAGCTTTAACACAATCATTGACTGGAATAGCTGTACTGCGGAGC...
protein                                                    protein_b2020
subreactions         defaultdict(<class 'int'>, {'met_addition_at_A...
```

The rest of the information required to define a translation reaction can be dynamically computed from these attributes.

For example, the amino acid sequence is calculated from the nucleotide sequence with:

```
In [21]: str(data.amino_acid_count)
```

```
Out[21]: "defaultdict(<class 'int'>, {'met__L_c': 7, 'ser__L_c': 34, 'phe__L_c': 12, 'asn__L_c': 13,
```

The codon count from the sequence can be used to determine the subreaction required for charged tRNA-mediated amino acid addition.

```
In [22]: str(data.subreactions_from_sequence)
```

```
Out[22]: "{'met_addition_at_AUG': 6, 'ser_addition_at_AGC': 12, 'phe_addition_at_UUU': 6, 'asn_additi
```

Changing the nucleotide sequence will automically update these values.

```
In [23]: data.nucleotide_sequence = 'ATGAGCTTTAAC'
         print('Elongation Subreactions')
         print(str(data.subreactions_from_sequence))
         print('\nOne of each start subreaction')
         print(str(data.add_initiation_subreactions()))
         print('\nNo termination subreaction (AAC) not valid stop codon')
         print(str(data.add_termination_subreactions()))
```

```
Elongation Subreactions
{'ser_addition_at_AGC': 1, 'phe_addition_at_UUU': 1}

One of each start subreaction
None

No termination subreaction (AAC) not valid stop codon
None
```

```
/home/sbrg-cjlloyd/cobrame/cobrame/core/processdata.py:888 UserWarning: RNA_b2020 starts with 'AUG' w
/home/sbrg-cjlloyd/cobrame/cobrame/core/processdata.py:925 UserWarning: No termination enzyme for RNA
```

Stop codons are defined for the organism beforehand. Changing the sequence to a valid stop codon corrects this

```
In [24]: data.nucleotide_sequence = 'ATGAGCTTTTAA'
         print('Termination subreaction')
         print(str(data.add_termination_subreactions()))
```

```
Termination subreaction
None
```

```
/home/sbrg-cjlloyd/cobrame/cobrame/core/processdata.py:925 UserWarning: No termination enzyme for RNA
```

4.4 ComplexFormation Reactions

```
In [25]: print('number of complex formation reactions = %i' %
               len([r.id for r in me.reactions if type(r) == cobrame.ComplexFormation]))
         print('number of complex data objects = %i' %  len(list(me.complex_data)))
         print('')
```

```
        print('number of complexes = %i' %
              len([m.id for m in me.metabolites if type(m) == cobrame.Complex]))
```

```
number of complex formation reactions = 1445
number of complex data objects = 1445

number of complexes = 1538
```

some complexes are modified in a metabolic process (e.g. Acyl Carrier Protein sidechain reactions) ### Complex Metabolite Properties Like TranslatedGenes, Complex metabolites do not need to store any additional information. ### ComplexFormation / ComplexData Properties Each ComplexFormation reaction in a COBRAme ME-model is associated with exactly one ComplexData which includes everything necessary to define the reaction. This includes: - **modification** To define the modifications by prosthetic groups or cofactors that can be required for the complex to catalyze cellular processes - **stoichiometry** Stoichiometry of the protein subunits

The ComplexData for a ComplexFormation reaction is shown below

```
In [26]: rxn = me.reactions.get_by_id('formation_2OXOGLUTARATEDEH-CPLX_mod_mg2_mod_lipo')
         data = me.process_data.get_by_id(rxn.complex_data_id)
         pd.DataFrame({i: str(v) for i, v in data.__dict__.items()}, index=['Atribute Values']).T
```

```
Out[26]: Atribute Values
         _complex_id                                                    None
         _model                                                  iJL1678b-ME
         _parent_reactions  {'AKGDH_FWD_2OXOGLUTARATEDEH-CPLX_mod_mg2_mod_...
         id                           2OXOGLUTARATEDEH-CPLX_mod_mg2_mod_lipo
         stoichiometry      defaultdict(<class 'float'>, {'protein_b0726':...
         subreactions                  {'mod_mg2_c': 1.0, 'mod_lipo_c': 1.0}
```

This complex has two modification mod_lipo_c and mod_mg_c. You can view the properties of these modifications by accessing their ModificationData objects.

```
In [27]: for mod in data.subreactions:
             mod_data = me.process_data.get_by_id(mod)
             print(mod_data.id)
             for key, value in mod_data.__dict__.items():
                 if not key.startswith('_') and value:
                     print('\t', key, value)
```

```
mod_mg2_c
         id mod_mg2_c
         stoichiometry {'mg2_c': -1}
         keff 65.0
mod_lipo_c
         id mod_lipo_c
         stoichiometry {'lipoamp_c': -1, 'amp_c': 1, 'h_c': 2}
         enzyme EG11796-MONOMER
         keff 65.0
```

The information in the ModificationData and ComplexData are assembled in the ComplexFormation reaction shown below

```
In [28]: print(rxn.reaction)
```

```
4.27350427350427e-6*mu EG11796-MONOMER + lipoamp_c + mg2_c + 2.0 protein_b0116 + 12.0 protein_b0726 +
```

CHAPTER 5

ME-model Saving and Loading

There are currently 3 methods that can be used to save/load an ME-model using COBRAme: 1. As a full JSON file 2. As a reduced JSON file 3. As a pickle file

5.1 As a full JSON file

This is the recommended way to save, load and share `COBRAme` ME-models in full detail. This will include all of the model's functionality and information (`MEReaction`, `ProcessData`, etc). It uses a defined JSONSCHEMA found in `cobrame.io`.

Saving and loading a full ME-model (`me_model`) as a JSON can be done using:

```
In [ ]: from cobrame.io.json import save_json_me_model, load_json_me_model
        save_json_me_model(me_model, '[save_loc]/model.json')
```

Then loading can be done with

```
In [ ]: new_me_model = load_json_me_model('[save_loc]/model.json')
```

where `new_me_model` is of type `cobrame.MEModel`

5.2 As a reduced JSON file

Alternatively, ME-models can be saved as a `COBRApy` model. This storage type loses all the additional information contained in a full ME-model, but retains the stoichiometry of all the reactions. In other words, it behaves like an M-model with symbolic mu terms in metabolic coefficients and reaction bounds. Therefore it will give identical solutions compared to the full model, but all additional ME-model functionality will be lost.

Saving and loading a reduced Me-model (`me_model`) as a JSON can be done using:

```
In [ ]: from cobrame.io.json import save_reduced_json_me_model, load_reduced_json_me_model
        save_reduced_json_me_model(me_model, '[save_loc]/model.json')
```

Then loading can be done with

```
In [ ]: new_me_model = load_reduced_json_me_model('[save_loc]/model.json')
```

where `new_me_model` is of type `cobra.Model`

5.3 As a pickle file

This is the quickest way to save a ME-model in full detail. It can be accomplished using python's pickle dump/load methods. A ME-model named `me_model` can be saved follows.

```
In [ ]: import pickle
        with open('[save_loc]/model.pickle', 'wb') as f:
            pickle.dump(me_model, f)
```

It can then be loaded with:

```
In [ ]: with open('[save_loc]/model.pickle', 'wb') as f:
            new_me_model = pickle.load(f)
```

This is not a recommended way to save a ME-model when sharing or for use over the long term as it can break when using different software versions.

CHAPTER 6

Coupling Constraint Derivations

This section will show in detail how coupling coefficients for two macromolecules (**mRNA** and **ribosome**) are derived. The remaining macromolecule coupling derivations follow a similar approach and logic, therefore they are omitted here. For remaining derivations, reference O'Brien et al, 2013.

6.1 Parameters

The parameters for the mRNA coupling coefficient derivations are listed below:

$$\mathrm{P} = \text{total cellular protein mass fraction } (\frac{\mathrm{g_{aa}}}{\mathrm{gDW_{cell}}})$$

$$R = total\ cellular\ RNA\ mass\ fraction\ (\frac{g_{nt}}{gDW_{cell}})$$

$$\mu = specific\ growth\ rate\ (\frac{1}{hr})$$

$$f_{rRNA} = mass\ fraction\ of\ RNA\ that\ is\ rRNA(\frac{g_{nt}}{g_{nt_{total}}})$$

$$f_{tRNA} = mass\ fraction\ of\ RNA\ that\ is\ tRNA(\frac{g_{nt}}{g_{nt_{total}}})$$

$$f_{mRNA} = mass\ fraction\ of\ RNA\ that\ is\ mRNA(\frac{g_{nt}}{g_{nt_{total}}})$$

$$m_{aa} = molecular\ weight\ of\ average\ amino\ acid(\frac{g_{aa}}{mol_{aa}})$$

$$m_{nt} = molecular\ weight\ of\ average\ mRNA\ nucleotide(\frac{g_{nt}}{mol_{nt}})$$

$$m_{tRNA} = molecular\ weight\ of\ average\ tRNA(\frac{g_{tRNA}}{mol_{tRNA}})$$

$$m_{rr} = mass\ of\ rRNA\ per\ ribosome(\frac{g_{nt}}{mol_{ribosome}})$$

$$k_{deg}^{mRNA} = first - order\ mRNA\ degradation\ constant(\frac{1}{hr})$$

Along with an experical relationship between measured ratio of RNA (R) to Protein (P)

$$\frac{\mathrm{R}}{\mathrm{P}} = \frac{\mu}{\kappa_\tau} + \mathrm{r}_0 = \frac{\mu + \kappa_\tau \cdot \mathrm{r}_0}{\kappa_\tau}$$

For E. coli grown at $37^o C$, (Scott et al., 2010) emperically found $r_0 = 0.087$ and $\kappa_\tau = 4.5 \frac{1}{hr}$.

6.2 Derivation of mRNA coupling coefficients

To derive the mRNA dilution and degradation coupling coefficients, we assume that these processes are coupled together as follows.

$$\mathrm{v}_{\mathrm{dilution}_{\mathrm{nt}_{\mathrm{mRNA}}}} = \alpha_1 \cdot \mathrm{v}_{\mathrm{degradation}_{\mathrm{nt}_{\mathrm{mRNA}}}}$$
$$\mathrm{v}_{\mathrm{degradation}_{\mathrm{nt}_{\mathrm{mRNA}}}} = \alpha_2 \cdot \mathrm{v}_{\mathrm{translation}_{\mathrm{aa}_{\mathrm{protein}}}}$$

where α_1 and α_2 represent the coupling of degradation to dilution and translation to degredation, respectively. For the remainder of the mRNA coupling derivation we will abbreviate these reaction rates as $\mathrm{v}_{\mathrm{dilution}}, \mathrm{v}_{\mathrm{degradation}} \text{and } \mathrm{v}_{\mathrm{translation}}$ for simplicity.

To find these coupling values, we will need to find $\mathrm{v}_{\mathrm{dilution}}, \mathrm{v}_{\mathrm{degradation}} \text{and } \mathrm{v}_{\mathrm{translation}}$. The dilution of mRNA nucleotides as it is passed on to daughter cells is related to the concentration of mRNA nucleotides and the growth rate as follows:

$$\mathrm{v}_{\mathrm{dilution}} = \mu \cdot [\mathrm{nt}_{\mathrm{mRNA}}]$$

similarly the degradation rate can be found using the first order rate constant of mRNA degradation

$$\mathrm{v}_{\mathrm{degradation}} = \mathrm{k}_{\mathrm{deg}}^{\mathrm{mRNA}} \cdot [\mathrm{nt}_{\mathrm{mRNA}}]$$

the rate of translation / protein synthesis rate in ($\frac{mol_{aa}}{hr}$) can be found using the following. This represents the rate which amino acid are incorporated into protein:

$$\mathrm{v}_{\mathrm{translation}} = \frac{\mu \cdot \mathrm{P}}{\mathrm{m}_{\mathrm{aa}}}$$

The concentration of mRNA nucleotides in units of ($\frac{mol_{nt}}{gDW_{cell}}$) can be defined as:

$$[\mathrm{nt}_{\mathrm{mRNA}}] = \frac{\mathrm{R} \cdot \mathrm{f}_{\mathrm{mRNA}}}{\mathrm{m}_{\mathrm{nt}}}$$

6.2.1 Solving for mRNA coupling coefficients

Solving for each of these coupling terms gives:

$$\boxed{\alpha_1 = \frac{\mathrm{v}_{\mathrm{dilution}}}{\mathrm{v}_{\mathrm{degradation}}} = \frac{\mu \cdot [\mathrm{nt}_{\mathrm{mRNA}}]}{\mathrm{k}_{\mathrm{deg}}^{\mathrm{mRNA}} \cdot [\mathrm{nt}_{\mathrm{mRNA}}]} = \frac{\mu}{\mathrm{k}_{\mathrm{deg}}^{\mathrm{mRNA}}}}$$
$$\alpha_2 = \frac{\mathrm{v}_{\mathrm{degradation}}}{\mathrm{v}_{\mathrm{translation}}} = \frac{\mathrm{k}_{\mathrm{deg}}^{\mathrm{mRNA}} \cdot [\mathrm{nt}_{\mathrm{mRNA}}]}{\frac{\mu \cdot \mathrm{P}}{\mathrm{m}_{\mathrm{aa}}}}$$

substituting for [mRNA] gives:

$$\alpha_2 = \frac{k_{deg}^{mRNA} \cdot \frac{R \cdot f_{mRNA}}{m_{nt}}}{\frac{\mu \cdot P}{m_{aa}}} = \frac{k_{deg}^{mRNA} \cdot R \cdot f_{mRNA} \cdot m_{aa}}{m_{nt} \cdot \mu \cdot P}$$

$$simplifying:$$

$$\alpha_2 = \frac{k_{deg}^{mRNA}}{\mu} \cdot \frac{R}{P} \cdot \frac{f_{mRNA} \cdot m_{aa}}{m_{nt}}$$

substitution for $\frac{R}{P}$ gives:

$$\boxed{\alpha_2 = \frac{k_{deg}^{mRNA}}{\mu} \cdot \frac{\mu + \kappa_\tau \cdot r_0}{\kappa_\tau} \cdot \frac{f_{mRNA} \cdot m_{aa}}{m_{nt}}}$$

Simplifying the above relationship, the coupling of dilution to translation is represented by:

$$v_{dilution} = \alpha_1 \cdot \alpha_2 \cdot v_{translation}$$

$$where:$$

$$\boxed{\alpha_1 \cdot \alpha_2 = \frac{\mu + \kappa_\tau \cdot r_0}{\kappa_\tau} \cdot \frac{f_{mRNA} \cdot m_{aa}}{m_{nt}}}$$

Therefore $\frac{\mu}{k_{mRNA}} = \alpha_1 \cdot \alpha_2$ and:

$$k_{mRNA} = \frac{\mu}{\alpha_1 \cdot \alpha_2} = \frac{\mu \cdot \kappa_\tau}{\mu + \kappa_\tau \cdot r_0} \cdot \frac{m_{nt}}{f_{mRNA} \cdot m_{aa}}$$

6.2.2 Units of mRNA coupling

Based on the $[nt_{mRNA}]$ expression above, the units will be:

$$[nt_{mRNA}] = \frac{R \cdot f_{mRNA}}{m_{nt}} \xrightarrow{units} \frac{(\frac{g_{nt_{total}}}{gDW_{cell}}) \cdot (\frac{g_{nt}}{g_{nt_{total}}})}{(\frac{g_{nt}}{mol_{nt}})} = (\frac{mol_{nt}}{gDW_{cell}})$$

therefore $v_{degradation}$ will be :

$$v_{degradation} = k_{deg}^{mRNA} \cdot [nt_{mRNA}] \xrightarrow{units} (\frac{1}{hr}) \cdot (\frac{mol_{nt}}{gDW_{cell}}) = (\frac{mol_{nt}}{gDW_{cell} \cdot hr})$$

and for $v_{translation}$:

$$v_{translation} = \frac{\mu \cdot P}{m_{aa}} \xrightarrow{units} \frac{(\frac{1}{hr}) \cdot (\frac{g_{aa}}{gDW_{cell}})}{(\frac{g_{aa}}{mol_{aa}})} = (\frac{mol_{aa}}{gDW_{cell} \cdot hr})$$

and for $v_{dilution}$:

$$v_{dilution} = \mu \cdot [nt_{mRNA}] \xrightarrow{units} (\frac{1}{hr}) \cdot (\frac{mol_{nt}}{gDW_{cell}}) = (\frac{mol_{nt}}{gDW_{cell} \cdot hr})$$

6.2.3 Applying mRNA coupling to translation

Note that the units for each reaction detailed in the above derivations describe the overall coupling of translation, dilution, and degradation cell-wide. For individual proteins and ME-model translation reactions, we will have:

$$v_{dilution_i} = \alpha_1 \cdot \alpha_2 \cdot \frac{len_{peptide_i}}{len_{mRNA_i}} \cdot v_{translation_i}$$

the length terms are required due to the fact that $\mathrm{v_{dilution_i}}$ and $\mathrm{v_{translation_i}}$ will have units of $\frac{\mathrm{mol_{mRNA_i}}}{\mathrm{gDW \cdot hr}}$ and $\frac{\mathrm{mol_{protein_i}}}{\mathrm{gDW \cdot hr}}$, respectively.

Since:

$$\alpha_1 \cdot \alpha_2 = \frac{\mathrm{v_{dilution}}}{\mathrm{v_{translation}}} \xrightarrow{\text{units}} \frac{\mathrm{mol_{nt}}}{\mathrm{mol_{aa}}}$$

therefore:

$$\alpha_1 \cdot \alpha_2 \cdot \frac{\mathrm{len_{peptide_i}}}{\mathrm{len_{mRNA_i}}} \xrightarrow{\text{units}} (\frac{\mathrm{mol_{nt}}}{\mathrm{mol_{aa}}}) \cdot (\frac{\frac{\mathrm{mol_{aa}}}{\mathrm{mol_{peptide_i}}}}{\frac{\mathrm{mol_{nt}}}{\mathrm{mol_{mRNA_i}}}}) = \frac{\mathrm{mol_{mRNA_i}}}{\mathrm{mol_{protein_i}}}$$

however the length of a peptide will always be 1/3 the length of the mRNA that encodes it (3 nucleotides in a codon) therefore we can replace $(\frac{\mathrm{len_{peptide_i}}}{\mathrm{len_{mRNA_i}}})$ with $(\frac{1}{3}\frac{\mathrm{mol_{aa} \cdot mol_{mRNA_i}}}{\mathrm{mol_{protein_i} \cdot mol_{nt}}})$

therefore the final coupling of dilution to translation will be:

$$\boxed{\mathrm{v_{dilution_i}} = \alpha_1 \cdot \alpha_2 \cdot \frac{1}{3} \cdot \mathrm{v_{translation_i}}}$$

and similarly for degradation coupling:

$$\boxed{\mathrm{v_{degradation_i}} = \alpha_2 \cdot \frac{1}{3} \cdot \mathrm{v_{translation_i}}}$$

6.2.4 Plugging ribosome coupling into a ME-model reaction

The coupling of mRNA synthesis to translation will require considering the sum of the mRNA dilution and degration. When imposed in the ME-model, a translation reaction will look similar to following:

$$\mathrm{x \cdot charged\_tRNAs} + (\frac{1}{3} \cdot \alpha_1 \cdot \alpha_2 + \frac{1}{3} \cdot \alpha_2) \cdot \mathrm{mRNA_i} + \mathrm{y \cdot ribosome} \xrightarrow{\mathrm{v_{translation_i}}} \mathrm{protein_i} + \frac{1}{3} \cdot \alpha_2 \cdot \mathrm{nucleotides}$$

with the coupling coefficients substituted:

$$\mathrm{x \cdot charged\_tRNAs} + (\frac{1}{3} \cdot \frac{\mu + \kappa_\tau \cdot \mathrm{r_0}}{\kappa_\tau} \cdot \frac{\mathrm{f_{mRNA} \cdot m_{aa}}}{\mathrm{m_{nt}}} + \frac{1}{3} \cdot \frac{k_{deg}^{mRNA}}{\mu} \cdot \frac{\mu + \kappa_\tau \cdot r_0}{\kappa_\tau} \cdot \frac{f_{mRNA} \cdot m_{aa}}{m_{nt}}) \cdot mRNA_i + y \cdot ribosome \xrightarrow{v_{translation_i}} protein_i + (\frac{1}{3} \cdot \frac{k_{deg}^{mRNA}}{\mu} \cdot \frac{\mu + \kappa_\tau \cdot r_0}{\kappa_\tau} \cdot \frac{f_{mRNA} \cdot m_{aa}}{m_{nt}}) \cdot nucleotides$$

where x and y represents the coupling coefficient for the tRNAs and ribosome (the ribosome coupling is derived below). The reaction will produce nucleotides with a coefficient of $\frac{1}{3} \cdot \alpha_2$ since these are the product of mRNA degradation.

Note: There is a minor typo in the O'brien et al., 2013 coupling coefficient derivations where the α_1 and α_2 expresions are multiplied by 3 instead of $\frac{1}{3}$.

6.3 Derivation of ribosome coupling coefficients

Like above, we will derive the coupling between translation and ribosome dilution to daughter cells during cell division. Unlike mRNA, ribosomes and rRNA are stable and we assume they are degraded at a neglible rate

$$\mathrm{v_{dilution_{ribosome}}} = \alpha_3 \cdot \mathrm{v_{translation_{aa_{protein}}}}$$

As for the mRNA coupling derivation above, α_3 represent the coupling of translation to ribosome dilution. For the remainder of the ribosome coupling derivation, we will abbreviate these reaction rates as $\mathrm{v_{dilution}}$and $\mathrm{v_{translation}}$ for simplicity.

The translation of protein is defined as above in the mRNA coupling derivations:

$$\mathrm{v_{translation}} = \frac{\mu \cdot \mathrm{P}}{\mathrm{m_{aa}}}$$

and:

$$\mathrm{v_{dilution}} = \mu \cdot [\mathrm{ribosome}]$$

The concentration of ribosome in units of $(\frac{\mathrm{mol_{ribosome}}}{\mathrm{gDW_{cell}}})$:

$$[\mathrm{ribosome}] = \frac{\mathrm{R} \cdot \mathrm{f_{rRNA}}}{\mathrm{m_{rr}}}$$

plugging in this expression for [ribosome] and solving for α_3 gives :

$$\alpha_3 = \frac{\frac{\mathrm{R} \cdot \mathrm{f_{rRNA}}}{\mathrm{m_{rr}}} \cdot \mu}{\frac{\mu \cdot \mathrm{P}}{\mathrm{m_{aa}}}} = \frac{\mathrm{R}}{\mathrm{P}} \cdot \frac{\mathrm{f_{rRNA}} \cdot \mathrm{m_{aa}}}{\mathrm{m_{rr}}}$$

plugging in the above empirical expression for $\frac{R}{P}$:

$$\boxed{\alpha_3 = \frac{\mu + \kappa_\tau \cdot \mathrm{r_0}}{\kappa_\tau} \cdot \frac{\mathrm{f_{rRNA}} \cdot \mathrm{m_{aa}}}{\mathrm{m_{rr}}}}$$

6.3.1 Units of ribosome coupling

$$\mathrm{v_{dilution}} = \mu \cdot [\mathrm{ribosome}] \xrightarrow{\mathrm{units}} (\frac{1}{\mathrm{hr}}) \cdot \frac{(\frac{\mathrm{g_{nt_{total}}}}{\mathrm{gDW}}) \cdot (\frac{\mathrm{g_{nt_{ribosome}}}}{\mathrm{g_{nt_{total}}}})}{(\frac{\mathrm{g_{nt_{ribosome}}}}{\mathrm{mol_{ribosome}}})} = (\frac{\mathrm{mol_{ribosome}}}{\mathrm{gDW_{cell}} \cdot \mathrm{hr}})$$

$$v_{translation} = \frac{\mu \cdot P}{m_{aa}} \xrightarrow{units} = \frac{(\frac{1}{hr}) \cdot (\frac{g_{aa}}{gDW_{cell}})}{(\frac{g_{aa}}{mol_{aa}})} = (\frac{mol_{aa}}{gDW_{cell} \cdot hr})$$

6.3.2 Applying ribosome coupling to translation

Note that the units for each reaction detailed in the above derivations describe the overall coupling of translation to ribosome dilution on a cell-wide level. For individual proteins, we will have:

$$\mathrm{v_{dilution_i}} = \alpha_3 \cdot \mathrm{len_{peptide_i}} \cdot \mathrm{v_{translation_i}}$$

The length term is required due to the fact that in the ME-model $\mathrm{v_{dilution_i}}$ and $\mathrm{v_{translation_i}}$ will have units of $\frac{\mathrm{mol_{ribosome}}}{\mathrm{gDW_{cell} \cdot hr}}$ and $\frac{\mathrm{mol_{protein_i}}}{\mathrm{gDW_{cell} \cdot hr}}$, respectively.

Since:

$$\alpha_3 = \frac{\mathrm{v_{dilution}}}{\mathrm{v_{translation}}} \xrightarrow{\text{units}} \frac{\mathrm{mol_{ribosome}}}{\mathrm{mol_{aa}}}$$

therefore:

$$(\alpha_3) \cdot (\mathrm{len_{peptide_i}}) \xrightarrow{\text{units}} (\frac{\mathrm{mol_{ribosome}}}{\mathrm{mol_{aa}}}) \cdot (\frac{\mathrm{mol_{aa}}}{\mathrm{mol_{peptide_i}}}) = \frac{\mathrm{mol_{ribosome}}}{\mathrm{mol_{protein_i}}}$$

therefore plugging this into the final coupling of dilution to translation will be:

$$\boxed{\mathrm{v_{dilution_i}} = \alpha_3 \cdot \mathrm{len_{protein_i}} \cdot \mathrm{v_{translation_i}}}$$

confirming units:

$$\mathrm{v_{dilution_i}} = \alpha_3 \cdot \mathrm{len_{protein_i}} \cdot \mathrm{v_{translation_i}} \xrightarrow{\text{units}} (\frac{\mathrm{mol_{ribosome}}}{\mathrm{mol_{protein_i}}}) \cdot (\frac{\mathrm{mol_{protein_i}}}{\mathrm{gDW_{cell}}}) = \frac{\mathrm{mol_{ribosome}}}{\mathrm{gDW_{cell}}}$$

6.3.3 Applying ribosome coupling to translation

When further imposing ribosome dilution coupling in the ME-model, a translation reaction will look similar to following:

$$\mathrm{x} \cdot \mathrm{charged\_tRNAs} + (\frac{1}{3} \cdot \alpha_1 \cdot \alpha_2 + \frac{1}{3} \cdot \alpha_2) \cdot \mathrm{mRNA_i} + \mathrm{len_{protein_i}} \cdot \alpha_3 \cdot \mathrm{ribosome} \xrightarrow{\mathrm{v_{translation_i}}} \mathrm{protein_i} + \frac{1}{3} \cdot \alpha_2 \cdot \mathrm{nucleotides}$$

with the coupling coefficients substituted:

$$\mathrm{x} \cdot \mathrm{charged\_tRNAs} + (\frac{1}{3} \cdot \frac{\mu + \kappa_\tau \cdot \mathrm{r_0}}{\kappa_\tau} \cdot \frac{\mathrm{f_{mRNA}} \cdot \mathrm{m_{aa}}}{\mathrm{m_{nt}}} + \frac{1}{3} \cdot \frac{k_{deg}^{mRNA}}{\mu} \cdot \frac{\mu + \kappa_\tau \cdot r_0}{\kappa_\tau} \cdot \frac{f_{mRNA} \cdot m_{aa}}{m_{nt}}) \cdot mRNA_i + (len_{protein} \cdot \frac{\mu + \kappa_\tau \cdot r_0}{\kappa_\tau} \cdot \frac{f_{rRNA} \cdot m_{aa}}{m_{rr}}) \cdot ribosome \xrightarrow{v_{translation_i}} protein_i + (\frac{1}{3} \cdot \frac{k_{deg}^{mRNA}}{\mu} \cdot \frac{\mu + \kappa_\tau \cdot r_0}{\kappa_\tau} \cdot \frac{f_{mRNA} \cdot m_{aa}}{m_{nt}}) \cdot nucleotides$$

where x represents the coupling coefficient for the tRNAs.

CHAPTER 7

cobrame package

7.1 Subpackages

7.1.1 cobrame.core package

Submodules

cobrame.core.reaction module

class `cobrame.core.reaction.`**`ComplexFormation`**(*id*)

Bases: *`cobrame.core.reaction.MEReaction`*

Formation of a functioning enzyme complex that can act as a catalyst for a ME-model reaction.

This reaction class produces a reaction that combines the protein subunits and adds any coenyzmes, prosthetic groups or enzyme modifications to form complete enzyme complex.

Parameters **`id`** (*`str`*) – Identifier of the complex formation reaction. As a best practice, this ID should be prefixed with 'formation + _ + <complex_id>'. If there are multiple ways of producing complex, this can be suffixed with '_ + alt'

`_complex_id`

str – Name of the complex being produced by the complex formation reaction

`complex_data_id`

str – Name of ComplexData that defines the subunit stoichiometry or subreactions (modfications). This will not always be the same as the _complex_id. Sometimes complexes can be modified using different processes/enzymes

`complex`

Get the metabolite product of the complex formation reaction

Returns Instance of complex metabolite from self._complex_id

Return type *`cobrame.core.component.Complex`*

update (*verbose=True*)

Creates reaction using the associated complex data and adds chemical formula to complex metabolite product.

This function adds the following components to the reaction stoichiometry (using 'data' as shorthand for *cobrame.core.processdata.ComplexData*):

1. Complex product defined in self._complex_id
2. Protein subunits with stoichiometery defined in data.stoichiometry
3. Metabolites and enzymes w/ coupling coefficients defined in data.subreactions. This often includes enzyme complex modifications by coenzymes or prosthetic groups.
4. Biomass *cobrame.core.component.Constraint* corresponding to modifications detailed in data.subreactions, if any

Parameters **verbose** (*bool*) – Prints when new metabolites are added to the model when executing update()

class cobrame.core.reaction.**GenericFormationReaction** (*id*)

Bases: *cobrame.core.reaction.MEReaction*

Some components in an ME-model can perform exactly the same function. To handle this, GenericFormation-Reactions are used to create generic forms of these components.

Parameters **id** (*str*) – Identifier of the generic formation reaction. As a best practice, this ID should be prefixed with 'metabolite_id + _to_ + generic_metabolite_id'

class cobrame.core.reaction.**MEReaction** (*id=None*, *name=''*)

Bases: cobra.core.Reaction.Reaction

MEReaction is a general reaction class from which all ME-Model reactions will inherit

This class contains functionality that can be used by all ME-model reactions

Parameters **id** (*str*) – Identifier of the MEReaction. Should follow best practices of child class

add_biomass_from_subreactions (*process_data*, *biomass=0.0*)

Account for the biomass of metabolites added to macromolecule (protein, complex, etc.) due to a modification such as prosthetic group addition.

Parameters

- **process_data** (*cobrame.core.processdata.ProcessData*) – ProcessData that is used to construct MEReaction
- **biomass** (*float*) – Initial biomass value in kDa

Returns Initial biomass value + biomass added from subreactions in kDa

Return type float

add_subreactions (*process_data_id*, *stoichiometry*, *scale=1.0*)

Function to add subreaction process data to reaction stoichiometry

Parameters

- **process_data_id** (*str*) – ID of the process data associated with the metabolic reaction.

  For example, if the modifications are being added to a complex formation reaction, the process data id would be the name of the complex.

- **stoichiometry** (dict) – Dictionary of {metabolite_id: float} or {metabolite_id: float * (sympy.Symbol)}
- **scale** (*float*) – Some processes (ie. tRNA charging) are reformulated such that other involved metabolites need scaling

Returns Stoichiometry dictionary with updated entries

Return type *dict*

check_me_mass_balance()

Checks the mass balance of ME reaction, ignoring charge balances

Returns {element: number_of_elemental_imbalances}

Return type *dict*

clear_metabolites()

Remove all metabolites from the reaction

get_components_from_ids(*id_stoichiometry*, *default_type=<class 'cobrame.core.component.Metabolite'>*, *verbose=True*)

Function to convert stoichiometry dictionary entries from strings to cobra objects.

{metabolite_id: value} to {*cobrame.core.component.Metabolite*: value}

Parameters

- **id_stoichiometry** (*Dict {string: float}*) – Input Dict of {metabolite_id: value}
- **default_type** (*String*) – The type of cobra.Metabolite to default to if the metabolite is not yet present in the model
- **verbose** (*Boolean*) – If True, print metabolites added to model if not yet present in model

Returns {*cobrame.core.component.Metabolite*: float}

Return type *dict*

objective_coefficient

Get and set objective coefficient of reaction

Overrides method in parent class in order to enable use of optlang interfaces.

Returns Objective coefficient of reaction

Return type float

class cobrame.core.reaction.**MetabolicReaction**(*id*)

Bases: *cobrame.core.reaction.MEReaction*

Irreversible metabolic reaction including required enzymatic complex

This reaction class's update function processes the information contained in the complex data for the enzyme that catalyzes this reaction as well as the stoichiometric data which contains the stoichiometry of the metabolic conversion being performed (i.e. the stoichiometry of the M-model reaction analog)

Parameters **id** (*str*) – Identifier of the metabolic reaction. As a best practice, this ID should use the following template (FWD=forward, REV=reverse): "<StoichiometricData.id> + _ + <FWD or REV> + _ + <Complex.id>"

keff

float – The turnover rete (keff) couples enzymatic dilution to metabolic flux

reverse
boolean – If True, the reaction corresponds to the reverse direction of the reaction. This is necessary since all reversible enzymatic reactions in an ME-model are broken into two irreversible reactions

complex_data
Get or set the ComplexData instance that details the enzyme that catalyzes the metabolic reaction. Can be set with instance of ComplexData or with its id.

Returns Complex data detailing enzyme that catalyzes this reaction

Return type *cobrame.core.processdata.ComplexData*

stoichiometric_data
Get or set the StoichiometricData instance that details the metabolic conversion of the metabolic reaction. Can be set with instance of StoichiometricData or with its id.

Returns Stoichiometric data detailing enzyme that catalyzes this reaction

Return type :class:‘cobrame.core.processdata.StoichiometricData ‘

update(*verbose=True*)
Creates reaction using the associated stoichiometric data and complex data.

This function adds the following components to the reaction stoichiometry (using ‘data’ as shorthand for *cobrame.core.processdata.StoichiometricData*):

1. Complex w/ coupling coefficients defined in self.complex_data.id and self.keff
2. Metabolite stoichiometry defined in data.stoichiometry. Sign is flipped if self.reverse == True

Also sets the lower and upper bounds based on self.reverse and data.upper_bound and data.lower_bound.

Parameters verbose (*bool*) – Prints when new metabolites are added to the model when executing update()

class cobrame.core.reaction.**PostTranslationReaction**(*id*)
Bases: *cobrame.core.reaction.MEReaction*

Reaction class that includes all posttranslational modification reactions (translocation, protein folding, modification (for lipoproteins) etc)

There are often multiple different reactions/enzymes that can accomplish the same modification/function. In order to account for these and maintain one translation reaction per protein, these processes need to be modeled as separate reactions.

Parameters id (*str*) – Identifier of the post translation reaction

add_translocation_pathways(*process_data_id, protein_id, stoichiometry=None*)
Add complexes and metabolites required to translocate the protein into cell membranes.

Parameters

- **process_data_id** (*str*) – ID of translocation data defining post translation reaction
- **protein_id** (*str*) – ID of protein being translocated via post translation reaction
- **stoichiometry** (dict) – Dictionary of {metabolite_id: float} or {metabolite_id: float * (sympy.Symbol)}

Returns Stoichiometry dictionary with updated entries from translocation

Return type *dict*

posttranslation_data
Get or set PostTranslationData that defines the type of post translation modification/process (folding/translocation) that the reaction accounts for. Can be set with instance of PostTranslationData or with its id.

Returns The PostTranslationData that defines the PostTranslationReaction

Return type *cobrame.core.processdata.PostTranslationData*

update(*verbose=True*)
Creates reaction using the associated posttranslation data and adds chemical formula to processed protein product

This function adds the following components to the reaction stoichiometry (using 'data' as shorthand for *cobrame.core.processdata.PostTranslationData*):

1. Processed protein product defined in data.processed_protein_id
2. Unprocessed protein reactant defined in data.unprocessed_protein_id
3. Metabolites and enzymes defined in data.subreactions
4. Translocation pathways defined in data.translocation
5. Folding mechanism defined in data.folding_mechanims w/ coupling coefficients defined in data.keq_folding, data.k_folding, model.global_info['temperature'], data.aggregation_propensity, and data.propensity_scaling
6. Surface area constraints defined in data.surface_are
7. Biomass if a significant chemical modification takes place (i.e. lipid modifications for lipoproteins)

Parameters verbose (*bool*) – Prints when new metabolites are added to the model when executing update()

class cobrame.core.reaction.**SummaryVariable**(*id=None*)
Bases: *cobrame.core.reaction.MEReaction*

SummaryVariables are reactions that impose global constraints on the model.

The primary example of this is the biomass_dilution SummaryVariable which forces the rate of biomass production of macromolecules, etc. to be equal to the rate of their dilution to daughter cells during growth.

Parameters id (*str*) – Identifier of the SummaryVariable

class cobrame.core.reaction.**TranscriptionReaction**(*id*)
Bases: *cobrame.core.reaction.MEReaction*

Transcription of a TU to produced TranscribedGene.

RNA is transcribed on a transcription unit (TU) level. This type of reaction produces all of the RNAs contained within a TU, as well as accounts for the splicing/excision of RNA between tRNAs and rRNAs. The appropriate RNA_biomass constrain is produced based on the molecular weight of the RNAs being transcribed

Parameters id (*str*) – Identifier of the transcription reaction. As a best practice, this ID should be prefixed with 'transcription + _'

transcription_data
Get or set the *cobrame.core.processdata.TranscriptionData* that defines the transcription unit architecture and the features of the RNAs being transcribed.

update(*verbose=True*)
Creates reaction using the associated transcription data and adds chemical formula to RNA products

This function adds the following components to the reaction stoichiometry (using 'data' as shorthand for *cobrame.core.processdata.TranscriptionData*):

1. RNA_polymerase from data.RNA_polymerase w/ coupling coefficient (if present)
2. RNA products defined in data.RNA_products
3. Nucleotide reactants defined in data.nucleotide_counts
4. If tRNA or rRNA contained in data.RNA_types, excised base products
5. Metabolites + enzymes w/ coupling coefficients defined in data.subreactions (if present)
6. Biomass *cobrame.core.component.Constraint* corresponding to data.RNA_products and their associated masses
7. Demand reactions for each transcript product of this reaction

Parameters verbose (*bool*) – Prints when new metabolites are added to the model when executing update()

class cobrame.core.reaction.**TranslationReaction**(*id*)

Bases: *cobrame.core.reaction.MEReaction*

Reaction class for the translation of a TranscribedGene to a TranslatedGene

Parameters id (*str*) – Identifier of the translation reaction. As a best practice, this ID should be prefixed with 'translation + _'

translation_data

Get and set the cobra.core.processdata.TranslationData that defines the translation of the gene. Can be set with instance of TranslationData or with its id.

Returns

Return type cobra.core.processdata.TranslationData

update(*verbose=True*)

Creates reaction using the associated translation data and adds chemical formula to protein product

This function adds the following components to the reaction stoichiometry (using 'data' as shorthand for *cobrame.core.processdata.TranslationData*):

1. Amino acids defined in data.amino_acid_sequence. Subtracting water to account for condensation reactions during polymerization
2. Ribosome w/ translation coupling coefficient (if present)
3. mRNA defined in data.mRNA w/ translation coupling coefficient
4. mRNA + nucleotides + hydrolysis ATP cost w/ degradation coupling coefficient (if kdeg (defined in model.global_info) > 0)
5. RNA_degradosome w/ degradation coupling coefficient (if present and kdeg > 0)
6. Protein product defined in data.protein
7. Subreactions defined in data.subreactions
8. protein_biomass *cobrame.core.component.Constraint* corresponding to the protein product's mass
9. Subtract mRNA_biomass *cobrame.core.component.Constraint* defined by mRNA degradation coupling coefficinet (if kdeg > 0)

Parameters **verbose** (*bool*) – Prints when new metabolites are added to the model when executing update()

class cobrame.core.reaction.**tRNAChargingReaction**(*id*)
Bases: *cobrame.core.reaction.MEReaction*

Reaction class for the charging of a tRNA with an amino acid

Parameters **id** (*str*) – Identifier for the charging reaction. As a best practice, ID should follow the template "charging_tRNA + _ + <tRNA_locus> + _ + <codon>". If tRNA initiates translation, <codon> should be replaced with START.

tRNA_data
Get and set the cobra.core.processdata.tRNAData that defines the translation of the gene. Can be set with instance of tRNAData or with its id.

Returns

Return type cobra.core.processdata.tRNAData

update(*verbose=True*)
Creates reaction using the associated tRNA data

This function adds the following components to the reaction stoichiometry (using 'data' as shorthand for *cobrame.core.processdata.tRNAData*):

1. Charged tRNA product following template: "generic_tRNA + _ + <data.codon> + _ + <data.amino_acid>"
2. tRNA metabolite (defined in data.RNA) w/ charging coupling coefficient
3. Charged amino acid (defined in data.amino_acid) w/ charging coupling coefficient

5. Synthetase (defined in data.synthetase) w/ synthetase coupling coefficient found, in part, using data.synthetase_keff
6. Post transcriptional modifications defined in data.subreactions

Parameters **verbose** (*bool*) – Prints when new metabolites are added to the model when executing update()

cobrame.core.processdata module

class cobrame.core.processdata.**ComplexData**(*id*, *model*)
Bases: *cobrame.core.processdata.ProcessData*

Contains all information associated with the formation of an functional enzyme complex.

This can include any enzyme complex modifications required for the enzyme to become active.

Parameters

- **id** (*str*) – Identifier of the complex data. As a best practice, this should typically use the same ID as the complex being formed. In cases with multiple ways to form complex '_ + alt' or similar suffixes can be used.
- **model** (*cobrame.core.model.MEModel*) – ME-model that the ComplexData is associated with

stoichiometry
collections.DefaultDict(int) – Dictionary containing {protein_id: count} for all protein subunits comprising enzyme complex

subreactions
dict – Dictionary of {subreaction_data_id: count} for all complex formation subreactions/modifications. This can include cofactor/prosthetic group binding or enzyme side group addition.

complex
Get complex metabolite object

Returns Instance of complex metabolite that ComplexData is used to synthesize

Return type *cobrame.core.component.Complex*

complex_id
Get and set complex ID for product of complex formation reaction

There are cases where multiple equivalent processes can result in the same final complex. This allows the equivalent final complex complex_id to be queried. This only needs set in the above case

Returns ID of complex that ComplexData is used to synthesize

Return type str

create_complex_formation(*verbose=True*)
creates a complex formation reaction

This assumes none exists already. Will create a reaction (prefixed by "formation") which forms the complex

Parameters **verbose** (*bool*) – If True, print if a metabolite is added to model during update

formation
Get the formation reaction object

Returns Complex formation reaction detailed in ComplexData

Return type *cobrame.core.reaction.ComplexFormation*

class cobrame.core.processdata.**GenericData**(*id*, *model*, *component_list*)
Bases: *cobrame.core.processdata.ProcessData*

Class for storing information about generic metabolites

Parameters

- **id** (*str*) – Identifier of the generic metabolite. As a best practice, this ID should be prefixed with 'generic + _'
- **model** (*cobrame.core.model.MEModel*) – ME-model that the GenericData is associated with
- **component_list** (*list*) – List of metabolite ids for all metabolites that can provide identical functionality

create_reactions()
Adds reaction with id "<metabolite_id> + _ + to + _ + <generic_id>" for each metabolite in self.component_list.

Creates generic metabolite and generic reaction, if they do not already exist.

class cobrame.core.processdata.**PostTranslationData**(*id*, *model*, *processed_protein*, *preprocessed_protein*)
Bases: *cobrame.core.processdata.ProcessData*

Parameters

- **id** (*str*) – Identifier for post translation process.

- **model** (*cobrame.core.model.MEModel*) – ME-model that the PostTranslationData is associated with
- **processed_protein** (*str*) – ID of protein following post translational process
- **preprocessed_protein** (*str*) – ID of protein before post translational process

translocation
set – Translocation pathways involved in post translation reaction.

Set of {`cobrame.core.processdata.TranslocationData.id`}

translocation_multipliers
dict – Some proteins require different coupling of translocation enzymes.

Dictionary of {`cobrame.core.processdata.TranslocationData.id`: float}

surface_area
dict – If protein is translated into the inner or outer membrane, the surface area the protein occupies can be accounted for as well.

Dictionary of {SA_+<inner_membrane or outer_membrane>: float}

subreactions
`collections.DefaultDict(float)` – If a protein is modified following translation, this is accounted for here

Dictionary of {subreaction_id: float}

biomass_type
str – If the subreactions add biomass to the translated gene, the biomass type (`cobrame.core.compontent.Constraint.id`) of the modification must be defined.

folding_mechanism
str – ID of folding mechanism for post translation reaction

aggregation_propensity
float – Aggregation propensity for the protein

keq_folding
dict – Temperature dependant keq for folding protein

Dictionary of {str(temperature): value}

k_folding
dict – Temperature dependant rate constant (k) for folding protein

Dictionary of {str(temperature): value}

propensity_scaling
float – Some small peptides are more likely to be folded by certain chaperones. This is accounted for using propensity_scaling.

class `cobrame.core.processdata.`**ProcessData**(*id*, *model*)
Bases: `object`

Generic class for storing information about a process

This class essentially acts as a database that contains all of the relevant information needed to construct a particular reaction. For example, to construct a transcription reaction, following information must be accessed in some way:

- nucleotide sequence of the transcription unit
- RNA_polymerase (w/ sigma factor)

- RNAs transcribed from transcription unit
- other processes involved in transcription of RNAs (splicing, etc.)

ME-model reactions are built from information in these objects.

Parameters

- **id** (*str*) – Identifier of the ProcessData instance.
- **model** (*cobrame.core.model.MEModel*) – ME-model that the ProcessData is associated with

model

Get the ME-model the process data is associated with

Returns ME-model that uses this process data

Return type class:'cobrame.core.model.MEModel

parent_reactions

Get reactions that the ProcessData instance is used to construct.

Returns Parent reactions of ProcessData

Return type set

update_parent_reactions()

Executes the update() function for all reactions that the ProcessData instance is used to construct.

class cobrame.core.processdata.**StoichiometricData**(*id*, *model*)

Bases: *cobrame.core.processdata.ProcessData*

Encodes the stoichiometry for a metabolic reaction.

StoichiometricData defines the metabolite stoichiometry and upper/lower bounds of metabolic reaction

Parameters

- **id** (*str*) – Identifier of the metabolic reaction. Should be identical to the M-model reactions in most cases.
- **model** (*cobrame.core.model.MEModel*) – ME-model that the StoichiometricData is associated with

_stoichiometry

dict – Dictionary of {metabolite_id: stoichiometry} for reaction

subreactions

collections.DefaultDict(int) – Cases where multiple enzymes (often carriers ie. Acyl Carrier Protein) are involved in a metabolic reactions.

upper_bound

int – Upper reaction bound of metabolic reaction. Should be identical to the M-model reactions in most cases.

lower_bound

int – Lower reaction bound of metabolic reaction. Should be identical to the M-model reactions in most cases.

stoichiometry

Get or set metabolite stoichiometry for reaction.

Returns Dictionary of {metabolite_id: stoichiometry}

Return type *dict*

class `cobrame.core.processdata.`**`SubreactionData`**(*id*, *model*)
Bases: *`cobrame.core.processdata.ProcessData`*

Parameters

- **`id`** (*`str`*) – Identifier of the subreaction data. As a best practice, if the subreaction data details a modification, the ID should be prefixed with "mod + _"
- **`model`** (*`cobrame.core.model.MEModel`*) – ME-model that the SubreactionData is associated with

`enzyme`
list or str or None – List of `cobrame.core.component.Complex.id` s for enzymes that catalyze this process

or

String of single `cobrame.core.component.Complex.id` for enzyme that catalyzes this process

`keff`
float – Effective turnover rate of enzyme(s) in subreaction process

`_element_contribution`
dict – If subreaction adds a chemical moiety to a macromolecules via a modification or other means, net element contribution of the modification process should be accounted for. This can be used to mass balance check each of the individual processes.

Dictionary of {element: net_number_of_contributions}

`calculate_biomass_contribution`()
Calculate net biomass increase/decrease as a result of the subreaction process.

If subreaction adds a chemical moiety to a macromolecules via a modification or other means, the biomass contribution of the modification process should be accounted for and ultimately included in the reaction it is involved in.

Returns Mass of moiety transferred to macromolecule by subreaction

Return type float

`calculate_element_contribution`()
Calculate net contribution of chemical elements based on the stoichiometry of the subreaction data

Returns Dictionary of {element: net_number_of_contributions}

Return type *dict*

`element_contribution`
Get net contribution of elements from subreaction process to macromolecule

If subreaction adds a chemical moiety to a macromolecules via a modification or other means, net element contribution of the modification process should be accounted for. This can be used to mass balance check each of the individual processes.

Returns Dictionary of {element: net_number_of_contributions}

Return type *dict*

`get_all_usages`()
Get all process data that the subreaction is involved in

Yields *`cobrame.core.processdata.ProcessData`* – ProcessData that subreaction is involved in

get_complex_data()
Get the complex data that the subreaction is involved in

Yields *cobrame.core.processdata.ComplexData* – ComplexData that subreaction is involved in

class cobrame.core.processdata.**TranscriptionData**(*id*, *model*, *rna_products=set([])*)
Bases: *cobrame.core.processdata.ProcessData*

Class for storing information needed to define a transcription reaction

Parameters

- **id** (*str*) – Identifier of the transcription unit, typically beginning with 'TU'
- **model** (*cobrame.core.model.MEModel*) – ME-model that the TranscriptionData is associated with

nucleotide_sequence
str – String of base pair abbreviations for nucleotides contained in the transcription unit

RNA_products
set – IDs of *cobrame.core.component.TranscribedGene* that the transcription unit encodes. Each member should be prefixed with "RNA + _"

RNA_polymerase
str – ID of the *cobrame.core.component.RNAP* that transcribes the transcription unit. Different IDs are used for different sigma factors

subreactions
collections.DefaultDict(int) – Dictionary of {*cobrame.core.processdata.SubreactionData* ID: num_usages} required for the transcription unit to be transcribed

RNA_types
Get generator consisting of the RNA type for each RNA product

Yields *str* – (mRNA, tRNA, rRNA, ncRNA)

codes_stable_rna
Get whether transcription unit codes for a stable RNA

Returns True if tRNA or rRNA in RNA products False if not

Return type bool

excised_bases
Get count of bases that are excised during transcription

If a stable RNA (e.g. tRNA or rRNA) is coded for in the transcription unit, the transcript must be spliced in order for these to function.

This determines whether the transcription unit requires splicing and, if so, returns the count of nucleotides within the transcription unit that are not accounted for in the RNA products, thus identifying the appropriate introns nucleotides.

Returns

{nucleotide_monophosphate_id: number_excised}

i.e. {"amp_c": 10, "gmp_c": 11, "ump_c": 9, "cmp_c": 11}

Return type *dict*

nucleotide_count
Get count of each nucleotide contained in the nucleotide sequence

Returns {nuclotide_id: number_of_occurances}

Return type *dict*

class `cobrame.core.processdata.`**`TranslationData`**(*id*, *model*, *mrna*, *protein*)

Bases: `cobrame.core.processdata.ProcessData`

Class for storing information about a translation reaction.

Parameters

- **`id`** (`str`) – Identifier of the gene being translated, typically the locus tag
- **`model`** (`cobrame.core.model.MEModel`) – ME-model that the TranslationData is associated with
- **`mrna`** (`str`) – ID of the mRNA that is being translated
- **`protein`** (`str`) – ID of the protein product.

`mRNA`

str – ID of the mRNA that is being translated

`protein`

str – ID of the protein product.

`subreactions`

`collections.DefaultDict(int)` – Dictionary of {`cobrame.core.processdata.SubreactionData.id`: num_usages} required for the mRNA to be translated

`nucleotide_sequence`

str – String of base pair abbreviations for nucleotides contained in the gene being translated

`add_elongation_subreactions`(*elongation_subreactions=set([])*)

Add all subreactions involved in translation elongation.

This includes:

- tRNA activity subreactions returned with `subreactions_from_sequence()` which is called within this function.
- Elongation subreactions passed into this function. These will be added with a value of len(amino_acid_sequence) - 1 as these are involved in each amino acid addition

Some additional enzymatic processes are required for each amino acid addition during translation elongation

Parameters **`elongation_subreactions`** (`set`) – Subreactions that are required for each amino acid addition

`add_initiation_subreactions`(*start_codons=set([])*, *start_subreactions=set([])*)

Add all subreactions involved in translation initiation.

Parameters

- **`start_codons`** (`set, optional`) – Start codon sequences for the organism being modeled
- **`start_subreactions`** (`set, optional`) – Subreactions required to initiate translation, including the activity by the start tRNA

`add_termination_subreactions`(*translation_terminator_dict=None*)

Add all subreactions involved in translation termination.

Parameters **`translation_terminator_dict`** (`dict or None`) – {stop_codon : enzyme_id_of_terminator_enzyme}

amino_acid_count
Get number of each amino acid in the translated protein

Returns {amino_acid_id: number_of_occurrences}

Return type *dict*

amino_acid_sequence
Get amino acid sequence from mRNA's nucleotide sequence

Returns Amino acid sequence

Return type str

codon_count
Get the number of each codon contained within the gene sequence

Returns {codon_sequence: number_of_occurrences}

Return type *dict*

first_codon
Get the first codon contained in the mRNA sequence. This should correspond to the start codon for the gene.

Returns First 3 nucleotides comprising the first codon in the mRNA gene sequence

Return type str

last_codon
Get the last codon contained in the mRNA sequence. This should correspond to the stop codon for the gene.

Returns Last 3 nucleotides comprising the last codon in the mRNA gene sequence

Return type str

subreactions_from_sequence
Get subreactions associated with each tRNA/AA addition.

tRNA activity is accounted for as subreactions. This returns the subreaction counts associated with each amino acid addition, based on the sequence of the mRNA.

Returns {`cobrame.core.processdata.SubreactionData.id`: num_usages}

Return type *dict*

class `cobrame.core.processdata.`**`TranslocationData`**(*id*, *model*)
Bases: *`cobrame.core.processdata.ProcessData`*

Class for storing information about a protein translocation pathway

Parameters

- **id** (*str*) – Identifier for translocation pathway.
- **model** (*`cobrame.core.model.MEModel`*) – ME-model that the TranslocationData is associated with

keff
float – Effective turnover rate of the enzymes in the translocation pathway

enzyme_dict
dict – Dictionary containing enzyme specific information about the way it is coupled to protein translocation

{enzyme_id: {length_dependent: <True or False>, fixed_keff: <True or False>}}

length_dependent_energy
bool – True if the ATP cost of translocation is dependent on the length of the protein

stoichiometry
dict – Stoichiometry of translocation pathway, typically ATP/GTP hydrolysis

class cobrame.core.processdata.**tRNAData**(*id*, *model*, *amino_acid*, *rna*, *codon*)
Bases: *cobrame.core.processdata.ProcessData*

Class for storing information about a tRNA charging reaction.

Parameters

- **id** (*str*) – Identifier for tRNA charging process. As best practice, this should be follow "tRNA + _ + <tRNA_locus> + _ + <codon>" template. If tRNA initiates translation, <codon> should be replaced with START.
- **model** (*cobrame.core.model.MEModel*) – ME-model that the tRNAData is associated with
- **amino_acid** (*str*) – Amino acid that the tRNA transfers to an peptide
- **rna** (*str*) – ID of the uncharged tRNA metabolite. As a best practice, this ID should be prefixed with 'RNA + _'

subreactions
collections.DefaultDict(int) – Dictionary of {cobrame.core.processdata.SubreactionData.id: num_usages} required for the tRNA to be charged

synthetase
str – ID of the tRNA synthetase required to charge the tRNA with an amino acid

synthetase_keff
float – Effective turnover rate of the tRNA synthetase

cobrame.core.component module

class cobrame.core.component.**Complex**(*id*)
Bases: *cobrame.core.component.MEComponent*

Metabolite class for protein complexes

Parameters **id** (*str*) – Identifier of the protein complex.

metabolic_reactions
Get metabolic reactions catalyzed by complex

Returns List of *cobrame.core.reaction.MetabolicReaction* s catalyzed by complex.

Return type list

class cobrame.core.component.**Constraint**(*id*)
Bases: *cobrame.core.component.MEComponent*

Metabolite class for global constraints such as biomass

Parameters **id** (*str*) – Identifier of the constraint

class cobrame.core.component.**GenericComponent**(*id*)
Bases: *cobrame.core.component.MEComponent*

Metabolite class for generic components created from *cobrame.core.reaction.GenericFormationReaction*

Parameters **id** (*str*) – Identifier of the generic tRNA. As a best practice should follow template: 'generic + _ + <generic metabolite id>'

class cobrame.core.component.**GenerictRNA** (*id*)

Bases: *cobrame.core.component.MEComponent*

Metabolite class for generic tRNAs created from *cobrame.core.reaction.tRNAChargingReaction*

Parameters **id** (*str*) – Identifier of the generic tRNA. As a best practice should follow template: 'generic_tRNA + _ + <codon> + _ + <amino acid metabolite id>'

class cobrame.core.component.**MEComponent** (*id*)

Bases: cobra.core.Metabolite.Metabolite

COBRAme component representation. Inherits from cobra.core.metabolite.Metabolite

Parameters **id** (*str*) – Identifier of the component. Should follow best practices of child classes

remove_from_me_model (*method='subtractive'*)

Remove metabolite from me model along with any relevant *cobrame.core.processdata.ProcessData*

Parameters **method** (*str*) –

- destructive: remove metabolite from model and remove reactions it is involved in
- subtractive: remove only metabolite from model

class cobrame.core.component.**Metabolite** (*id*)

Bases: *cobrame.core.component.MEComponent*

COBRAme metabolite representation

Parameters **id** (*str*) – Identifier of the metabolite

class cobrame.core.component.**ProcessedProtein** (*id*, *unprocessed_protein_id*)

Bases: *cobrame.core.component.MEComponent*

Metabolite class for protein created from *cobrame.core.reaction.PostTranslationReaction*

Parameters

- **id** (*str*) – Identifier of the processed protein
- **unprocessed_protein_id** (*str*) – Identifier of protein before being processed by PostTranslationReaction

unprocessed_protein

Get unprocessed protein reactant in PostTranslationReaction

Returns Unprocessed protein object

Return type *cobrame.core.component.TranslatedGene*

class cobrame.core.component.**RNAP** (*id*)

Bases: *cobrame.core.component.Complex*

Metabolite class for RNA polymerase complexes. Inherits from *cobrame.core.component.Complex*

Parameters **id** (*str*) – Identifier of the RNA Polymerase.

class cobrame.core.component.**Ribosome** (*id*)

Bases: *cobrame.core.component.Complex*

Metabolite class for Ribosome complexes. Inherits from *cobrame.core.component.Complex*

Parameters **id** (*str*) – Identifier of the Ribosome.

class cobrame.core.component.**TranscribedGene** (*id*, *rna_type*, *nucleotide_sequence*)
Bases: *cobrame.core.component.MEComponent*

Metabolite class for gene created from *cobrame.core.reaction.TranscriptionReaction*

Parameters

- **id** (*str*) – Identifier of the transcribed gene. As a best practice, this ID should be prefixed with 'RNA + _'
- **RNA_type** (*str*) – Type of RNA encoded by gene sequence (mRNA, rRNA, tRNA, or ncRNA)
- **nucleotide_sequence** (*str*) – String of base pair abbreviations for nucleotides contained in the gene

left_pos
int – Left position of gene on the sequence of the (+) strain

right_pos
int – Right position of gene on the sequence of the (+) strain

strand
str –

- (+) if the RNA product is on the leading strand
- (-) if the RNA product is on the comple(mentary strand

nucleotide_count
Get number of each nucleotide monophosphate

Returns {nucleotide_monophosphate_id: count}

Return type *dict*

class cobrame.core.component.**TranslatedGene** (*id*)
Bases: *cobrame.core.component.MEComponent*

Metabolite class for protein created from *cobrame.core.reaction.TranslationReaction*

Parameters **id** (*str*) – Identifier of the translated protein product. Should be prefixed with "protein + _"

amino_acid_sequence
Get amino acid sequence of protein

Returns Amino acid sequence of protein

Return type str

complexes
Get the complexes that the protein forms

Returns List of *cobrame.core.component.Complex* s that the protein is a subunit of

Return type list

metabolic_reactions
Get the mtabolic reactions that the protein helps catalyze

Returns List of cobrame.core.reactions.MetabolicReaction s that the protein helps catalyze

Return type list

translation_data
Get translation data that defines protein.

Assumes that TranslatedGene is "protein + _ + <translation data id>"

Returns Translation data used to form translation reaction of protein

Return type *cobrame.core.processdata.TranslationData*

cobrame.core.component.**create_component**(*component_id*, *default_type=<class 'cobrame.core.component.MEComponent'>*, *rnap_set=set([])*)
creates a component and attempts to set the correct type

cobra.core.model module

class cobrame.core.model.**MEModel**(**args*)
Bases: cobra.core.Model.Model

add_biomass_constraints_to_model(*biomass_types*)

complex_data

compute_solution_error(*solution=None*)

construct_attribute_vector(*attr_name*, *growth_rate*)
build a vector of a reaction attribute at a specific growth rate

Mainly used for upper and lower bounds

construct_s_matrix(*growth_rate*)
build the stoichiometric matrix at a specific growth rate

gam

generic_data

get_metabolic_flux(*solution=None*)
extract the flux state for metabolic reactions

get_transcription_flux(*solution=None*)
extract the transcription flux state

get_translation_flux(*solution=None*)
extract the translation flux state

ngam

posttranslation_data

prune(*skip=None*)
remove all unused metabolites and reactions

This should be run after the model is fully built. It will be difficult to add new content to the model once this has been run.

skip: list List of complexes/proteins/mRNAs/TUs to remain unpruned from model.

remove_genes_from_model(*gene_list*)

set_sasa_keffs(*median_keff*)

stoichiometric_data

subreaction_data

tRNA_data

transcription_data

translation_data

translocation_data

unmodeled_protein

unmodeled_protein_biomass

unmodeled_protein_fraction

update()
updates all component reactions

Module contents

7.1.2 cobrame.util package

Submodules

cobrame.util.building module

cobrame.util.building.**add_complex_to_model**(*me_model*, *complex_id*, *complex_stoichiometry*, *complex_modifications=None*)
Adds ComplexData to the model for a given complex.

Parameters

- **me_model** (*cobrame.core.model.MEModel*) –
- **complex_id** (*str*) – ID of the complex and thus the model ComplexData
- **complex_stoichiometry** (*dict*) – {complex_id: {protein_<locus_tag>: stoichiometry}}
- **complex_modifications** (*dict*) – {subreaction_id: stoichiometry}

cobrame.util.building.**add_dummy_reactions**(*me_model*, *dna_seq*, *update=True*)
Add all reactions necessary to produce a dummy reaction catalyzed by "CPLX_dummy".

Parameters

- **me_model** (*cobrame.core.model.MEModel*) – The MEModel object to which the content will be added
- **dna_seq** (*str*) – DNA sequence of dummy gene. Should be representative of the average codon composition, amino acid composition, length of a gene in the organism being modeled
- **update** (*bool*) – If True, run update functions on all transcription, translation, complex formation, and metabolic reactions added when constructing dummy reactions.

cobrame.util.building.**add_m_model_content**(*me_model*, *m_model*, *complex_metabolite_ids=None*)
Add metabolite and reaction attributes to me_model from m_model. Also creates StoichiometricData objects for each reaction in m_model, and adds reactions directly to me_model if they are exchanges or demands.

Parameters

- **me_model** (*cobrame.core.model.MEModel*) – The MEModel object to which the content will be added
- **m_model** (cobra.core.model.Model) – The m_model which will act as the source of metabolic content for MEModel
- **complex_metabolite_ids** (*list*) – List of complexes which are 'metabolites' in the m-model reaction matrix, but should be treated as complexes

cobrame.util.building.**add_metabolic_reaction_to_model**(*me_model*, *stoichiometric_data_id*, *directionality*, *complex_id=None*, *spontaneous=False*, *update=False*, *keff=65*)

Creates and add a MetabolicReaction to a MEModel.

Parameters

- **me_model** (*cobrame.core.model.MEModel*) – MEModel that the MetabolicReaction will be added to
- **stoichiometric_data_id** (*str*) – ID of the StoichiometricData for the reaction being added
- **directionality** (*str*) –
  - Forward: Add reaction that occurs in the forward direction
  - Reverse: Add reaction that occurs in the reverse direction
- **complex_id** (*str or None*) – ID of the ComplexData for the enzyme that catalyze the reaction being added.
- **spontaneous** (*bool*) –
  - If True and complex_id=" add reaction as spontaneous reaction
  - If False and complex_id=" add reaction as orphan (CPLX_dummy catalyzed)

cobrame.util.building.**add_model_complexes**(*me_model*, *complex_stoichiometry_dict*, *complex_modification_dict*, *verbose=True*)

Construct ComplexData for complexes into MEModel from its subunit stoichiometry, and a dictionary of its modification metabolites.

It is assumed that each modification adds one equivalent of the modification metabolite. Multiple

Intended to be used as a function for large-scale complex addition.

For adding individual ComplexData objects, use add_complex_to_model

Parameters

- **me_model** (*cobrame.core.model.MEModel*) –
- **complex_stoichiometry_dict** (dict) – {unmodified_complex_id: {protein_<locus_tag>: stoichiometry}}
- **complex_modification_dict** (dict) –

  {modified_complex_id:{core_enzyme: unmodified_complex_id, 'modifications: {mod_metabolite: stoichiometry}}}

`cobrame.util.building.`**`add_reactions_from_stoichiometric_data`**(*me_model*, *rxn_to_cplx_dict*, *rxn_info_frame*, *update=False*, *keff=65*)

Creates and adds MetabolicReaction for all StoichiometricData in model.

Intended for use when adding all reactions from stoichiometric data for the first time.

For adding an individual reaction use add_metabolic_reaction_to_model()

Parameters

- **me_model** (*cobrame.core.model.MEModel*) – MEModel that the MetabolicReaction will be added to
- **rxn_to_cplx_dict** (*dict*) – {StoichiometricData.id: catalytic_enzyme_id}
- **rxn_info_frame** (pandas.Dataframe) – Contains the ids, names and reversibility for each reaction in the metabolic reaction matrix as well as whether the reaction is spontaneous

`cobrame.util.building.`**`add_subreaction_data`**(*me_model*, *modification_id*, *modification_stoichiometry*, *modification_enzyme=None*, *verbose=True*)

Creates a SubreactionData object for each modification defined by the function inputs.

It's assumed every complex modification occurs spontaneously, unless a modification_enzyme argument is passed.

If a modification uses an enzyme this can be updated after the SubreactionData object is already created

Parameters **me_model** (*cobrame.core.model.MEModel*) –

`cobrame.util.building.`**`add_transcription_reaction`**(*me_model*, *tu_name*, *locus_ids*, *sequence*, *update=True*)

Create TranscriptionReaction object and add it to ME-Model. This includes the necessary transcription data.

Parameters

- **me_model** (*cobrame.core.model.MEModel*) – The MEModel object to which the reaction will be added
- **tu_name** (*str*) – ID of TU being transcribed. The TranscriptionReaction will be added as "transcription_+TU_name" The TranscriptionData will be added as just "TU_name"
- **locus_ids** (*set*) – Set of locus IDs that the TU transcribes
- **sequence** (*str*) – Nucleotide sequence of the TU.
- **update** (*bool*) – If True, use TranscriptionReaction's update function to update and add reaction stoichiometry

Returns TranscriptionReaction for the TU

Return type *cobrame.core.reaction.TranscriptionReaction*

`cobrame.util.building.`**`add_translation_reaction`**(*me_model*, *locus_id*, *dna_sequence*, *update=False*)

Creates and adds a TranslationReaction to the ME-model as well as the associated TranslationData

A dna_sequence is required in order to add a TranslationReaction to the ME-model

Parameters

- **me_model** (cobra.core.model.MEModel) – The MEModel object to which the reaction will be added
- **locus_id** (*str*) – Locus ID of RNA product. The TranslationReaction will be added as "translation + _ + locus_id" The TranslationData will be added as "locus_id"
- **dna_sequence** (*str*) – DNA sequence of the RNA product. This string should be reverse transcribed if it originates on the complement strand.
- **update** (*bool*) – If True, use TranslationReaction's update function to update and add reaction stoichiometry

cobrame.util.building.**build_reactions_from_genbank**(*me_model, gb_filename, tu_frame=None, element_types=set(['tRNA', 'ncRNA', 'rRNA', 'CDS']), verbose=True, frameshift_dict=None, trna_to_codon=None, update=True*)

Creates and adds transcription and translation reactions using genomic information from the organism's genbank file. Adds in the basic requirements for these reactions. Organism specific components are added ...

Parameters

- **me_model** (*cobrame.core.model.MEModel*) – The MEModel object to which the reaction will be added
- **gb_filename** (*str*) – Local name of the genbank file that will be used for ME-model construction
- **tu_frame** (pandas.DataFrame) – DataFrame with indexes of the transcription unit name and columns containing the transcription unit starting and stopping location on the genome and whether the transcription unit is found on the main (+) strand or complementary (-) strand.

  If no transcription unit DataFrame is passed into the function, transcription units are added corresponding to each transcribed gene in the genbank file.
- **element_types** (*set*) – Transcription reactions will be added to the ME-model for all RNA feature.types in this set. This uses the nomenclature of the genbank file (gb_filename)
- **verbose** (*bool*) – If True, display metabolites that were not previously added to the model and were thus added when creating charging reactions
- **frameshift_dict** (dict) – {locus_id: genome_position_of_TU}

  If a locus_id is in the frameshift_dict, update it's nucleotide sequence to account of the frameshift

cobrame.util.building.**convert_aa_codes_and_add_charging**(*me_model, trna_aa, trna_to_codon, verbose=True*)

Adds tRNA charging reactions for all tRNAs in ME-model

Parameters

- **me_model** (cobra.core.model.MEModel) – The MEModel object to which the reaction will be added

- **trna_aa** (dict) – Dictionary of tRNA locus ID to 3 letter codes of the amino acid that the tRNA contributes

  {tRNA identifier (locus_id): amino_acid_3_letter_code}

- **trna_to_codon** (dict) – Dictionary of tRNA identifier to the codon which it associates

  {tRNA identifier (locus_id): codon_sequence}

- **verbose** (*bool*) – If True, display metabolites that were not previously added to the model and were thus added when creating charging reactions

cobrame.util.building.**create_transcribed_gene**(*me_model*, *locus_id*, *rna_type*, *seq*, *left_pos=None*, *right_pos=None*, *strand=None*)

Creates a *TranscribedGene* metabolite object and adds it to the ME-model

Parameters

- **me_model** (*cobrame.core.model.MEModel*) – The MEModel object to which the reaction will be added
- **locus_id** (*str*) – Locus ID of RNA product. The TranscribedGene will be added as "RNA + _ + locus_id"
- **left_pos** (*int or None*) – Left position of gene on the sequence of the (+) strain
- **right_pos** (*int or None*) – Right position of gene on the sequence of the (+) strain
- **seq** (*str*) – Nucleotide sequence of RNA product. Amino acid sequence, codon counts, etc. will be calculated based on this string.
- **strand** (*str or None*) –
  - (+) if the RNA product is on the leading strand
  - (-) if the RNA product is on the complementary strand
- **rna_type** (*str*) – Type of RNA of the product. tRNA, rRNA, or mRNA Used for determining how RNA product will be processed.

Returns Metabolite object for the RNA product

Return type *cobrame.core.component.TranscribedGene*

cobrame.util.dogma module

cobrame.util.dogma.**extract_sequence**(*full_seq*, *left_pos*, *right_pos*, *strand*)

cobrame.util.dogma.**get_amino_acid_sequence_from_dna**(*dna_seq*)

cobrame.util.dogma.**return_frameshift_sequence**(*full_seq*, *frameshift_string*)

cobrame.util.dogma.**reverse_transcribe**(*seq*)

cobrame.util.mass module

cobra.core.massbalance module

cobrame.util.massbalance.**check_me_model_mass_balance**(*model0*)

cobrame.util.massbalance.**check_transcription_mass_balance**(*reaction*)

cobrame.util.massbalance.**elements_to_formula**(*obj*, *elements*)

cobrame.util.massbalance.**eval_reaction_at_growth_rate**(*reaction*, *growth_rate*)

cobrame.util.massbalance.**get_elements_from_process_data**(*reaction*, *process_data*, *elements*)

If a modification is required to form a functioning macromolecule, update the element dictionary accordingly.

cobrame.util.massbalance.**stringify**(*element*, *number*)

Module contents

7.1.3 cobrame.io package

Submodules

cobrame.io.dict module

cobrame.io.dict.**get_numeric_from_string**(*string*)

Parameters **string** (*str*) – String representation of numeric expression

Returns Numeric representation of string

Return type float or sympy expression

cobrame.io.dict.**get_sympy_expression**(*value*)

Return sympy expression from json string using sympify

mu is assumed to be positive but using sympify does not apply this assumption. The mu symbol produced from sympify is replaced with cobrame's mu value to ensure the expression can be used in the model.

Parameters **value** (*str*) – String representation of mu containing expression

Returns Numeric representation of string with cobrame's mu symbol substituted

Return type sympy expression

cobrame.io.dict.**me_model_from_dict**(*obj*)

Load ME-model from its dictionary representation. This will return a full *MEModel* object identical to the one saved.

Parameters **obj** (*dict*) – Dictionary representation of ME-model

Returns Full COBRAme ME-model

Return type *MEModel*

cobrame.io.dict.**me_model_to_dict**(*model*)

Create dictionary representation of full ME-model

Parameters **model** (*MEModel*) –

Returns Dictionary representation of ME-model

Return type *dict*

cobrame.io.json module

cobrame.io.json.**get_schema**()

Load JSON schema for ME-model JSON saving/loading

Returns JSONSCHEMA

Return type *dict*

`cobrame.io.json.`**`load_json_me_model`**(*file_name*)

Load a full JSON version of the ME-model. Loading a model in this format will return a ME-model identical to the one saved, which retains all ME-model functionality.

Parameters **`file_name`** (*`str or file-like object`*) – Filename of the JSON output or an open json file

Returns A full ME-model

Return type *`cobrame.core.model.MEModel`*

`cobrame.io.json.`**`load_reduced_json_me_model`**(*file_name*)

Load a stripped-down JSON version of the ME-model. This will exclude all of ME-Model information except the reaction stoichiometry information and the reaction bounds. Saving/loading a model in this format will thus occur much quicker, but limit the ability to edit the model and use most of its features.

Parameters **`file_name`** (*`str or file-like object`*) – Filename of the JSON ME-model

Returns COBRA Model representation of the ME-model. This will not include all of the functionality of a *`MEModel`* but will solve identically compared to the full model.

Return type `cobra.core.model.Model`

`cobrame.io.json.`**`save_json_me_model`**(*model, file_name*)

Save a full JSON version of the ME-model. Saving/loading a model in this format can then be loaded to return a ME-model identical to the one saved, which retains all ME-model functionality.

Parameters

- **`model`** (*`cobrame.core.model.MEModel`*) – A full ME-model
- **`file_name`** (*`str or file-like object`*) – Filename of the JSON output or an open json file

`cobrame.io.json.`**`save_reduced_json_me_model`**(*me0, file_name*)

Save a stripped-down JSON version of the ME-model. This will exclude all of ME-Model information except the reaction stoichiometry information and the reaction bounds. Saving/loading a model in this format will thus occur much quicker, but limit the ability to edit the model and use most of its features.

Parameters

- **`me0`** (*`cobrame.core.model.MEModel`*) – A full ME-model
- **`file_name`** (*`str or file-like object`*) – Filename of the JSON output

Module contents

7.2 Module contents

CHAPTER 8

Indices and tables

- genindex
- modindex
- search

Python Module Index

c

cobrame, 69
cobrame.core, 63
cobrame.core.component, 59
cobrame.core.model, 62
cobrame.core.processdata, 51
cobrame.core.reaction, 45
cobrame.io, 69
cobrame.io.dict, 68
cobrame.io.json, 68
cobrame.util, 68
cobrame.util.building, 63
cobrame.util.dogma, 67
cobrame.util.massbalance, 67

Index

Symbols

_complex_id (cobrame.core.reaction.ComplexFormation attribute), 45
_element_contribution (SubreactionData attribute), 19
_element_contribution (cobrame.core.processdata.SubreactionData attribute), 55
_stoichiometry (StoichiometricData attribute), 22
_stoichiometry (cobrame.core.processdata.StoichiometricData attribute), 54

A

add_biomass_constraints_to_model() (cobrame.core.model.MEModel method), 62
add_biomass_from_subreactions() (cobrame.core.reaction.MEReaction method), 46
add_complex_to_model() (in module cobrame.util.building), 63
add_dummy_reactions() (in module cobrame.util.building), 63
add_elongation_subreactions() (cobrame.core.processdata.TranslationData method), 57
add_initiation_subreactions() (cobrame.core.processdata.TranslationData method), 57
add_m_model_content() (in module cobrame.util.building), 63
add_metabolic_reaction_to_model() (in module cobrame.util.building), 64
add_model_complexes() (in module cobrame.util.building), 64
add_reactions_from_stoichiometric_data() (in module cobrame.util.building), 64
add_subreaction_data() (in module cobrame.util.building), 65
add_subreactions() (cobrame.core.reaction.MEReaction method), 46
add_termination_subreactions() (cobrame.core.processdata.TranslationData method), 57
add_transcription_reaction() (in module cobrame.util.building), 65
add_translation_reaction() (in module cobrame.util.building), 65
add_translocation_pathways() (cobrame.core.reaction.PostTranslationReaction method), 48
aggregation_propensity (cobrame.core.processdata.PostTranslationData attribute), 53
amino_acid_count (cobrame.core.processdata.TranslationData attribute), 57
amino_acid_sequence (cobrame.core.component.TranslatedGene attribute), 61
amino_acid_sequence (cobrame.core.processdata.TranslationData attribute), 58

B

biomass_type (cobrame.core.processdata.PostTranslationData attribute), 53
build_reactions_from_genbank() (in module cobrame.util.building), 66

C

calculate_biomass_contribution() (cobrame.core.processdata.SubreactionData method), 55
calculate_element_contribution() (cobrame.core.processdata.SubreactionData method), 55
check_me_mass_balance() (cobrame.core.reaction.MEReaction method), 47
check_me_model_mass_balance() (in module cobrame.util.massbalance), 67

check_transcription_mass_balance() (in module cobrame.util.massbalance), 67
clear_metabolites() (cobrame.core.reaction.MEReaction method), 47
cobrame (module), 69
cobrame.core (module), 63
cobrame.core.component (module), 59
cobrame.core.model (module), 62
cobrame.core.processdata (module), 51
cobrame.core.reaction (module), 45
cobrame.io (module), 69
cobrame.io.dict (module), 68
cobrame.io.json (module), 68
cobrame.util (module), 68
cobrame.util.building (module), 63
cobrame.util.dogma (module), 67
cobrame.util.massbalance (module), 67
codes_stable_rna (cobrame.core.processdata.TranscriptionData attribute), 56
codon_count (cobrame.core.processdata.TranslationData attribute), 58
Complex (class in cobrame.core.component), 59
complex (cobrame.core.processdata.ComplexData attribute), 52
complex (cobrame.core.reaction.ComplexFormation attribute), 45
complex_data (cobrame.core.model.MEModel attribute), 62
complex_data (cobrame.core.reaction.MetabolicReaction attribute), 48
complex_data_id (cobrame.core.reaction.ComplexFormation attribute), 45
complex_id (cobrame.core.processdata.ComplexData attribute), 52
ComplexData (class in cobrame.core.processdata), 51
complexes (cobrame.core.component.TranslatedGene attribute), 61
ComplexFormation (class in cobrame.core.reaction), 45
compute_solution_error() (cobrame.core.model.MEModel method), 62
Constraint (class in cobrame.core.component), 59
construct_attribute_vector() (cobrame.core.model.MEModel method), 62
construct_s_matrix() (cobrame.core.model.MEModel method), 62
convert_aa_codes_and_add_charging() (in module cobrame.util.building), 66
create_complex_formation() (cobrame.core.processdata.ComplexData method), 52
create_component() (in module cobrame.core.component), 62
create_reactions() (cobrame.core.processdata.GenericData method), 52
create_transcribed_gene() (in module cobrame.util.building), 67

E

element_contribution (cobrame.core.processdata.SubreactionData attribute), 55
elements_to_formula() (in module cobrame.util.massbalance), 67
enzyme (cobrame.core.processdata.SubreactionData attribute), 55
enzyme (SubreactionData attribute), 19
enzyme_dict (cobrame.core.processdata.TranslocationData attribute), 58
eval_reaction_at_growth_rate() (in module cobrame.util.massbalance), 68
excised_bases (cobrame.core.processdata.TranscriptionData attribute), 56
extract_sequence() (in module cobrame.util.dogma), 67

F

first_codon (cobrame.core.processdata.TranslationData attribute), 58
folding_mechanism (cobrame.core.processdata.PostTranslationData attribute), 53
formation (cobrame.core.processdata.ComplexData attribute), 52

G

gam (cobrame.core.model.MEModel attribute), 62
generic_data (cobrame.core.model.MEModel attribute), 62
GenericComponent (class in cobrame.core.component), 59
GenericData (class in cobrame.core.processdata), 52
GenericFormationReaction (class in cobrame.core.reaction), 46
GenerictRNA (class in cobrame.core.component), 60
get_all_usages() (cobrame.core.processdata.SubreactionData method), 55
get_amino_acid_sequence_from_dna() (in module cobrame.util.dogma), 67
get_complex_data() (cobrame.core.processdata.SubreactionData method), 55
get_components_from_ids() (cobrame.core.reaction.MEReaction method), 47
get_elements_from_process_data() (in module cobrame.util.massbalance), 68
get_metabolic_flux() (cobrame.core.model.MEModel method), 62

get_numeric_from_string() (in module cobrame.io.dict), 68
get_schema() (in module cobrame.io.json), 68
get_sympy_expression() (in module cobrame.io.dict), 68
get_transcription_flux() (cobrame.core.model.MEModel method), 62
get_translation_flux() (cobrame.core.model.MEModel method), 62

K

k_folding (cobrame.core.processdata.PostTranslationData attribute), 53
keff (cobrame.core.processdata.SubreactionData attribute), 55
keff (cobrame.core.processdata.TranslocationData attribute), 58
keff (cobrame.core.reaction.MetabolicReaction attribute), 47
keff (MetabolicReaction attribute), 23
keff (SubreactionData attribute), 19
keq_folding (cobrame.core.processdata.PostTranslationData attribute), 53

L

last_codon (cobrame.core.processdata.TranslationData attribute), 58
left_pos (cobrame.core.component.TranscribedGene attribute), 61
left_pos (TranscribedGene attribute), 11
length_dependent_energy (cobrame.core.processdata.TranslocationData attribute), 58
load_json_me_model() (in module cobrame.io.json), 69
load_reduced_json_me_model() (in module cobrame.io.json), 69
lower_bound (cobrame.core.processdata.StoichiometricData attribute), 54
lower_bound (StoichiometricData attribute), 23

M

me_model_from_dict() (in module cobrame.io.dict), 68
me_model_to_dict() (in module cobrame.io.dict), 68
MEComponent (class in cobrame.core.component), 60
MEModel (class in cobrame.core.model), 62
MEReaction (class in cobrame.core.reaction), 46
metabolic_reactions (cobrame.core.component.Complex attribute), 59
metabolic_reactions (cobrame.core.component.TranslatedGene attribute), 61
MetabolicReaction (class in cobrame.core.reaction), 47
Metabolite (class in cobrame.core.component), 60
model (cobrame.core.processdata.ProcessData attribute), 54
mRNA (cobrame.core.processdata.TranslationData attribute), 57
mRNA (TranslationData attribute), 14

N

ngam (cobrame.core.model.MEModel attribute), 62
nucleotide_count (cobrame.core.component.TranscribedGene attribute), 61
nucleotide_count (cobrame.core.processdata.TranscriptionData attribute), 56
nucleotide_sequence (cobrame.core.processdata.TranscriptionData attribute), 56
nucleotide_sequence (cobrame.core.processdata.TranslationData attribute), 57
nucleotide_sequence (TranscriptionData attribute), 12
nucleotide_sequence (TranslationData attribute), 15

O

objective_coefficient (cobrame.core.reaction.MEReaction attribute), 47

P

parent_reactions (cobrame.core.processdata.ProcessData attribute), 54
posttranslation_data (cobrame.core.model.MEModel attribute), 62
posttranslation_data (cobrame.core.reaction.PostTranslationReaction attribute), 48
PostTranslationData (class in cobrame.core.processdata), 52
PostTranslationReaction (class in cobrame.core.reaction), 48
ProcessData (class in cobrame.core.processdata), 53
ProcessedProtein (class in cobrame.core.component), 60
propensity_scaling (cobrame.core.processdata.PostTranslationData attribute), 53
protein (cobrame.core.processdata.TranslationData attribute), 57
protein (TranslationData attribute), 14
prune() (cobrame.core.model.MEModel method), 62

R

remove_from_me_model() (cobrame.core.component.MEComponent method), 60
remove_genes_from_model() (cobrame.core.model.MEModel method), 62
return_frameshift_sequence() (in module cobrame.util.dogma), 67

reverse (cobrame.core.reaction.MetabolicReaction attribute), 47
reverse (MetabolicReaction attribute), 23
reverse_transcribe() (in module cobrame.util.dogma), 67
Ribosome (class in cobrame.core.component), 60
right_pos (cobrame.core.component.TranscribedGene attribute), 61
right_pos (TranscribedGene attribute), 11
RNA_polymerase (cobrame.core.processdata.TranscriptionData attribute), 56
RNA_polymerase (TranscriptionData attribute), 12
RNA_products (cobrame.core.processdata.TranscriptionData attribute), 56
RNA_products (TranscriptionData attribute), 12
RNA_types (cobrame.core.processdata.TranscriptionData attribute), 56
RNAP (class in cobrame.core.component), 60

S

save_json_me_model() (in module cobrame.io.json), 69
save_reduced_json_me_model() (in module cobrame.io.json), 69
set_sasa_keffs() (cobrame.core.model.MEModel method), 62
stoichiometric_data (cobrame.core.model.MEModel attribute), 62
stoichiometric_data (cobrame.core.reaction.MetabolicReaction attribute), 48
StoichiometricData (class in cobrame.core.processdata), 54
stoichiometry (cobrame.core.processdata.ComplexData attribute), 51
stoichiometry (cobrame.core.processdata.StoichiometricData attribute), 54
stoichiometry (cobrame.core.processdata.TranslocationData attribute), 59
stoichiometry (ComplexData attribute), 20
strand (cobrame.core.component.TranscribedGene attribute), 61
strand (TranscribedGene attribute), 12
stringify() (in module cobrame.util.massbalance), 68
subreaction_data (cobrame.core.model.MEModel attribute), 62
SubreactionData (class in cobrame.core.processdata), 54
subreactions (cobrame.core.processdata.ComplexData attribute), 52
subreactions (cobrame.core.processdata.PostTranslationData attribute), 53
subreactions (cobrame.core.processdata.StoichiometricData attribute), 54
subreactions (cobrame.core.processdata.TranscriptionData attribute), 56
subreactions (cobrame.core.processdata.TranslationData attribute), 57
subreactions (cobrame.core.processdata.tRNAData attribute), 59
subreactions (ComplexData attribute), 21
subreactions (StoichiometricData attribute), 22
subreactions (TranscriptionData attribute), 12
subreactions (TranslationData attribute), 15
subreactions (tRNAData attribute), 17
subreactions_from_sequence (cobrame.core.processdata.TranslationData attribute), 58
SummaryVariable (class in cobrame.core.reaction), 49
surface_area (cobrame.core.processdata.PostTranslationData attribute), 53
synthetase (cobrame.core.processdata.tRNAData attribute), 59
synthetase (tRNAData attribute), 17
synthetase_keff (cobrame.core.processdata.tRNAData attribute), 59
synthetase_keff (tRNAData attribute), 17

T

TranscribedGene (class in cobrame.core.component), 61
transcription_data (cobrame.core.model.MEModel attribute), 63
transcription_data (cobrame.core.reaction.TranscriptionReaction attribute), 49
TranscriptionData (class in cobrame.core.processdata), 56
TranscriptionReaction (class in cobrame.core.reaction), 49
TranslatedGene (class in cobrame.core.component), 61
translation_data (cobrame.core.component.TranslatedGene attribute), 61
translation_data (cobrame.core.model.MEModel attribute), 63
translation_data (cobrame.core.reaction.TranslationReaction attribute), 50
TranslationData (class in cobrame.core.processdata), 57
TranslationReaction (class in cobrame.core.reaction), 50
translocation (cobrame.core.processdata.PostTranslationData attribute), 53
translocation_data (cobrame.core.model.MEModel attribute), 63
translocation_multipliers (cobrame.core.processdata.PostTranslationData attribute), 53
TranslocationData (class in cobrame.core.processdata), 58
tRNA_data (cobrame.core.model.MEModel attribute), 62
tRNA_data (cobrame.core.reaction.tRNAChargingReaction attribute), 51
tRNAChargingReaction (class in cobrame.core.reaction), 51

tRNAData (class in cobrame.core.processdata), 59

U

unmodeled_protein (cobrame.core.model.MEModel attribute), 63
unmodeled_protein_biomass (cobrame.core.model.MEModel attribute), 63
unmodeled_protein_fraction (cobrame.core.model.MEModel attribute), 63
unprocessed_protein (cobrame.core.component.ProcessedProtein attribute), 60
update() (cobrame.core.model.MEModel method), 63
update() (cobrame.core.reaction.ComplexFormation method), 45
update() (cobrame.core.reaction.MetabolicReaction method), 48
update() (cobrame.core.reaction.PostTranslationReaction method), 49
update() (cobrame.core.reaction.TranscriptionReaction method), 49
update() (cobrame.core.reaction.TranslationReaction method), 50
update() (cobrame.core.reaction.tRNAChargingReaction method), 51
update_parent_reactions() (cobrame.core.processdata.ProcessData method), 54
upper_bound (cobrame.core.processdata.StoichiometricData attribute), 54
upper_bound (StoichiometricData attribute), 22
