# Supplementary material for: COBRAme: A computational framework for genome-scale models of metabolism and gene expression
Source: PLoS Comput Biol. 2018 Jul 5;14(7):e1006302. doi: 10.1371/journal.pcbi.1006302 (PMC6049947; doi:10.1371/journal.pcbi.1006302)
Supplement: S8 File — Includes additional information regarding ME-model updates and reformulations along with some additional analysis. (PDF) [file pcbi.1006302.s008.pdf]

COBRAme: A Computational Framework for Genome-Scale Models of Metabolism and Gene Expression

Colton J. Lloyd*, Ali Ebrahim*, Laurence Yang, Zachary A. King, Edward Catoiu, Edward J. O'Brien, Joanne K. Liu, Bernhard O. Palsson

Supplementary Text

ME-model Reformulation 1
Formation of tRNA Equivalents 2
Stable RNA Splicing 4
Coupling Constraint Implementation 5
Unmodeled Protein Fraction 7
Braun's Lipoprotein Demand 7
Lipoprotein Reactions 8

Model Improvement and Additions 8
Dummy Complex / Orphan Reactions 8
Modeling of Protein Carriers 9
Solve Procedure Simplification 9
Model Updates and Corrections 10
Parameter Updates and Corrections 13

ME-model Characteristics 13

Supplementary Figures 15

ME-model Reformulation

The ME-model coupling constraint formulation and implementation was altered from O'Brien et al [1]. These changes allowed a variety of improvements in ME-model size, solve time, and user interpretation. The major changes made to tRNA charging reactions, coupling constraint implementation, and the application of the biomass constraint are summarized in the

following sections.

Formation of tRNA Equivalents

A major goal of this work was to facilitate modular construction of ME-models (i.e. with the ability to produce models with various macromolecules or expression processes removed). To this end, the tRNA coupling to translation was reformulated. This was done in order to account for the dilution coupling of both the tRNA synthetase and tRNAs themselves during tRNA charging reactions. In doing so, the tRNA charging reactions now produce a charged tRNA equivalent. These tRNA equivalents can be included in the translation reaction directly without a coupling coefficient since dilution coupling has already been accounted for in the charging reaction. This allows tRNAs to be cleanly removed without breaking the model by simply deleting the SubreactionData instances that apply the charged tRNA equivalents.

To do this, the amino acid metabolite was uncoupled from its charged tRNA using the following derivation.

Ignoring energy requirements, the overall tRNA charging reaction can be written as followed:

$$\mathrm{AA} + \mathrm{tRNA} + \frac{\mu}{\mathrm{k_{eff,charging}}}\mathrm{synthetase} \rightarrow \mathrm{tRNA}^* \tag{1}$$

where tRNA^* corresponds to a charged tRNA.

During translation, a tRNA is used to make the protein. However, because the tRNA is being used as a catalyst, its synthesis must be coupled to its use during translation with a coupling constraint. Therefore when the tRNA is used during translation, it is applied as follows:

$$\left(1 + \frac{\mu}{\mathrm{k_{eff,tRNA}}}\right)\mathrm{tRNA}^* \rightarrow \mathrm{tRNA} + \mathrm{protein_{i+1}} \tag{2}$$

Where i is the length of the protein before the charged tRNA is applied and $\frac{\mu}{\mathrm{k_{eff,tRNA}}}$ represents the coupling of charged tRNA dilution to charged tRNA mediated amino acid addition (See **Coupling Constraint Implementation** below).

Plugging (1) into (2) gives (3):

$$\left(1+\frac{\mu}{\mathrm{k_{eff,tRNA}}}\right)\left(\mathrm{AA}+\mathrm{tRNA}+\frac{\mu}{\mathrm{k_{eff,charging}}}\mathrm{synthetase}\right)\rightarrow \\ \mathrm{tRNA}+\mathrm{protein_{i+1}} \tag{3}$$

Multiplying out (3) gives (4):

$$\mathrm{AA}+\mathrm{tRNA}+\left(1+\frac{\mu}{\mathrm{k_{eff,tRNA}}}\right)\frac{\mu}{\mathrm{k_{eff,charging}}}\mathrm{synthetase}+ \\ \frac{\mu}{\mathrm{k_{eff,tRNA}}}(\mathrm{tRNA}+\mathrm{AA})\rightarrow \mathrm{tRNA}+\mathrm{protein_{i+1}} \tag{4}$$

The tRNA metabolite shows up on both sides of the equation and can be dropped giving (5):

$$\mathrm{AA}+\left(1+\frac{\mu}{\mathrm{k_{eff,tRNA}}}\right)\frac{\mu}{\mathrm{k_{eff,charging}}}\mathrm{synthetase}+ \\ \frac{\mu}{\mathrm{k_{eff,tRNA}}}(\mathrm{tRNA}+\mathrm{AA})\rightarrow \mathrm{protein_{i+1}} \tag{5}$$

Equation (5) can be split into both the **charging reaction** (6) and **translation reaction** (7):

$$\left(1+\frac{\mu}{\mathrm{k_{eff,tRNA}}}\right)\frac{\mu}{\mathrm{k_{eff,charging}}}\mathrm{synthetase}+ \\ \frac{\mu}{\mathrm{k_{eff,tRNA}}}(\mathrm{tRNA}+\mathrm{AA})\rightarrow \mathrm{generic\_tRNA_{AA}} \tag{6}$$

$$\mathrm{AA}+\mathrm{generic\_tRNA_{AA}}\rightarrow \mathrm{protein_{i+1}} \tag{7}$$

where generic_tRNA$_{AA}$ is a pseudo-metabolite that represents a charged tRNA equivalent with tRNA coupling already applied. Using the formulation of tRNA charging and translation in (6) and (7), respectively, the user is able to cleanly remove the tRNA process from the model by simply removing generic_tRNA$_{AA}$ from the model or translation reaction. This is useful for constructing ME-models for organisms in which tRNA charging is not well reconstructed, for reducing the complexity of the model (and thus solve time), and for investigating the role of tRNA charging at a systems level.

Stable RNA Splicing

When transcription units containing stable RNAs (tRNA, rRNA, or ncRNA) are transcribed, the individual RNAs must be excised from the transcript using various endonucleases. Previous *E. coli* ME-models included individual reactions for all the different possible combinations which a transcription unit (TU) can be excised of rRNA, tRNA, or ncRNA. Only 82 transcription reactions (representing the 82 modeled transcription units used to express each of the stable RNA molecules) are present in *i*JL1678b-ME. Accounting for all excision possibilities and the subsequent degradation of the excised noncoding TU portions, however, adds thousands of reactions to the model. This has a sizable effect on the solve time of the ME-model with limited improvement in the predictive capability of the model, therefore the handling of this was removed from *i*JL1678b-ME.

In *i*OL1650-ME, this was modeled by accounting for all possible combinations of RNA excision as separate individual reactions. For instance, if a TU_1 contains rRNA_1 and rRNA_2 which code at the first and last position on TU_1, respectively, it would require three separate reactions:

$$\begin{aligned} \text{TU\_1} &\rightarrow \text{excised\_portion\_1} + \text{rRNA\_1} \\ \text{TU\_1} &\rightarrow \text{rRNA\_2} + \text{excised\_portion\_2} \\ \text{TU\_1} &\rightarrow \text{rRNA\_1} + \text{excised\_portion\_3} + \text{rRNA\_2} \end{aligned} \quad (8)$$

Each of the excised portion metabolites shown in (8) would then require a separate reaction to degrade the excised portions back into nucleotide building blocks. While this is a rigorous way of modeling this phenomenon, it can result in a single TU requiring up to 127 reactions in order to be transcribed. To bypass this combinatorial problem, each TU in the new ME-model is transcribed in one single reaction that also degrades all of the excised portions giving the overall reaction. It is shown below ignoring excision and degradation enzymes:

$$\text{TU\_1} \rightarrow \text{rRNA\_1} + \text{rRNA\_2} + \text{x} \cdot \text{nucleotides} \quad (9)$$

Coupling Constraint Implementation

The previous iterations of ME-models applied coupling constraints in the following way:

$$\begin{aligned}\textbf{Enzyme Priming: } & \text{enzyme} \xrightarrow{v0} \text{enzyme\_primed} + \alpha \cdot \text{coupling} \\ \textbf{Enzymatic Reaction: } & \text{a} + \text{enzyme\_primed} \xrightarrow{v1} \text{enzyme} + \text{b} \\ \textbf{Dilution Coupling Reaction: } & \text{enzyme} + \text{coupling} \xrightarrow{v2} \emptyset\end{aligned} \tag{10}$$

Where α is the coupling coefficient applied to "coupling", a pseudo metabolite (constraint) which effectively determines the minimal rate that the third dilution coupling reaction ($v2$) must proceed. For previous ME-model implementations, this coupling constraint was given a "_constraint_sense" in COBRApy of 'L' (less than or equal to) meaning that for this toy example $v0 = v1$ and $v2 \geq \alpha \cdot v1$.

With COBRAme it is assumed that the optimal ME solution will not dilute more enzyme than is required by the coupling constraint, thus constraining $v2 = \alpha \cdot v1$. Further, the coupling of the metabolite (constraint) was imposed directly in the reaction that uses the enzyme to give.

$$\text{a} + \alpha \cdot \text{enzyme} \xrightarrow{v1} \text{b} \tag{11}$$

Reformulation the model to impose the coupling coefficients directly into the reaction in which they participate (equation (11), **Figure 3**) resulted in a reduced ME-matrix. This reformulation allowed the removal of coupling constraints (i.e. the "coupling" and "enzyme_primed" pseudo metabolites shown above in (10)) along with the associated variables (i.e. reactions v_0 and v_2 in (10)) used in previous ME-models to apply the macromolecular coupling. Removing these pseudo metabolites and reactions makes the ME-matrix notably smaller and decreases the solve time.

The above reformulation also reduces the space of feasible fluxes at suboptimal growth rates by eliminating inequality constraints. At an optimum, a ME-model will not waste resources, and, as a result, all the computed values are pushed up against their inequality constraints, effectively rendering them as equalities. Therefore, reformulating the model with equalities alone will compute the same optimal flux state but results in a much simpler numerical problem when applying the binary search or bisection solving algorithm (see **Optimization Procedure**).

As mentioned above, previous ME-model formulations have applied the coupling constraints as inequalities, thus allowing the simulation to synthe-

size macromolecule components above the value dictated by the coupling coefficient. Imposing the constraints as equalities, however, means that each COBRAme ME-model solution will synthesize the exact amount of each macromolecule as dictated by the coupling coefficient, thus giving the computed optimal macromolecule synthesis fluxes for the *in silico* conditions. While all enzymes are not fully saturated in *E. coli in vivo*, this phenomenon would not be selected as an optimal ME-model solution.

The COBRAme ME-model optimization problem is shown below using variable names and nomenclature from [1].

$$
\begin{aligned}
\max_{v,\mu}\ & \mu \\
\text{s.t. } & Sv = 0 \\
& v_{\text{formation,Ribosome}} - \sum_{i \in Peptide} \left(\frac{l_{p,i}}{c_{\text{ribo}} \kappa_\tau} (\mu + r_0 \kappa_\tau) \cdot v_{\text{translation},i} \right) = 0 \\
& v_{\text{formation,RNAP}} - \sum_{i \in TU} \left(\frac{l_{\text{TU},i}}{3 c_{\text{ribo}} \kappa_\tau} (\mu + r_0 \kappa_\tau) \cdot v_{\text{transcription},i} \right) = 0 \\
& v_{\text{formation},j} \\
& \quad - \sum_{i \in \text{generic\_tRNA}_{AA}} \left(\left(1 + \frac{\mu}{k_{eff,tRNA}} \right) \frac{\mu}{k_{eff,charging}} v_{\text{charging},i} \right) = 0, \\
& \quad \forall j \in Synthetase \\
& v_{\text{formation},j} - \sum_{i \in \text{enzymatic reaction}} \left(\frac{\mu}{k_{ij}^{\text{eff}}} v_{\text{usage},i} \right) = 0, \quad \forall j \in Enzyme \\
& v_{\text{formation},j} - \sum_{i \in \text{tRNA anticodons}} \frac{(\mu + \kappa_\tau r_0)}{\kappa_\tau c_{tRNA,j}} v_{charging,i} = 0, \quad \forall j \in tRNA \\
& v_{\text{degredation},j} - \frac{k_{deg,j}}{3 \kappa_\tau c_{mRNA}} \cdot \frac{\mu + \kappa_\tau r_0}{\mu} v_{translation,j} = 0, \quad \forall j \in mRNA \\
& v_{\text{formation},j} - \frac{(\mu + \kappa_\tau r_0)}{3 \kappa_\tau c_{mRNA}} v_{translation,j} = 0, \quad \forall j \in mRNA \\
& v^L \le v \le v^U \\
& \mu \le v_{biomass\_dilution} \le \mu
\end{aligned}
$$

Unmodeled Protein Fraction

Not all of the protein expressed in *E. coli* K-12 MG1655 is represented or active in a ME-model. To account for this discrepancy, the unmodeled protein fraction parameter is used. This parameter ensures that, in addition to the total protein biomass synthesized by the model to carry out enzymatic functions, an amount of wasted "unmodeled protein biomass" (dictated by the unmodeled protein fraction, Q) must be synthesized. This "unmodeled protein biomass" represents the unmodeled or underused protein in the ME-model.

The unmodeled protein fraction parameter was applied in the "protein_biomass_to_biomass" reaction. This reaction converts the protein_biomass and unmodeled_protein_biomass constraints to the overall biomass constraint and imposes the unmodeled protein fraction parameter. It has the form of (12)

$$\text{protein\_biomass} + \text{n} \cdot \text{unmodeled\_protein\_biomass} \rightarrow (1 + \text{n}) \cdot \text{biomass} \tag{12}$$

where:

$$Q = \frac{n}{1 + n} \tag{13}$$

Since the unmodeled protein fraction is intended to constrain the fraction of unmodeled_protein_biomass (n) relative to the total protein contribution to biomass ($1 + n$) to be equal to Q. Solving for Q gives (14).

$$n = \frac{Q}{1 - Q} \tag{14}$$

The expression for n as a function of the unmodeled protein fraction (Q) was used as the coefficient for the "protein_biomass_to_biomass" reaction which took the form of (15).

$$\text{protein\_biomass} + \frac{Q}{1 - Q} \cdot \text{unmodeled\_protein\_biomass} \rightarrow (1 + \frac{Q}{1 - Q}) \cdot \text{biomass} \tag{15}$$

Braun's Lipoprotein Demand

To reflect the structural demands of the cell membrane, a Braun's lipoprotein demand was implemented to ensure that the most abundant lipoprotein –

EG10544 (b1677) from the *Lpp* gene – and membrane murein are produced according to the following ratio:

$$0.013894 \text{ murein } + \ 0.003597 \text{ lipoprotein b1677} \longrightarrow \emptyset \tag{16}$$

as described by Liu *et al.* 2014 [2]. Here, the reaction was bounded by the growth rate (μ) and the murein used was 'murein5px4p_p'.

Lipoprotein Reactions

Lipoprotein biogenesis reactions were added so that 14 lipoproteins from *i*JL1678-ME (Liu *et al.* 2014 [2]) could be created. In *i*JL1678-ME, lipoproteins can be modified by one of seven phosphatidylglycerol metabolites. It is further modified by either a phosphatidylethanolamine or a phosphatidylglycerol, requiring three enzymatic reactions. These reactions were implemented as one post-translational reaction:

$$\left\{ \begin{array}{c} Protein \\ +\ PG_{metabolite} \\ +\ Phospholipid \end{array} \right\} \left[\begin{array}{c} 1\ of\ 14 \\ 1\ of\ 7 \\ PG\ or\ PE \end{array} \right] \longrightarrow \left\{ \begin{array}{c} Lipoprotein \\ +\ G3P \\ +\ 2ag(phospholipid)_{PG\ or\ PE} \end{array} \right\} \tag{17}$$

which is catalyzed by the enzymes: EG10168 , Lgt, and LspA. The total number of reactions added to the model is 196 (14 × 7 × 2), in which 14 new lipoproteins are produced and can be used in downstream metabolic reactions and protein complex formations.

Model Corrections, Improvement and Additions

Dummy Complex / Orphan Reactions

Not all metabolic reactions modeled in *i*JL1678b-ME are annotated with the known enzyme complex that catalyzes the metabolic conversion. Previous ME-model reconstructions have modeled such reactions effectively as spontaneous reactions with no enzyme complex coupled to metabolic flux. For ME-models constructed using COBRAme it is assumed that, if the metabolic reaction does not have an associated catalytic enzyme and is not explicitly annotated as a spontaneous reaction (i.e., orphan reactions), there is a protein cost to operating the reaction. This is accomplished by coupling flux through the orphan reaction to the creation of a “dummy” complex with

coupling amount of $\frac{\mu}{k_{eff}}$ as in other metabolic reactions. The "dummy" complex is composed of a single equivalent of a "dummy" protein with a codon composition representative of an average gene found in *E. coli.*

Modeling of Protein Carriers

Some proteins in *E. coli* act by donating side groups to other proteins or metabolites (i.e., carriers). In previous ME-models, these reactions were modeled as

$$\text{protein\_mod} + \text{metabolite} \rightarrow \text{protein} + \text{metabolite\_mod} \tag{18}$$

where "mod" represents a generic side group that is donated from the protein to the metabolite. As a consequence, in some cases the *i*OL1650 ME-model did not require the protein to be translated in order for the reaction to proceed. The new ME-model formulation identifies all such cases and models the process as

$$\left(1 + \frac{\mu}{k_{eff}}\right) \text{protein\_mod} + \text{metabolite} \rightarrow \text{protein} + \text{metabolite\_mod} \tag{19}$$

This effectively accounts for the metabolic cost of producing and donating the side group ("mod") while also requiring a portion of the protein_mod complex to be diluted. As a result of this change 52 more genes must be expressed in *i*JL1678b-ME compared to it predecessor.

Solve Procedure Simplification

Previous ME-model versions required that the user had previous knowledge of the growth regime that the *E. coli* cell would be growing in prior to solving. In other words, the user had to know whether, for the determined growth environment, simulated growth was nutrient limited or proteome limited. Previously, if simulating substrate limited growth, a two step procedure was required. This included 1) performing binary search to determine the maximum growth rate μ^* and 2) performing a minimization on:

$$\min_{y} \; ke\vec{f}f^* = y \cdot \vec{k_{eff}} \tag{20}$$

where y is a scaling term with values between 0 and 1 to represent the global enzyme effective rate constant vector ($\vec{k_{eff}}$) acting below optimality. This

was done to produce a realistic flux state that accounts for the observation that enzymes in a cell undergoing nutrient limited growth act below optimality (O'Brien et al. 2013 [1]).

To circumvent the need to perform two subsequent optimizations in nutrient limited growth, the presented ME-model optimizes for the production of a "dummy" complex while solving each LP throughout the binary search procedure. The two approaches give identical results since producing extra protein is computationally identical to globally reducing enzyme efficiency.

As a result, the following procedure was implemented in COBRAme to perform the binary search. First, each symbolic coefficient or reaction bound was compiled into a function by SymPy. Then, a linear program was created and passed into the linear programming solver, with all of these symbolic functions evaluated to an initial μ value. For each instance of the binary search in μ, values in the linear program were replaced by the new μ value, and the problem was resolved using the last feasible basis and optimizing for the production of a "dummy" complex. This was repeated until the difference between the maximum feasible and minimum infeasible μ computed was within a predetermined tolerance/precision chosen by the user (See **ME-model Characteristics** for more on this).

Model Updates and Corrections

No new gene content was added to the model, but several changes were made to *i*JL1678-ME if it corrected model errors, reduced the number of required artificial metabolite sources or sinks, or simplified the model. For example, previous ME-models included a forced 1:1 flux split constraint between the reactions catalyzed by the two NADH dehydrogenase complexes, NDHI (NADH-DHI-CPLX) and NDHII (NADH-DHII-MONOMER). This was applied in *i*JL1678-ME to give solutions with P/O ratios within the measured range. This was removed to reduce the number of artificial constraints imposed on the model and since the flux split will not be generally observed in all experimental conditions.

New modification SubReactions were added to the model to reduce the number of "free" modifications that could take place without first synthesizing the machinery or metabolites needed for them to occur. For example, a modification SubReaction to produce a glycyl radical was added to correctly model the activity of the PflA enzyme, an enzyme included in *i*JL1678-ME that not have any functionality. This modification is required to activate PflB and TdcE. Additionally ribosomal complex Rpl7/12 requires a modification of an acetyl group, which is a modification with a known stoichiom-

etry [3]. This modification was added as a new modification SubReaction (mod_acetyl_c). A feasible stoichiometry for NiFeCoCN2 formation [4], a metabolite required for formate dehydrogenase activity, was also added to the model as a modification SubReaction. This allowed the removal of an artificial source of this metabolite from the model. Lastly, there were a few complexes with incorrect prosthetic group annotations. This included cytochrome bo3 ubiquinol oxidase (CYT-O-UBIOX-CPLX) missing a hemeO modification annotation and aminodeoxychorismate lyase (ADCLY-CPLX) incorrectly associated with a pyridoxal (pydx) modification instead of the correct pyridoxal 5'-phosphate (pydx5p) modification.

An effort was made to reduce the number of gaps and pseudo metabolites that were present in *i*JL1678-ME. In order to produce a solving model, many of these gap/pseudo metabolites were given artificial sources or sinks to account for their inability to be synthesized. One example of this was complex NDHII which requires Cu(I) as a coenzyme in order to function. Since Cu(I) synthesis was previously not included in *i*JL1678-ME, Cu(I) was given an artificial source reaction. NDHII, however, has known cupric reductase activity [5] which uses NADH to reduce Cu(II) to Cu(I). Adding this reaction to the model removed the requirement for a Cu(I) source. Additional pseudo metabolites were allowed to freely exchanged into *i*JL1678-ME which represented new metabolites or metabolites already present in the model, but with different IDs. This includes pseudo-metabolites C10H8O5 and C9H9O4 which were included in the cmo5U_at_34 tRNA modification stoichiometry. The stoichiometry was corrected per [6, 7, 8] which negated the need for these metabolites to model this modification. Further, fldrd, fldox, trdox, trdrd were pseudo-metabolites included in *i*JL1678-ME that were able to be replaced with their corresponding flavodoxin or thioredoxin complexes.

The iJO1366 biomass objective function was completely removed from *i*JL1678-ME, except for glycogen. This is due to the fact that most of the components (amino acids, nucleotides, etc.) are already required to be synthesized in order to form macromolecules in the ME-model. Some metabolites, such as coenzyme A, however, can be recycled and must be drained from the model via a biomass objective function-type reaction in order for the pathways which synthesize these metabolites to be active. To accomplish this, metabolites similar to coenzyme A were added to a reaction, called "biomass_constituent_demand" with a coefficient equivalent to the number used in the *i*JO1366 biomass objective function. These metabolites included glycogen, 2-octaprenyl-6-hydroxyphenol, nicotinamide adenine dinucleotide, undecaprenyl diphosphate, coenzyme A, riboflavin, nicoti-

namide, adenine dinucleotide phosphate, 5,10-methylenetetrahydrofolate, 5,6,7,8-tetrahydrofolate, and 10-formyltetrahydrofolate. The lower and upper bound of this reaction was constrained to equal μ to ensure all the metabolites are produced at levels equivalent to *i*JO1366. Further, the biomass created as a result of synthesizing the components was accounted for (similar to proteins, RNA, etc.) with a "biomass_constituent_biomass" constraint/metabolite.

Some enzymes complexes require the synthesis of large prosthetic groups or the incorporation of coenzymes in order to carry their enzymatic function. To account for the biomass of these components, *i*JL1678-ME was updated to include "prosthetic_group_biomass" constraints. These constraints are handled identically to the "protein_biomass", "rna_biomass", etc. constraints. Similarly, constraints were added which account for the biomass produced by lipids, peptidoglycans, DNA, and the remaining biomass constituents (described above).

Lastly, corrections were made to *i*JL1678-ME to remove metabolites from the *in silico* growth media that are not present in minimal *E. coli* growth media. For instance cob(I)alamin is not essential for growth in *E. coli*, but was required in the *in silico* growth media of *i*JL1678-ME to produce a feasible model. This was due to cob(I)alamin being included in *i*JL1678-ME as an essential modification for the QueG complex. It has been shown that the presence of cob(I)alamin increases the epoxyqueuosine reductase tRNA modification activity, but is not necessarily required for the reaction to take place [9]. Alternatively, a known failure mode of *i*OL1678-ME and *i*JL1678-ME is that all RNA modification genes are computationally essential, when this is not always the case *in vivo*. This means that the tRNA modification catalyzed by QueG is computationally essential. Given that the presence of cob(I)alamin in the *in silico* media will allow the activity of three reactions that would not be active when grown in M9 minimal media (METS (catalyzed by MetH), ETHAAL, and MMM), the cob(I)alamin modification was removed from QueG and replaced with the two known iron-sulfur cluster modifications. Biotin was also removed from the *in silico* growth media since *i*JL1678b-ME can synthesize it from glucose and therefore it does not need to be supplemented for growth.

The remaining minor model corrections are summarized in *corrections.py* and *iJL1678b_model_changes.xlsx* in ECOLIme.

Parameter Updates and Corrections

The parameter corresponding to the molecular mass of the RNA component of a ribosome was adjusted. The value of 1700 kDa used in O'Brien *et al.* [1] resulted in an under-abundance of rRNAs synthesized in the simulation. This parameter value was replaced with 1453 kDa, which is the sum of the average molecular weights of the 5S, 16S, and 23S rRNAs modeled in iJL1678b.

The metabolic reaction k_{eff}s were set with an average of 65 s^{-1} and scaled by their solvent accessible surface area (approximated as the complex's moleculare_weight$^{\frac{3}{4}}$). A further set of 284 metabolic k_{eff}s were found by Ebrahim *et al.* [10] to be particularly important in *E. coli* for computing an accurate metabolic/proteomic state using proteomics data. These 284 k_{eff}s were used in iJL1678b-ME with the upper and lower limit of the k_{eff} values constrained to 3000 and 0.01, respectively. This was done to keep the vector of k_{eff} values within a physiologically feasible range.

ME-model Characteristics

One key characteristic of ME-models is that they are self limiting. This means that regardless of the amount of *in silico* nutrients (i.e. glucose) available to the model, there will be one maximum feasible growth rate predicted by the model (**Fig. A**). This results in three growth regions: the **nutrient limited region** where growth increases linearly with increasing glucose availability, the **batch growth region** where increasing glucose has no effected on growth rate, and a transition region (called the "**Janusian region**" in [1]) between the two which results from the limited proteome availability beginning to constrain the simulation. Of note, the transition from nutrient limited growth to batch growth (the "Janusian region") is more abrupt for iJL1678b-ME than iOL1650-ME [1]. This is seen because the iJL1678b-ME simulations are ran using the metabolic reaction k_{eff} vector from Ebrahim et al. [10]. As a result, the model does not predict acetate overflow metabolism at the maximum feasible growth rate in glucose batch growth conditions. This transition to acetate overflow metabolism was largely responsible for the extended "Janusian region" in iOL1650-ME. While the k_{eff} vector used for iJL1678b-ME is generally much more predictive than those used for iOL1650-ME [10], this limitation highlights the need for further examination of these kinetic constraints on a systems level.

As mentioned above, underlying this transition between nutrient limited growth and batch growth is caused by a restriction in metabolic pro-

cesses as a result of the proteome constraints inherent in the model. One way to visualize these constraints taking effect is to observe the changes in their dual values (shadow prices) relative to a biological function with increasing growth rates (shown for ATP synthesis in **Fig. A**). As the proteome constraints begin to restrict the model around a glucose uptake rate of 10 $\frac{mmol}{gDW \cdot hr}$, for instance, the "protein_biomass" constraint increases. This means alleviating this constraint would increase the amount of ATP the cell is capable of producing.

Another characteristic of ME-models is that their accuracy depends on the precision of of the maximum feasible growth rate obtained from binary search (see **Solve Procedure Simplification**). Since this value is determined using a binary/bisection algorithm, the solve procedure requires that the minimum acceptable difference between maximum computed feasible growth rate and minimum infeasible growth rate is set as a parameter. The more precise the growth rate value, the less variability possible in a particular reaction. In addition to PGI in **Figure 4**, the effect of μ precision on flux variability was observed for MGSA (Methylglyoxal synthase) and E4PD (Erythrose 4-phosphate dehydrogenase) along with the transcription and translation reactions required to express the enzymes that catalyze these reactions (**Fig. B**). MGSA is a nonessential reaction that carries effectively no flux at the maximum feasible growth rate when grown on glucose. E4PD can be catalyzed by two isozymes and carries low flux at the maximum feasible growth rate. The trends seen for PGI are generally observed for these two reactions where the variability effectively disappears when solving with a μ precision of 10^{-15}. As with PGI, maximum possible reaction flux for MGSA and the reverse direction of E4PD does not drop to $10^{-15}\frac{mmol}{gDW \cdot hr}$ by this μ precision. Their maximum value does, however, drop to below $10^{-13}\frac{mmol}{gDW \cdot hr}$ which is orders of magnitude below the lowest metabolic flux shown in **Figure 5**

Supplementary Figures

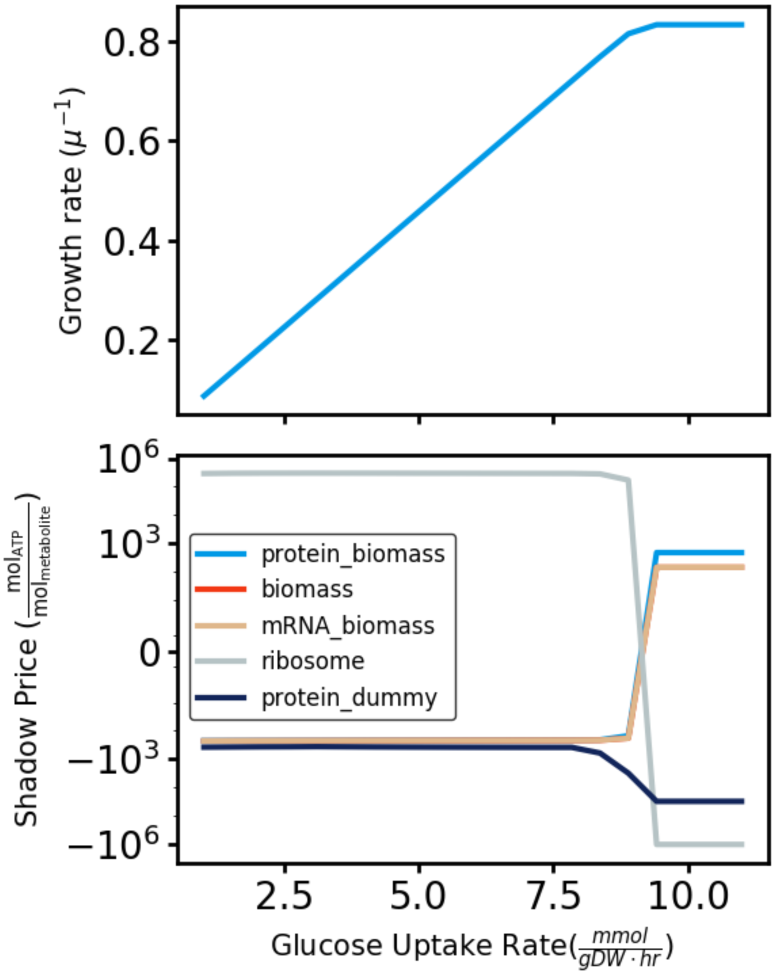

Fig. A: The iJL1678-ME Growth Curve. In the top panel the computed growth rate is plotted as a function of glucose uptake. The bottom panel depicted the role of global ME-model constraints in the transition from nutrient limited to batch growth, as indicated by the change in their shadow prices.

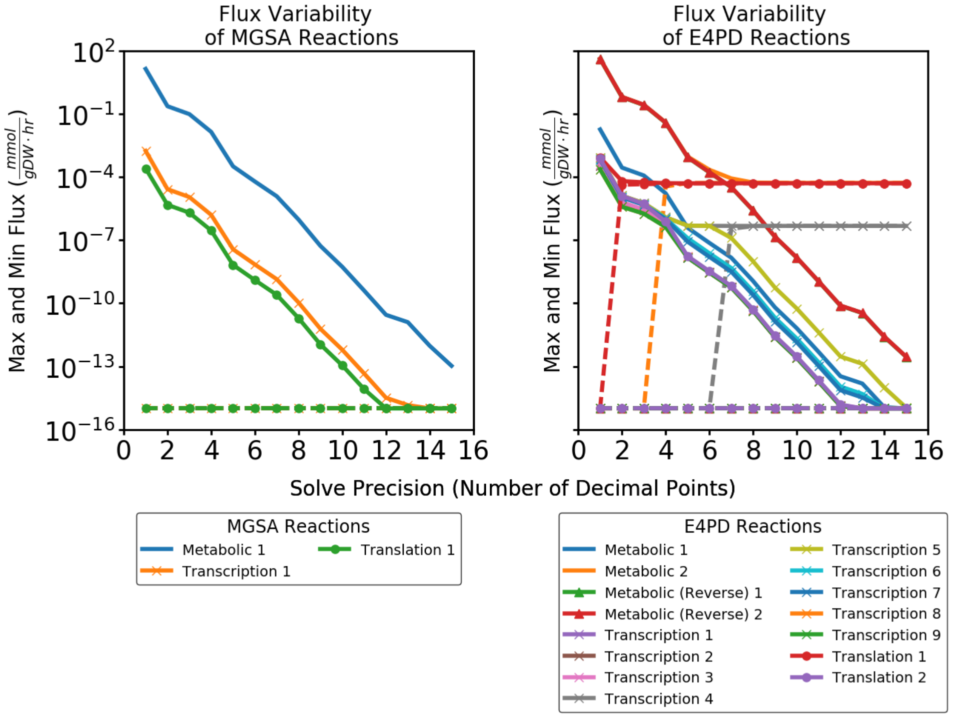

Fig. B: The effect of solver precision on flux variability of reactions MGSA and E4PD along with the reactions required to synthesize their enzymes.

References

[1] Edward J O'Brien, Joshua A Lerman, Roger L Chang, Daniel R Hyduke, and Bernhard Ø Palsson. Genome-scale models of metabolism and gene expression extend and refine growth phenotype prediction. *Mol. Syst. Biol.*, 9:693, 2013.

[2] Joanne K Liu, Edward J O Brien, Joshua A Lerman, Karsten Zengler, Bernhard O Palsson, and Adam M Feist. Reconstruction and modeling protein translocation and compartmentalization in escherichia coli at the genome-scale. *BMC Syst. Biol.*, 8(1):110, September 2014.

[3] Lin Miao, Hongqing Fang, Yanying Li, and Huipeng Chen. Studies of the in vitro nalpha-acetyltransferase activities of e. coli RimL protein. *Biochem. Biophys. Res. Commun.*, 357(3):641–647, June 2007.

[4] Sven T Stripp, Basem Soboh, Ute Lindenstrauss, Mario Braussemann, Martin Herzberg, Dietrich H Nies, R Gary Sawers, and Joachim Heberle. HypD is the scaffold protein for Fe-(CN)2CO cofactor assembly in [NiFe]-hydrogenase maturation. *Biochemistry*, 52(19):3289–3296, May 2013.

[5] V A Rapisarda, L R Montelongo, R N Farías, and E M Massa. Characterization of an NADH-linked cupric reductase activity from the escherichia coli respiratory chain. *Arch. Biochem. Biophys.*, 370(2):143–150, October 1999.

[6] Jungwook Kim, Hui Xiao, Jeffrey B Bonanno, Chakrapani Kalyanaraman, Shoshana Brown, Xiangying Tang, Nawar F Al-Obaidi, Yury Patskovsky, Patricia C Babbitt, Matthew P Jacobson, Young-Sam Lee, and Steven C Almo. Structure-guided discovery of the metabolite carboxy-SAM that modulates tRNA function. *Nature*, 498(7452):123–126, June 2013.

[7] Jungwook Kim, Hui Xiao, Junseock Koh, Yikai Wang, Jeffrey B Bonanno, Keisha Thomas, Patricia C Babbitt, Shoshana Brown, Young-Sam Lee, and Steven C Almo. Determinants of the CmoB carboxymethyl transferase utilized for selective tRNA wobble modification. *Nucleic Acids Res.*, 43(9):4602–4613, May 2015.

[8] Yusuke Sakai, Kenjyo Miyauchi, Satoshi Kimura, and Tsutomu Suzuki. Biogenesis and growth phase-dependent alteration of 5-

methoxycarbonylmethoxyuridine in tRNA anticodons. *Nucleic Acids Res.*, 44(2):509–523, January 2016.

[9] Zachary D Miles, Reid M McCarty, Gabriella Molnar, and Vahe Bandarian. Discovery of epoxyqueuosine (oq) reductase reveals parallels between halorespiration and tRNA modification. *Proc. Natl. Acad. Sci. U. S. A.*, 108(18):7368–7372, May 2011.

[10] Ali Ebrahim, Elizabeth Brunk, Justin Tan, Edward J O'Brien, Donghyuk Kim, Richard Szubin, Joshua A Lerman, Anna Lechner, Anand Sastry, Aarash Bordbar, Adam M Feist, and Bernhard O Palsson. Multi-omic data integration enables discovery of hidden biological regularities. *Nat. Commun.*, 7:13091, October 2016.
